# Genetic insights into the social organization of Neanderthals

**DOI:** 10.1038/s41586-022-05283-y

**Published:** 2022-10-19

**Authors:** Laurits Skov, Stéphane Peyrégne, Divyaratan Popli, Leonardo N. M. Iasi, Thibaut Devièse, Viviane Slon, Elena I. Zavala, Mateja Hajdinjak, Arev P. Sümer, Steffi Grote, Alba Bossoms Mesa, David López Herráez, Birgit Nickel, Sarah Nagel, Julia Richter, Elena Essel, Marie Gansauge, Anna Schmidt, Petra Korlević, Daniel Comeskey, Anatoly P. Derevianko, Aliona Kharevich, Sergey V. Markin, Sahra Talamo, Katerina Douka, Maciej T. Krajcarz, Richard G. Roberts, Thomas Higham, Bence Viola, Andrey I. Krivoshapkin, Kseniya A. Kolobova, Janet Kelso, Matthias Meyer, Svante Pääbo, Benjamin M. Peter

**Affiliations:** 1 Department of Evolutionary Genetics, Max Planck Institute for Evolutionary Anthropology, Leipzig, Germany; 2 European Centre for Research and Education in Environmental Geosciences (CEREGE), Aix-Marseille University, CNRS, IRD, INRAE, Collège de France, Aix-en-Provence, France; 3 Department of Anatomy and Anthropology Sackler, Faculty of Medicine, Tel Aviv University, Tel Aviv, Israel; 4 The Dan David Center for Human Evolution and Biohistory Research, Tel Aviv University, Tel Aviv, Israel; 5 Department of Human Molecular Genetics and Biochemistry, Sackler Faculty of Medicine, Tel Aviv University, Tel Aviv, Israel; 6 The Francis Crick Institute, London, UK; 7 Wellcome Sanger Institute, Hinxton, UK; 8 Oxford Radiocarbon Accelerator Unit, Research Laboratory for Archaeology and the History of Art, University of Oxford, Oxford, UK; 9 Institute of Archaeology and Ethnography, Russian Academy of Sciences, Novosibirsk, Russia; 10 Department of Chemistry G. Ciamician, Alma Mater Studiorum, University of Bologna, Bologna, Italy; 11 Department of Human Evolution, Max Planck Institute for Evolutionary Anthropology, Leipzig, Germany; 12 Department of Evolutionary Anthropology, Faculty of Life Sciences, University of Vienna, Vienna, Austria; 13 Department of Archaeology, Max Planck Institute for the Science of Human History, Jena, Germany; 14 Human Evolution and Archaeological Sciences Forschungsverbund, University of Vienna, Vienna, Austria; 15 Institute of Geological Sciences, Polish Academy of Sciences, Warsaw, Poland; 16 Centre for Archaeological Science, School of Earth, Atmospheric and Life Sciences, University of Wollongong, Wollongong, New South Wales Australia; 17 Australian Research Council (ARC) Centre of Excellence for Australian Biodiversity and Heritage, University of Wollongong, Wollongong, New South Wales Australia; 18 Department of Anthropology, University of Toronto, Toronto, Ontario Canada

**Keywords:** Genetic variation, Evolutionary biology, Human behaviour, Evolutionary genetics, Anthropology

## Abstract

Genomic analyses of Neanderthals have previously provided insights into their population history and relationship to modern humans^1–8^, but the social organization of Neanderthal communities remains poorly understood. Here we present genetic data for 13 Neanderthals from two Middle Palaeolithic sites in the Altai Mountains of southern Siberia: 11 from Chagyrskaya Cave^9,10^ and 2 from Okladnikov Cave^11^—making this one of the largest genetic studies of a Neanderthal population to date. We used hybridization capture to obtain genome-wide nuclear data, as well as mitochondrial and Y-chromosome sequences. Some Chagyrskaya individuals were closely related, including a father–daughter pair and a pair of second-degree relatives, indicating that at least some of the individuals lived at the same time. Up to one-third of these individuals’ genomes had long segments of homozygosity, suggesting that the Chagyrskaya Neanderthals were part of a small community. In addition, the Y-chromosome diversity is an order of magnitude lower than the mitochondrial diversity, a pattern that we found is best explained by female migration between communities. Thus, the genetic data presented here provide a detailed documentation of the social organization of an isolated Neanderthal community at the easternmost extent of their known range.

## Abstract (web summary)

Genetic data for 13 Neanderthals from 2 Middle Palaeolithic sites in the Altai Mountains of southern Siberia presented provide insights into the social organization of an isolated Neanderthal community at the easternmost extent of their known range.

## Main

Neanderthals occupied western Eurasia from around 430,000 years ago^8,12^ until their extinction around 40,000 years ago^13^. Genome-scale data have been reported for the skeletal remains of 18 individuals from 14 archaeological sites^1–8^ spanning Neanderthal history across large parts of their known geographical range, which extends as far east as the Altai Mountains in southern Siberia. These data have yielded a broad overview of Neanderthal populations, indicating the existence of multiple distinct Neanderthal populations over time and space^1,2,14^.

However, little is known about the genetic relationships and social organization within and between Neanderthal communities in any part of Eurasia during this time interval.

By ‘social organization’, we mean the size, sex composition and spatiotemporal cohesion of a community^15^. We define a community as a set of individuals that presumably lived together at the same location, and reserve the term population for a broadly connected set of communities in a wider geographical area.

On the basis of fossilized footprints^16,17^ and spatial patterns of site use^18^, previous studies on the social organization of Neanderthal communities have suggested that Neanderthals probably lived in small communities. In addition, partial mitochondrial DNA (mtDNA) sequences from six adult Neanderthals have been used to suggest that Neanderthals may have been patrilocal^19^, although this suggestion has been debated^20^.

**Fig. 1:**
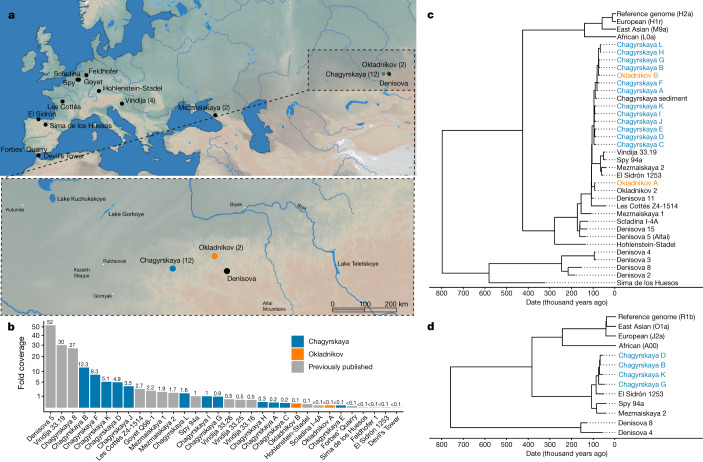
Neanderthal sites and genomic information. **a**, Locations of all of the sites with Neanderthal remains (the number of individuals is given in parentheses for sites with multiple individuals) from whom nuclear DNA has been extracted, with a close-up of the Chagyrskaya and Okladnikov caves in the Altai region of southern Siberia. **b**, Nuclear genomes ranked by the extent of coverage and colour-coded by site (blue, Chagyrskaya from this study; orange, Okladnikov from this study; grey, published previously in refs. ^1–8^). **c**, Maximum-likelihood tree for mtDNA sequences from the Neanderthal individuals included in this study in the context of known hominin variation. The reference genome is rCRS and the accession numbers for the present-day humans are East Asian (AF346973), European (AF346981) and African (AF381988). Okladnikov 2 refers to the mtDNA sequence in ref. ^41^ (this specimen is listed as Okladnikov 14 in Extended Data Table 1). Data from refs. ^1–4,6,30,41,42–49^. **d**, Maximum-likelihood tree based on consensus calling of 6.9 Mb of the Y chromosome of four Chagyrskaya individuals with coverage of more than onefold, along with previously published Y-chromosome data from three Neanderthals, two Denisovans and four present-day humans. The reference genome is hg19. Data from refs. ^26,50–53^. In **c** and **d**, the haplogroups are shown for present-day human populations.

Here we explore the social organization of Neanderthals using nuclear, Y-chromosomal and mtDNA data from the remains of 13 individuals recovered from 2 sites located close to one another in southern Siberia (Russia)—Chagyrskaya and Okladnikov caves (Table 1 and Fig. 1a).

**Table 1:** Neanderthals from Chagyrskaya and Okladnikov Caves included in this study

Individual	Bone/tooth ID	Age	Anatomical element	Genetic sex	Relationship to other individual(s)
Chagyrskaya A	Chagyrskaya 1	8–12 (D)	Deciduous lower left canine	Male	Second-degree relation of Chagyrskaya L
Chagyrskaya B	Chagyrskaya 2	3–5	Atlas (first cervical vertebra)	Male	
Chagyrskaya C	Chagyrskaya 6	Adult	Right mandible fragment with canine to M_2_	Male	
Chagyrskaya C	Chagyrskaya 14	Adult	Lower left second incisor	Male	
Chagyrskaya D	Chagyrskaya 7	Adult?	Thoracic vertebral process fragment	Male	Father of Chagyrskaya H; possible first-degree relation of/identical to Chagyrskaya E
Chagyrskaya E?	Chagyrskaya 9	Adult	Left proximal ulna fragment	Male	Possible first-degree relation of/identical to Chagyrskaya D
Chagyrskaya F	Chagyrskaya 12	Adult	Left third premolar	Female	
Chagyrskaya F	Chagyrskaya 8^a^	Adult	Distal phalanx of the hand (high-coverage genome)	Female	
Chagyrskaya G	Chagyrskaya 13	10–15	Left upper first incisor	Male	
Chagyrskaya G	Chagyrskaya 19	9–11 (D)	Deciduous left upper second molar	Male	
Chagyrskaya G	Chagyrskaya 63	9–14	Upper left second molar crown	Male	
Chagyrskaya H	Chagyrskaya 17	15–20?	Right lower fourth premolar	Female	Daughter of Chagyrskaya D
Chagyrskaya I	Chagyrskaya 18	9–11 (D)	Deciduous left upper M^1^	Female	
Chagyrskaya J	Chagyrskaya 20	7–12 (D)	Deciduous right upper canine	Female	
Chagyrskaya K	Chagyrskaya 41	Adult	Right lower third premolar	Male	
Chagyrskaya L	Chagyrskaya 60	Adult	Middle phalanx of the hand	Female	Second-degree relation of Chagyrskaya A
Okladnikov A	Okladnikov 11	7–11	Proximal half of a juvenile femur	Male	
Okladnikov B	Okladnikov 15	Adult	Right distal humerus fragment	Female	

Ages represent age-at-death estimates based on anatomical features, with the exception of the deciduous teeth (D); for these naturally exfoliated teeth, age is the time of tooth loss. Details are provided in Supplementary Information section 1.

^a^A high-coverage genome for Chagyrskaya 8 has been published previously^5^.

## Archaeological sites and remains

**Extended Data Fig. 1:**
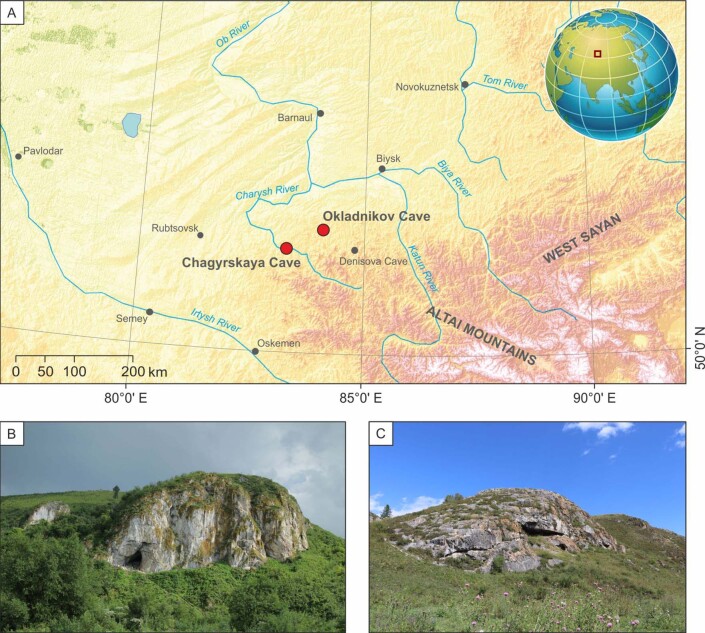
Chagyrskaya and Okladnikov Caves. **A**, Location map of Chagyrskaya and Okladnikov Caves in the Altai region of southern Siberia. Views of the **B**, north-facing entrance to Chagyrskaya Cave and **C**, south-facing entrance to Okladnikov Cave.

**Extended Data Fig. 2:**
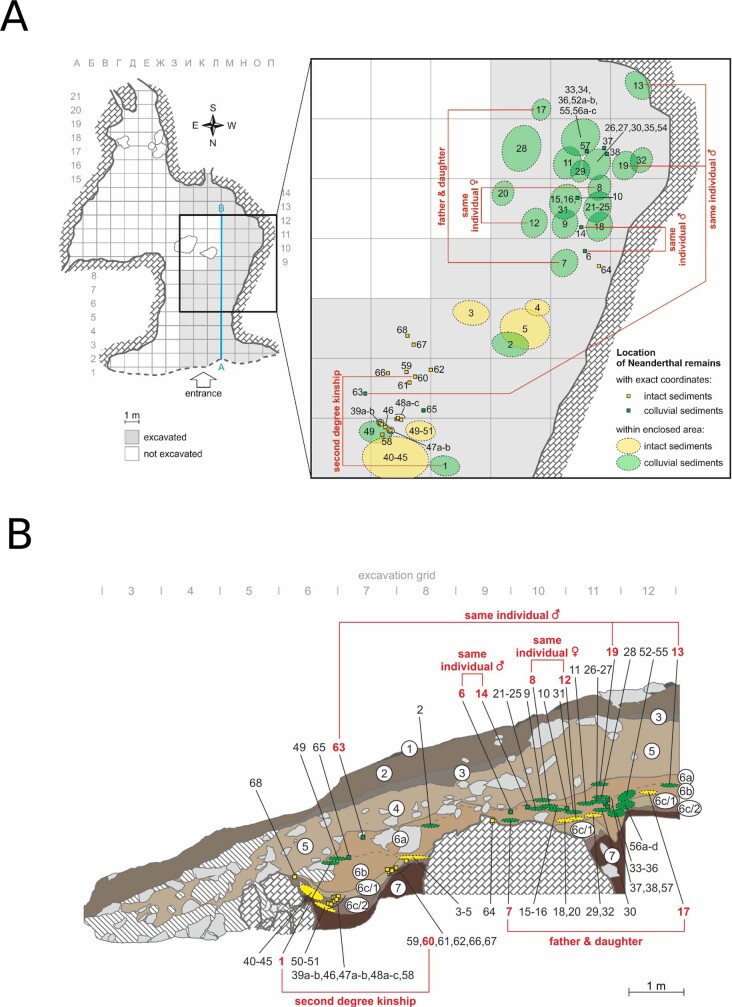
Plan map of Chagyrskaya Cave and locations of Neanderthal remains. **A**, Spatial distribution of Neanderthal remains. The excavated area is shown in grey, and the blue line (transect A–B) marks the position of the stratigraphic profile shown in **B**. The coloured squares and ellipses denote Neanderthal remains located with exact coordinates or within the circumscribed areas, respectively, and are annotated with the corresponding fossil number(s). **B**, Stratigraphic profile along transect A–B in **A**. Locations of Neanderthal remains are projected orthogonally onto this profile, so each fossil is not necessarily shown in the stratigraphic unit from which it was recovered.

The Chagyrskaya and Okladnikov caves, located in the foothills of the Altai Mountains (Fig. 1a and Extended Data Figs. 1 and 2), are thought to have been used mainly as short-term hunting camps^11,21^. They are two of three known sites at which a distinctive Sibiryachikha Middle Palaeolithic industry has been found (the third being Upper Sibiryachikha Cave)^9,10,22,23^ (Supplementary Fig. 1.6). The Sibiryachikha industry at Chagyrskaya and Okladnikov caves is distinct from the Middle Palaeolithic industry at Denisova Cave (located around 100 km to the east), where Neanderthal remains have also been found^2^.

The Neanderthal occupation deposits at Chagyrskaya Cave accumulated between 59,000 and 51,000 years ago, as indicated by optical dating of sediments and radiocarbon dating of bison bones^10^. We obtained additional radiocarbon ages from two pieces of charcoal and a Neanderthal bone (Chagyrskaya 9), all of which were older than 50,000 years before present (Supplementary Table 1.3). These ages are compatible with a short period of deposition (a few millennia or less), which is consistent with the presence of similar archaeological industry in all Neanderthal layers^10^ (Extended Data Fig. 2).

For Okladnikov Cave, we constrained the timing of Neanderthal occupation using hydroxyproline-based single amino-acid radiocarbon ages for three Neanderthal specimens (including Okladnikov 15) (Table 1 and Extended Data Table 1), which indicated that they were at least 44,000 years old (Supplementary Table 1.4). Our age estimates are consistent with uranium-series ages for animal bones and support previous suggestions that younger radiocarbon ages obtained from the collagen fraction reflect an incomplete removal of contaminants^24^ (Supplementary Information section 1). Therefore, the archaeological and chronological data suggest that the Neanderthals that occupied these two sites may have been broadly contemporaneous.

Previous analyses of high-coverage genomes of a Neanderthal from Chagyrskaya Cave (Chagyrskaya 8) and an earlier Neanderthal from Denisova Cave (Denisova 5, the ‘Altai Neanderthal’) revealed that they belonged to different populations^5^. A first-generation offspring (Denisova 11) of a Neanderthal mother and a Denisovan father revealed that the Neanderthal mother was more similar to Chagyrskaya 8 than she was to other Neanderthals^5,25^.

## Data acquisition and sex determination

We sampled 1–64 mg of tooth or bone powder from 17 specimens from Chagyrskaya Cave and 10 specimens from Okladnikov Cave. Of these, 15 from Chagyrskaya and 2 from Okladnikov yielded ancient DNA (Table 1, Extended Data Table 1 and Supplementary Data 1), from which we generated a total of 85 single-stranded DNA libraries (Supplementary Information section 2). All of the libraries were enriched for mtDNA sequences (Supplementary Information section 3) and 49 libraries (selected for high sequence yields and low levels of present-day human contamination) were enriched for nuclear DNA using a newly designed nuclear-capture array containing 643,472 transversion polymorphisms across the genome (Supplementary Information section 5). In the array, 271,306 sites vary among the 4 published high-coverage archaic individuals (three Neanderthals and one Denisovan)^2,3,5,14^ and 372,166 sites segregate in present-day African populations or are fixed between present-day humans and archaic hominins. The average nuclear DNA coverage for each fossil ranges from 0.04- to 12.3-fold (Fig. 1b), and present-day human contamination estimates range from 0.1% to 3.2% (Supplementary Table 5.4).

We determined the genetic sex of the 17 remains using the difference in coverage between the X chromosome and autosomes (Supplementary Fig. 5.5) and found that 6 remains stemmed from females. For the 11 male remains, we enriched the libraries for around 6.9 megabases (Mb) of Y-chromosome sequence^26^ (Supplementary Information section 4), yielding coverages ranging between 0.02- and 42.2-fold (Supplementary Table 4.3).

## Identification of relatives

To determine whether any of the remains originated from related individuals, we computed the nuclear DNA divergence between the 17 remains by randomly sampling 1 allele from 250,785 sites in the capture array that were variable in the high-coverage archaic individuals (excluding variants specific to Chagyrskaya 8) (Supplementary Information section 5). The divergence will be lower for related individuals because they have inherited parts of their genomes from the ancestors they share in the recent past. We normalized this divergence (*p*_0_) by a median DNA divergence among all comparisons. Using this approach^27^, we can detect up to second-degree relationships; we consider everything beyond that as unrelated. We expect *p*_0_ = 1 for remains who are more distantly related than second-degree relatives, *p*_0_ = 0.875 for second-degree relatives, *p*_0_ = 0.75 for first-degree relatives and *p*_0_ = 0.5 for remains from monozygotic twins or the same individual^27^. We also investigated mtDNA heteroplasmies (that is, when mitochondria carried by an individual differ in their DNA sequence) (Supplementary Table 3.2) to identify close genetic relationships^28^. As heteroplasmies can be transmitted from mother to child and typically persist for less than three generations^29^, their presence in different remains would indicate that they come from the same or maternally closely related individuals. To differentiate between remains (that is, between skeletal and dental samples) and individuals, we denote the former with numbers and the latter with letters (Table 1).

**Extended Data Fig. 3:**
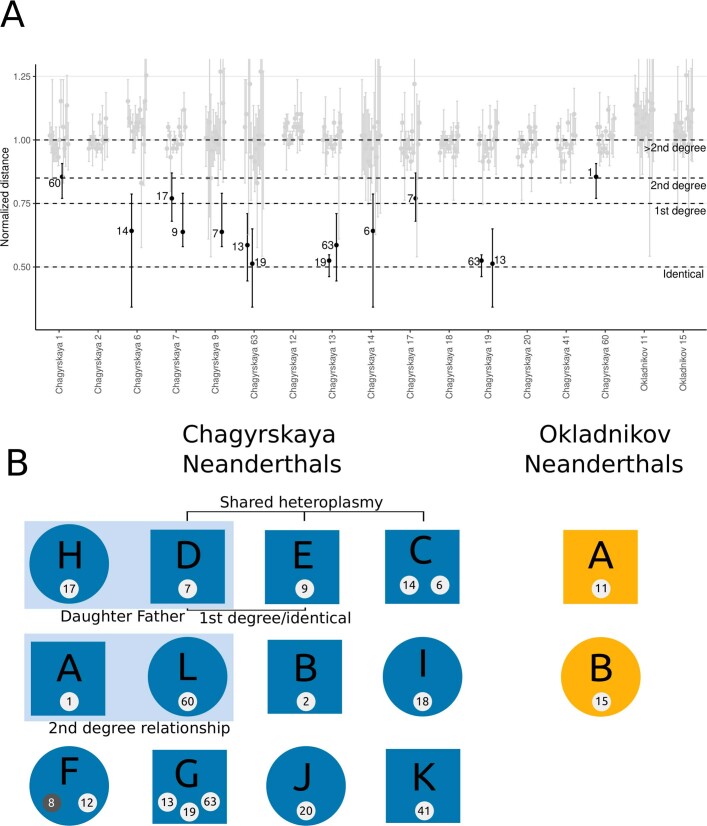
Normalized pairwise differences between Chagyrskaya and Okladnikov remains. **A**, Points show the mean pairwise differences (y-axis) between two remains (normalized by the median difference between all pairs of remains). Remains that were identified as identical, first degree and second degree relatives are named (x-axis shows the first fossil and the number denotes the second remain). Error bars are 95% confidence intervals for 100 bootstrap estimates of the mean pairwise differences. Horizontal lines indicate the expected normalized difference for identical individuals, first degree relationships, second degree relationships and unrelated individuals^27^. **B**, Each circle/square represents an individual (blue for Chagyrskaya, orange for Okladnikov) and the small white circles indicate which remains originated from this individual. The black circle for *Chagyrskaya 8* indicates that the genomic sequence for this bone is previously published. Squares indicate that the individual is male and circles indicate that the individual is female. Individuals which are first degree relatives, second degree relatives or share heteroplasmies are marked.

We found a deciduous tooth (Chagyrskaya 19) and two permanent teeth (Chagyrskaya 13 and Chagyrskaya 63). Surprisingly, despite their different developmental stages, the genetic data suggest that they belonged to the same individual (Chagyrskaya G; average *p*_0_ = 0.53) (Extended Data Fig. 3a). In agreement with this, all three teeth stemmed from a male and carried identical mtDNAs, including a heteroplasmy at position 3,961 at similar frequencies of 60.7–78.5% (Supplementary Table 3.2). The almost completely resorbed root of the deciduous tooth suggests that it was naturally exfoliated (Supplementary Information section 1). On the basis of patterns of wear and root development, we inferred that the permanent teeth came from a 9–15-year-old individual and that this male probably died around the time the deciduous tooth was lost.

We also identified two further sets of individuals with multiple fossils: Chagyrskaya C is represented by both Chagyrskaya 6, a mandible, and Chagyrskaya 14, a permanent incisor (Supplementary Information section 1), as evidenced by the morphological fit, identical mtDNA sequences (including a shared heteroplasmy) and low nuclear divergence (*p*_0_ = 0.65; 95% confidence interval, 0.34–0.78) (Fig. 1c, Extended Data Fig. 3a and Supplementary Tables 3.2 and 7.1). Similarly, Chagyrskaya F is represented by both Chagyrskaya 12 and the previously sequenced^5^ Chagyrskaya 8 (*p*_0_ = 0.46; 95% confidence interval, 0.41–0.46) (Supplementary Table 7.1).

One adult male individual, Chagyrskaya D, was closely related to multiple other individuals in the group. We found a first-degree relationship between him and Chagyrskaya H, who is an adolescent female (*p*_0_ = 0.77; 95% confidence interval, 0.72–0.82). There are three possible male–female combinations for first-degree relatives: mother–son, brother–sister or father–daughter. However, since the two individuals carry different mitochondrial genomes (Fig. 1c), we concluded that Chagyrskaya H was the daughter of Chagyrskaya D.

In addition, his mtDNA was identical to that of two other males, Chagyrskaya C and Chagyrskaya E (Supplementary Table 3.2), including a shared mtDNA heteroplasmy at position 545 (G>A) with a frequency of A of 42–54% for Chagyrskaya D, 20–41% for Chagyrskaya E and 23–30% for Chagyrskaya C. Therefore, these individuals were probably close maternal relatives (for example, they could have shared a grandmother and thus might have been fourth-degree relatives). However, the extent of the relationship between Chagyrskaya C and Chagyrskaya D is beyond the resolution of our approach (*p*_0_ = 1.05; 95% confidence interval, 0.94–1.16). Chagyrskaya E has low coverage (Supplementary Table 5.4) and high amounts of human and nonhuman contamination (Supplementary Table 5.3). After correcting for nonhuman contamination (Supplementary Table 7.1), we identified Chagyrskaya E as either a first-degree relative of or identical to Chagyrskaya D (*p*_0_ = 0.64; 95% confidence interval, 0.48–0.79). As we cannot be confident that Chagyrskaya E is a distinct individual, we removed the sample from further analysis.

The close relationships among Chagyrskaya C, D and H imply that they were contemporaneous. In addition, we found that Chagyrskaya A (male) and L (female) are second-degree relatives (*p*_0_ = 0.85; 95% confidence interval, 0.77–0.91). Although the sparse data prevented us from determining the exact relationship, they must also have lived close in time (Extended Data Fig. 3b). The genetic divergence between each group of contemporaneous individuals and the other six Chagyrskaya individuals were not significantly different from each other (Wilcoxon rank-sum test, both *P* > 0.26) (Supplementary Table 7.4). In addition, the contemporaneous father–daughter pair carried the highest number of differences among all mtDNA sequences, implying that there was no substantial temporal structure in the mtDNA diversity. Taken together, the data supported the hypothesis that all eleven Chagyrskaya Neanderthals were part of the same community.

The two Okladnikov remains were unrelated to each other (*p*_0_ = 1.14; 95% confidence interval, 0.90–1.38) and also not related to any individual from Chagyrskaya Cave. In fact, the pairwise genetic divergence among the Chagyrskaya individuals was lower (*p*_0_ = 1.0; 95% confidence interval, 0.99–1.02) than that between individuals from Chagyrskaya and Okladnikov caves (*p*_0_ = 1.06; Wilcoxon rank-sum test, *P* = 8.6 × 10^−5^) (Extended Data Fig. 3a and Supplementary Table 7.3). This indicates that the Okladnikov Neanderthals were not part of the Chagyrskaya Neanderthal community represented by the 11 individuals for which we obtained DNA. However, the mtDNA of Okladnikov B is identical to that of Chagyrskaya G (Fig. 1c). Because mutations accumulate over time, identical mtDNA between individuals implies that these two individuals lived within a few thousand years of each other (Supplementary Table 3.9). In addition, among the previously published sediment mtDNA samples from Chagyrskaya Cave, 2 of the 38 samples were more similar to Okladnikov A than they were to any Chagyrskaya Neanderthal^30^. This suggests there was some connection between the communities occupying the two caves.

## Relationships to other populations

**Extended Data Fig. 4:**
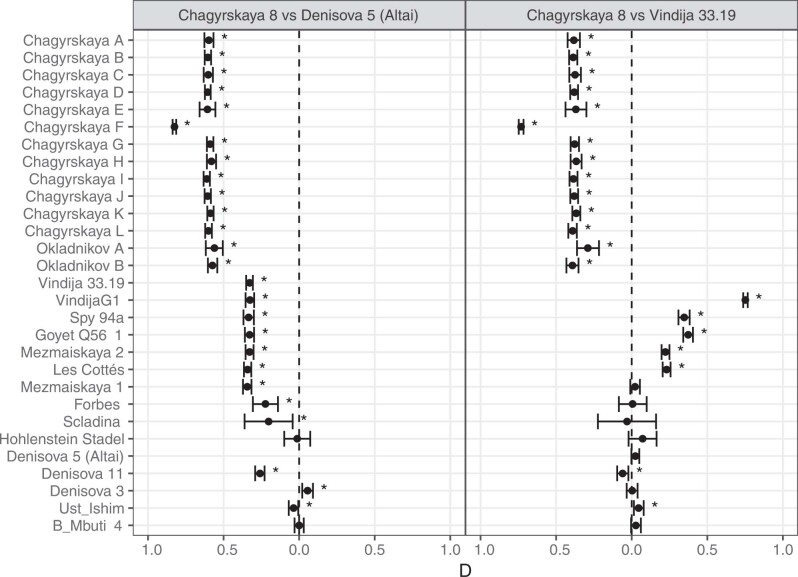
Sharing of variants among archaic genomes. The center of the errorbar show the D-statistic of the form *D*((*Denisova 5*/*Vindija33.19*), *Chagyrskaya 8*; Test, Chimpanzee) and error bars are the corresponding 95% confidence intervals calculated for 643,472 SNPs using a weighted block jackknife and a block size of 5 Mb. Points with |Z-score| > 2 are annotated with an asterisk. The dashed vertical line is at D = 0. Note that *Chagyrskaya F* is the same individual as *Chagyrskaya 8* and *VindijaG1* is the same individual as *Vindija 33.19*.

To explore how the Chagyrskaya and Okladnikov individuals are related to other Neanderthals, we investigated the extent to which they share nucleotide variants with the previously published high-quality Neanderthal genomes. All 13 newly sequenced individuals shared most variants with the high-coverage genome from Chagyrskaya Cave (Chagyrskaya 8)^5^ and were more similar to the around 50,000-year-old Neanderthal genome from Vindija Cave (Vindija 33.19)^3^ in Croatia than to the 91,000–130,000-year-old Altai Neanderthal (Denisova 5) from Denisova Cave^2^ (Extended Data Fig. 4). Therefore, although the communities from Chagyrskaya and Okladnikov caves were genetically distinct, they all appear equally related to European Neanderthals and were part of the same Neanderthal population; no individual showed evidence of recent gene flow from other Neanderthal populations.

We identified 5,416 variants in the 6.9 Mb sequence of the Y chromosome that varied among the Y chromosomes of the seven male individuals, three Neanderthals, two Denisovans and four present-day humans (Supplementary Table 4.7). For three individuals, we obtained only low-coverage sequences (0.03- to 0.3-fold), whereas the other four individuals yielded higher coverages (1.75- to 42.2-fold) (Supplementary Table 4.3).

We constructed a phylogenetic tree that incorporated the four higher-coverage Y-chromosome sequences from Chagyrskaya Cave, along with those of three other Neanderthals, two Denisovans and four present-day humans (Fig. 1d and Supplementary Table 4.7). Among Neanderthals, all four Chagyrskaya sequences form a clade, but they are more similar to El Sidrón 1253 (Spain) than to the geographically closer Mezmaiskaya 2 (northern Caucasus, Russia) (Fig. 1d). This absence of geographical structure is consistent with a fairly rapid expansionof Neanderthals around 100,000–115,000 years ago^30^. Both the late European Neanderthals and the Chagyrskaya and Okladnikov Neanderthals are descendants of this population.

The number of recovered Y-chromosome sequences from the remaining three individuals were not sufficient for constructing a phylogenetic tree, but at positions at which the Neanderthal Y chromosomes differed from each other, all three sequences shared more derived variants with the other Chagyrskaya Y chromosomes than with other Neanderthal Y chromosomes (Supplementary Table 4.9).

**Extended Data Fig. 5:**
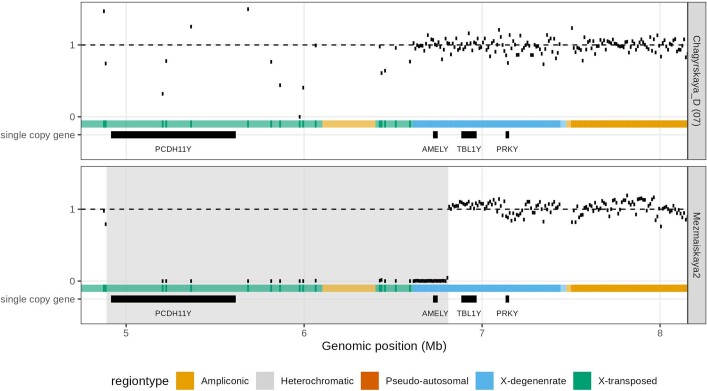
Deletion of the *AMELY* gene on the Y-chromosome. Deletion of 1.8 Mb of sequence on the Y-chromosome of *Mezmaiskaya 2* (bottom panel, light grey) compared to *Chagyrskaya D* (top panel, no deletion). The horizontal axis shows the genomic position on the Y-chromosome and the vertical axis shows the coverage in bins of 10 kb, normalized by the chromosome-wide average coverage. Bin colours indicate the region classes on the human reference Y-chromosome, with darker regions indicating coverage by the Y-chromosome capture array. Black bars denote known coding genes.

On the basis of the differences in coverage in windows of 10 kilobases (kb), we detected 3 deletions and 5 duplications (20–2,000 kb and 10–200 kb in size, respectively) (Supplementary Table 4.4) on the Neanderthal Y chromosomes. The largest deletion was found in Mezmaiskaya 2 and spans the AMELY-encoding gene. Because proteomic approaches use the presence of AMELY peptides to determine whether a bone stems from a male individual^31^, males who carry this deletion would be misclassified as females using this approach (Extended Data Fig. 5).

The mtDNA and Y chromosomes track only single loci, so autosomal genetic analyses are necessary to investigate details of gene flow. Gene flow between Neanderthals and Denisovans in the Altai Mountains has been observed in the nuclear genome of an individual (Denisova 11) who lived 79,000–118,000 years ago and had a Neanderthal mother and a Denisovan father^32^. It has also been estimated that the amount of Denisovan ancestry in Chagyrskaya 8 is around 0.09% and that the admixture event occurred 24,300 ± 14,100 years before Chagyrskaya 8 lived^33^. To investigate whether the timing of admixture is consistent across the other Chagyrskaya individuals, we looked for portions of their genomes that are more similar to the Denisovan genome than to the Altai or Vindija Neanderthals^33^. With this analysis, we identified 11 segments of Denisovan ancestry across 5 Chagyrskaya individuals that are longer than 0.2 centimorgans (cM) (Supplementary Table 6.2). These segments span 3.2 cM (2.7 Mb), with the longest at 1.5 cM (746 kb) found in Chagyrskaya A (Supplementary Fig. 6.2). On the basis of the lengths of these segments, we estimate that the admixture event happened 30,000 ± 18,000 years before the Chagyrskaya individuals lived, which is consistent with the previous estimate (Supplementary Fig. 6.3).

Denisova Cave was occupied by both Neanderthals and Denisovans around the same time that Neanderthals inhabited Chagyrskaya Cave^34,35^. However, the stone artefact industry at Denisova Cave lacks the characteristics of the Sibiryachikha variant found at Chagyrskaya Cave^10^. Accordingly, despite the proximity of the two caves and the presence of an offspring of a Neanderthal mother and a Denisovan father in Denisova Cave some tens of millennia before Chagyrskaya Cave was occupied^25^, we find no evidence of gene flow from Denisovans to the Chagyrskaya Neanderthals in the last 20,000 years before the Chagyrskaya individuals lived (Supplementary Information section 6).

## Inferring social organization

We investigated the community and population size of the Chagyrskaya Neanderthals through time using genomic segments of homozygosity from 8 individuals (those with more than 0.9-fold genomic coverage) (Supplementary Information section 9). Long segments of homozygosity (greater than 10 cM) in an individual imply that their parents shared a very recent common ancestor around ten generations ago and were, therefore, probably part of a small community^5,36^. In addition, the overall proportion of the genome with intermediate length segments of homozygosity (2.5–10 cM) is informative of the size of the population over a slightly longer time frame (around 10–40 generations).

Previous analyses of high-coverage Neanderthal genomes from the Altai mountains revealed that around 16.7% of the genome of Denisova 5 (ref. ^2^) and 19.3% of the genome of Chagyrskaya 8 (ref. ^5^) had intermediate and long segments of homozygosity. One explanation for these patterns is that their parents were second-degree relatives^2^ against a background of unrelated individuals, in which case we would expect most other individuals to have fewer homozygous segments. Alternatively, these data could be due to small local communities^5^, in which case all individuals, except recent immigrants and their descendants, would have extensive segments of homozygosity.

**Fig. 2:**
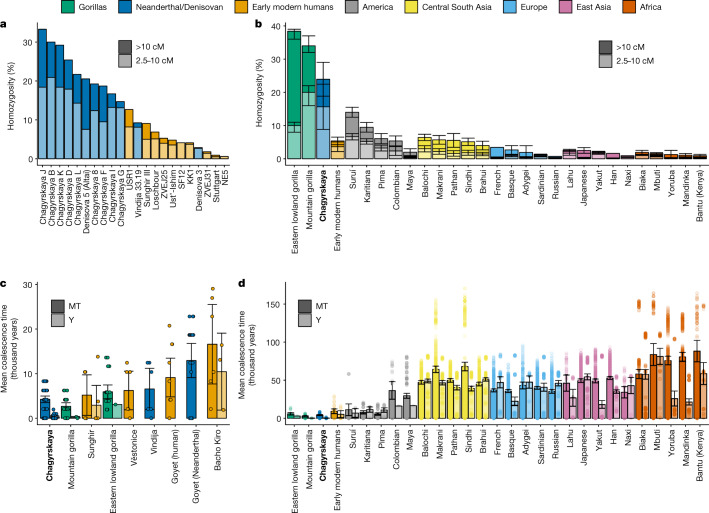
Genomic diversity for Chagyrskaya Neanderthals compared with other hominids. Neanderthal (blue), early modern human (orange) and present-day gorilla (green) populations are coloured the same throughout the figure. Present-day human populations are coloured according to the geographical region (see colour key). **a**, The proportion of the genome that is in homozygous tracts longer than 10 cM (dark) and tracts between 2.5 and 10 cM (light colour) for ancient individuals (early modern humans, Neanderthals and Denisovans). **b**, Average proportion of the genome that is homozygous for Chagyrskaya Neanderthals, early modern humans (grouped together) and present-day human and gorilla populations^37^. Data are mean ± 95% confidence intervals for the estimates of the mean. The sample size is equal to that of the mtDNA sequences listed below. **c**, Mean coalescence time for mtDNA (MT) and Y chromosome (left and right bars of each pair, respectively) for Neanderthal, early modern human and gorilla populations. **d**, Mean coalescence time for early modern humans (grouped together) and present-day human and gorilla populations. **c**,**d**, Data are mean ± 95% confidence intervals and points are all pairwise comparisons. The number of Y chromosome and mtDNA-genomes used in pairwise comparisons for each population is as follows: Neanderthal and Denisovan, Chagyrskaya (MT = 12, Y = 6), Vindija (MT = 4, Y = 0), Goyet  (Neanderthal) (MT = 7, Y = 0); early modern humans, Sunghir (MT = 4, Y = 4), Věstonice (MT = 4, Y = 0), Goyet (MT = 5, Y = 0), Bacho Kiro (MT = 4, Y = 3), which combined is (MT = 17, Y = 7); gorillas, mountain gorilla (MT = 8, Y = 3), eastern lowland gorilla (MT = 7, Y = 2); Americas, Suruí (MT = 9, Y = 4), Karitiana (MT = 13, Y = 5), Pima (MT = 14, Y = 7), Colombian (MT = 8, Y = 2), Mayan (MT = 22, Y = 2); central South Asia, Balochi (MT = 25, Y = 24), Makrani (MT = 26, Y = 20), Pathan (MT = 25, Y = 19), Sindhi (MT = 25, Y = 20), Brahui (MT = 26, Y = 25); Europe, French (MT = 29, Y = 11), Basque (MT = 24, Y = 15), Adygei (MT = 17, Y = 7), Sardinian (MT = 29, Y = 15), Russian (MT = 26, Y = 16); East Asia, Lahu (MT = 9, Y = 7), Japanese (MT = 28, Y = 19), Yakut (MT = 26, Y = 18), Han (MT = 34, Y = 15), Naxi (MT = 9, Y = 6); Africa, Biaka (MT = 23, Y = 22), Mbuti (MT = 14, Y = 10), Yoruba (MT = 23, Y = 11), Mandinka (MT = 23, Y = 14), Bantu (Kenya) (MT = 12, Y = 10).

In all 8 individuals with sufficient coverage, we observed that 1.6–14.9% of the genome had long segments of homozygosity and 9.5–20.5% had intermediate segments of homozygosity (Fig. 2a and Supplementary Table 9.2). We note that both proportions were probably underestimates owing to difficulties in identifying runs of homozygosity at lower coverages (Supplementary Table 9.1). Because we find high amounts of homozygosity in all individuals, we conclude that the local community size of the Chagyrskaya Neanderthals was small. The amount of homozygosity is also similar to the amount found in the genomes of present-day mountain gorillas^37^ (Fig. 2b), an endangered species that lives in small communities of 4–20 individuals^38^, in which it has been observed that matings between second-degree-related individuals are rare^39^.

**Extended Data Fig. 6:**
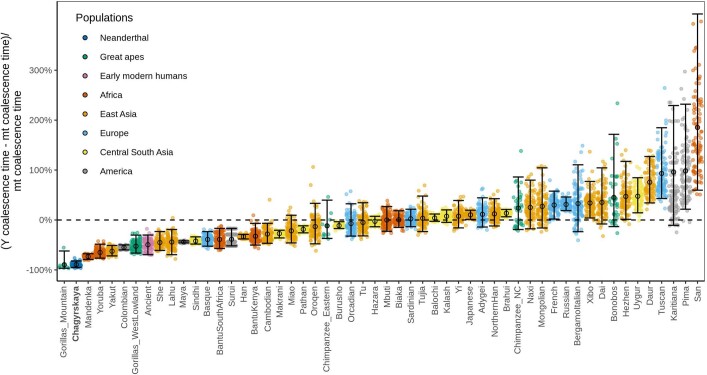
Ratios of mitochondrial DNA to Y-chromosome diversity. Black circles indicate mean estimates for each population and error bars are the corresponding 95% confidence intervals using 100 bootstrap iterations. Negative values denote lower Y-chromosome diversity than mitochondrial (mt) DNA diversity.

To further investigate the social organization of the Chagyrskaya Neanderthals, we contrasted the diversity of the 11 maternally inherited mtDNA sequences with the 6 paternally inherited Y-chromosome sequences. In a randomly mating population without sex-biased processes, the average coalescence time is expected to be the same for both uniparental markers. However, the observed average coalescent time for the Y chromosome (446 years; 95% confidence interval, 113–1,116 years) is significantly lower than that of the mitochondrial genome (4,348 years; 95% confidence interval, 2,043–6,196 years; Wilcoxon rank-sum test, *P* = 4.1 × 10^–5^). In a comparison with 47 modern human populations and 10 great ape subspecies, the Chagyrskaya Neanderthals have among the lowest ratios of Y-chromosome-to-mtDNA coalescence time, with only mountain gorillas having a more extreme ratio (Extended Data Fig. 6). We caution that similar ratios between apes and Neanderthals do not necessarily mean that the communities have the same social organization, as there are multiple caveats. First, the great ape data are very heterogeneous—for example, although some great apes were born in the wild, others were born in captivity (that is, in artificial communities) and often the sample sizes were very small (Supplementary Table 8.1). Second, several different scenarios may lead to similar Y-chromosome-to-mtDNA ratios. These include: differences in male and female generation times, a skewed offspring distribution among males (that is, a subset of males father the majority of the children) and female-biased migration. To test the relative importance of these processes, we simulated a large number of combinations of these scenarios, fitting the diversity of Y chromosomes and mtDNA and their ratio to the observed data (Supplementary Information section 8). We approximated the likelihood of each scenario using simulations as the proportion of simulated datasets that are within the 95% confidence intervals of the observed data. We then used the Akaike information criterion (AIC) to rank different scenarios (Supplementary Table 8.5).

The best-fitting scenarios (AIC = 6.2) assumed a community size of 20 individuals, with 60–100% of the females being migrants from another community (Supplementary Table 8.4). However, the shared heteroplasmy between Chagyrskaya C and Chagyrskaya D suggests that at least some females remained with the group they were born in. Scenarios that include only skewed offspring distributions explain the data less well (AIC = 7.4) and require large community sizes of 300 individuals. Scenarios with both skewed offspring distributions and female migrations does not improve the fit (AIC = 8.5) obtained by assuming migration-bias alone. Scenarios that include only differences in generation time fit the data poorly (AIC = 8.5) and require parameter settings that seem unrealistic (for example, females would need to be on average twice as old as males, Supplementary Table 8.4). Previous estimates of Neanderthal community sizes range from 3 to 60 individuals^5,16,17,19^ and, in this range, the best fitting scenarios include female migration (Supplementary Fig. 8.4). This result suggests that female-biased migration was a major factor in the social organization of the Chagyrskaya Neanderthal community.

## Conclusion

We present genetic data from 13 Neanderthals, making this one of the largest genetic studies of a Neanderthal population. For the first time, to our knowledge, we document familial relationships between Neanderthals, including a father-and-daughter pair.

The high degree of homozygosity in all individuals is similar to what is seen in mountain gorillas^40^, consistent with Neanderthals in the Altai living in small communities. Furthermore, based on the shorter average coalescent time for the Y chromosomes than for the mtDNA and shared mtDNA variants between Chagyrskaya and Okladnikov individuals, we suggest that these small Neanderthal communities were predominantly linked by female migration.

Our findings raise questions as to whether the characteristics of the Altai communities are related to their isolated geographical location at the easternmost extremity of the known range of Neanderthals (especially because the population size at Vindija Cave was probably larger^5^), or whether they are characteristic of Neanderthal communities more broadly.

Future studies should, therefore, when possible, aim to sample multiple individuals from additional Neanderthal communities in other parts of Eurasia to shed further light on the social organization of our closest evolutionary relatives.

## Methods

No statistical methods were used to predetermine sample size. The experiments were not randomized and the investigators were not blinded to allocation during experiments and outcome assessment. A detailed description of all analyses carried out in this study is included in the Supplementary Information. Permission to work on the archaeological specimens was granted based on a written agreement of scientific cooperation signed in 2018 by the Federal State Budgetary Institution of Science–Institute of Archaeology and Ethnography, Siberian Branch of the Russian Academy of Sciences and the Max Planck Institute for Evolutionary Anthropology.

### Reporting summary

Further information on research design is available in the Nature Research Reporting Summary linked to this article.

## Online content

Any methods, additional references, Nature Research reporting summaries, source data, extended data, supplementary information, acknowledgements, peer review information; details of author contributions and competing interests; and statements of data and code availability are available at 10.1038/s41586-022-05283-y.

### Supplementary information

Supplementary Information A detailed description of all of the analyses carried out in this study is provided in Supplementary Information sections 1–10, Supplementary Figs. 1–42, Supplementary Tables 1–44 and Supplementary References.

Reporting Summary

Supplementary Data 1 All information on the sequencing of libraries, including the treatment and complexity of the libraries and contamination estimates.

Peer Review File

## Figures and Tables

**Extended Data Table 1:**
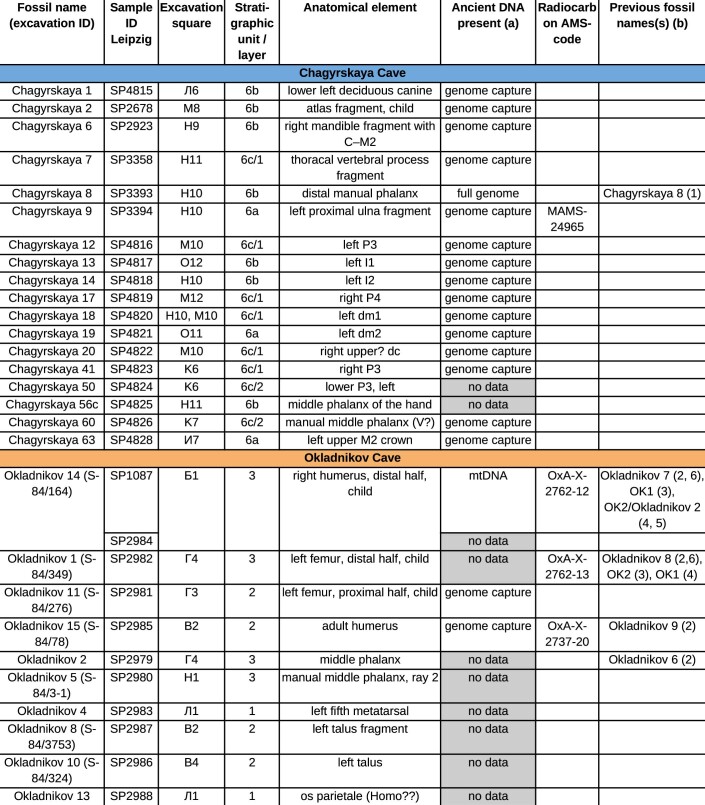
Neanderthal remains from Chagyrskaya and Okladnikov Caves included in this study for DNA analysis or ^14^C dating

All remains from Chagyrskaya Cave were recovered from lithoseries II, and all those from Okladnikov Cave were recovered from the Shelter. (**a**) This study, except for *Chagyrskaya 8*^*5*^ and Okladnikov sample SP1087 (mtDNA only^41^); ’genome capture’ indicates that mitochondrial, Y-chromosome and nuclear capture has been performed, and ‘no data’ indicates that no ancient DNA was detected. (**b**) Source references: 1,^5^; 2,^54^; 3,^55^; 4,^56^; 5,^41^; 6^25^.

## Data Availability

Raw data for each library are available in the European Nucleotide Archive under accession number PRJEB55327. Mapped BAM files for all specimens and individuals, VCF files, consensus FASTA mtDNA sequences and a multiple alignment of all mtDNA can be downloaded from http://ftp.eva.mpg.de/neandertal/ChagyrskayaOkladnikov/.
