# Supplementary material for: Genetic insights into the social organization of Neanderthals
Source: Nature. 2022 Oct 19;610(7932):519–25. doi: 10.1038/s41586-022-05283-y (PMC9581778; doi:10.1038/s41586-022-05283-y)
Supplement: Supplementary file 1 — A detailed description of all of the analyses carried out in this study is provided in Supplementary Information sections 1–10, Supplementary Figs. 1–42, Supplementary Tables 1–44 and Supplementary References. [file 41586_2022_5283_MOESM1_ESM.pdf]

nature portfolio

https://doi.org/10.1038/s41586-022-05283-y

Supplementary information

Genetic insights into the social organization of Neanderthals

In the format provided by the authors and unedited

Table of contents

SI 1 Stratigraphy, chronology and archaeology **3**
Chagyrskaya cave 3
Okladnikov Cave 8
Sibiryachikha Neanderthals 12
Neanderthal remains 14
Age estimates for remains 14
Chagyrskaya remains 15
Age estimation of associated Chagyrskaya remains 20
Okladnikov Cave remains 24

SI 2 DNA extraction, lib prep, sequencing **26**
DNA Extraction and library preparation 26
Initial shotgun sequencing 26
Additional shotgun sequencing 27

SI 3 Mitochondrial (mt) DNA captures and sequencing **28**
Captured libraries 28
Modern human contamination estimates 28
Reconstruction of the mitochondrial genomes 29
Curated data set 31
Heteroplasmies 32
Phylogenetic relationship 38
TMRCA for mitochondrial sequences 47

SI 4 Y chromosome DNA capture and sequencing **48**
Array design 48
Coding genes and transcription factors on the Y chromosome array 48
Additional Y chromosomes used 49
Captured libraries 49
Modern human contamination estimates 49
Testing contamination estimates 50
Chagyrskaya and Okladnikov contamination estimates 53
Curated data set 54
Detailed coverage across the Y chromosome 55
Results on simulated data 55
Results for Neanderthal and Denisovan libraries 56
Variant calling 63
Phylogenetic analysis 67
Placing low coverage samples on phylogenetic tree 69
Variant annotation 70
Dating of Y chromosomes 71

SI 5 Nuclear DNA capture and sequencing **72**
Array design 72
Samples 72
Filtering 72
Ascertainments 73
Probe Design 74
Captured libraries 74
Capture efficiency and bias 75
Probes found both in archaic component and African component 77
Probes that overlap other probes 79
Modern human contamination estimates 82
Contamination from non-human sources 82
Curated data set 84
Sex determination 85

SI 6 Relationship to other archaic individuals **86**
Chagyrskaya individuals are closer to Chagyrskaya 8 86
Data processing 86
Comparison between High-Coverage Archaics 86
Chagyrskaya 12 is the same individual as Chagyrskaya 08 88
Denisovan introgression 89

SI 7 Relatedness analysis **92**
Relationship between Chagyrskaya and Okladnikov remains 99
Relationship between contemporaneous and non-contemporaneous individuals 100

SI 8 Mitochondrial and Y chromosome diversity **101**
Data sets used 101
Mitochondrial and Y chromosome diversity 105
Comparing absolute diversity levels in Chagyrskaya to other populations 106
Comparing ratio of mt to Y chromosome diversity 107
Causes for difference in Y chromosome and mt coalescence time 108
Simulation parameters 108
Simulation results 110

SI 9 Diversity and runs of homozygosity **113**
Runs of homozygosity 113

SI 10 Diversity ratio of X chromosome and autosomes **117**

References **120**

SI 1 Stratigraphy, chronology and archaeology

Chagyrskaya cave

Chagyrskaya Cave (51°26′34.6″ N, 83°09′18.0″ E) is located on the left bank of the Charysh River in the Tigirek Range in the northwestern foothills of the Altai Mountains (**SI Figure 1.1 A,B**). The cave faces north and is situated 19 m above the river at an elevation of 353 m above sea level. The cave consists of two chambers with a total area of 130 m^2. Investigations in the cave started in 2007, since when more than 45 m^2 have been excavated by two teams (**SI Figure 1.2**).

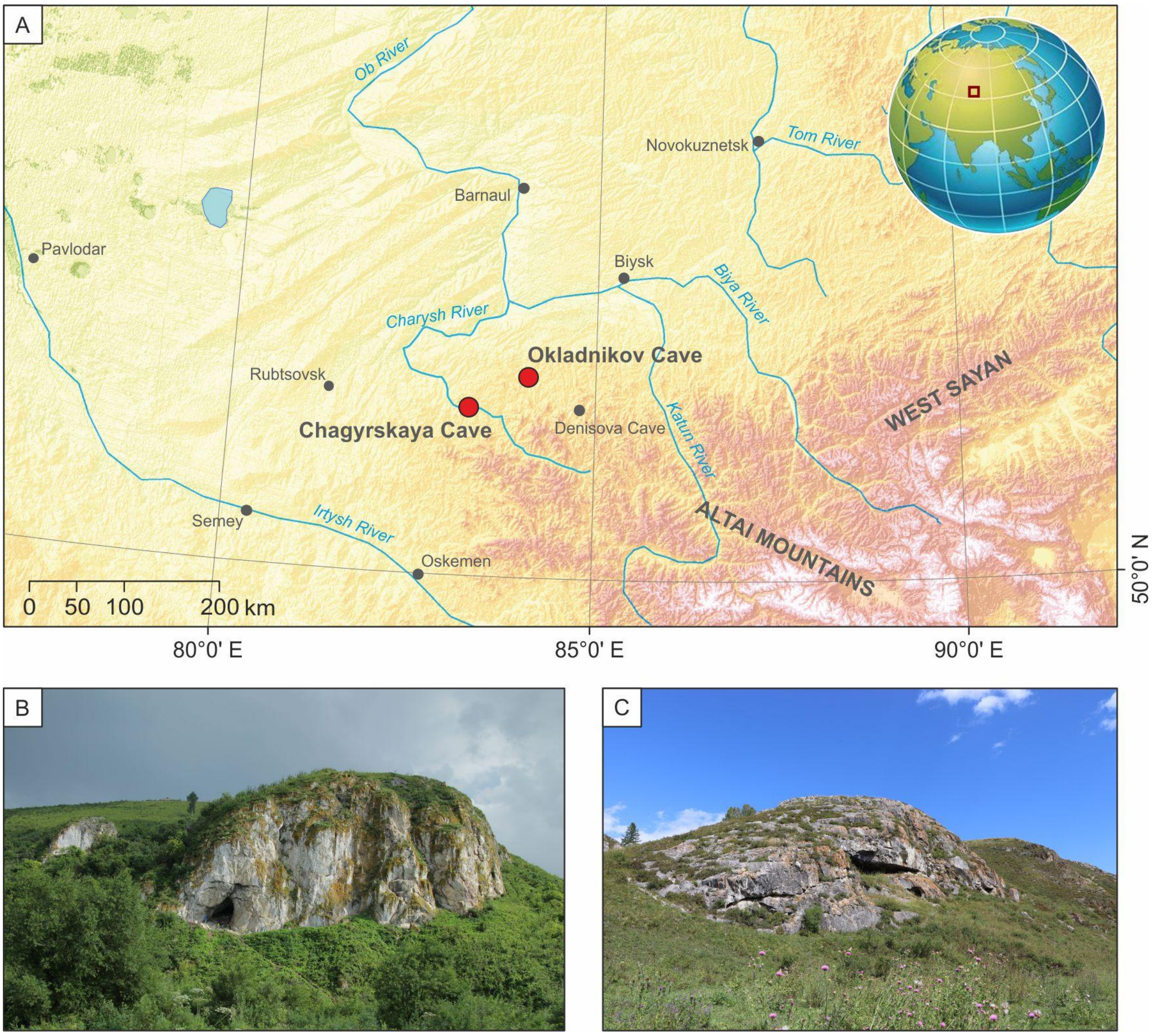

SI Figure 1.1. Location map of Chagyrskaya and Okladnikov Caves in the Altai region of southern Siberia (A). Views of the north-facing entrance to Chagyrskaya Cave (B) and the south-facing entrance to Okladnikov Cave (C).

In addition to yielding the largest collection of Neanderthal remains in North Asia, the archaeological assemblages at Chagyrskaya Cave are the easternmost examples known of the Micoquian/*Keilmessergruppen* (KMG) technocomplex, a unique tradition of stone artefact

production associated with Neanderthals[1]. Micoquian artefacts have been found at sites in Central and Eastern Europe, the Caucasus and the Crimea, and connections with these regions are also evident from sequencing of Neanderthal DNA from Chagyrskaya Cave. The high-coverage genome of a Neanderthal phalanx (*Chagyrskaya 8*) and nuclear DNA recovered from the cave sediments show that the Chagyrskaya Neanderthals have closer genetic affinities with late European Neanderthals than they do with Neanderthal remains retrieved from the Middle Palaeolithic layers of another site in the Altai—Denisova Cave—located ~100 km east of Chagyrskaya Cave[2]

The stratigraphy, sedimentology and micromorphology of the deposits in Chagyrskaya Cave are described in detail elsewhere[1,3,4].

The stratigraphic sequence is up to 3.5 m thick and consists of Holocene and Pleistocene deposits that are divided into layers (e.g., layer 6), subunits (e.g., subunit 6c) and sublayers (e.g., sublayer 6c/1) based on lithological differences and erosional features. These various stratigraphic units are grouped into three lithoseries[1], with Neanderthal remains recovered from lithoseries II (**Extended Table 1**, **SI Figure 1.2** and **SI Figure 1.3**).

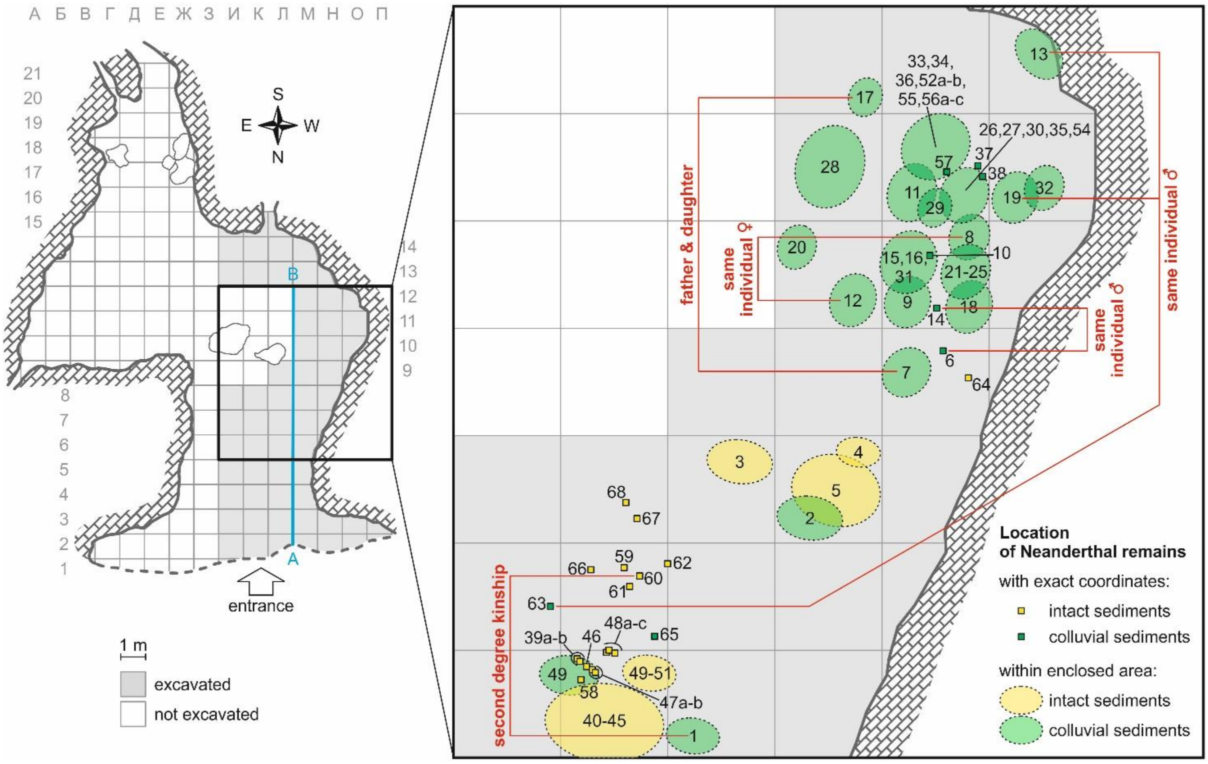

SI Figure 1.2. Spatial distribution of Neanderthal remains in Chagyrskaya Cave. The plan of the cave interior shows the excavated area in grey, and the blue line (transect A–B) marks the position of the stratigraphic profile shown in **SI Figure 1.3**. The coloured squares and ellipses denote Neanderthal remains located with exact coordinates or within the circumscribed areas, respectively, and are annotated with the corresponding remain number(s).

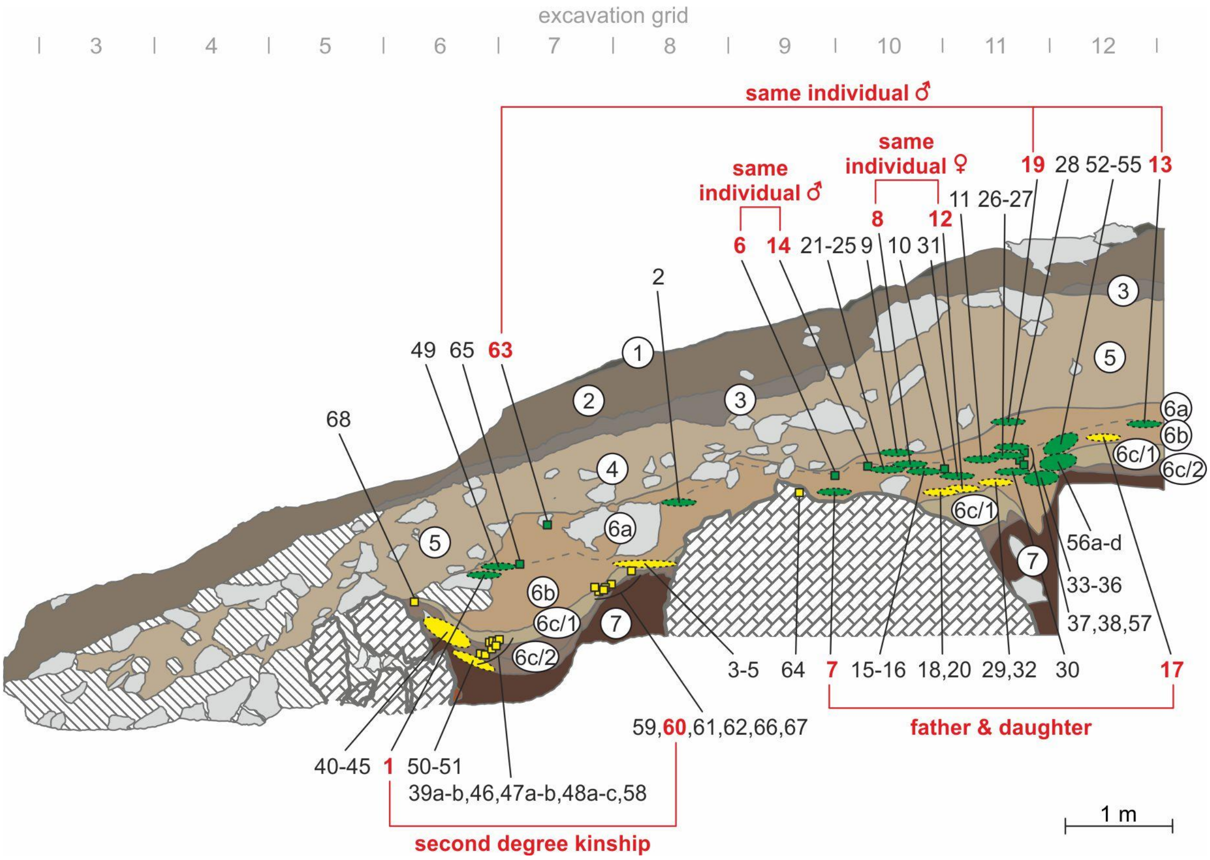

SI Figure 1.3. Stratigraphic profile of Chagyrskaya Cave along transect A–B in **SI Figure 1.2**. Locations of Neanderthal remains are projected orthogonally onto this profile, so each remain is not necessarily shown in the stratigraphic unit from which it was recovered. Symbols are the same as in **SI Figure 1.2**.

Lithoseries II is divided here into lower and upper portions, which we refer to as subseries IIa and IIb, respectively (**SI Table 1.1**). Subseries IIa is comprised of intact sediments, with Middle Palaeolithic artefacts and Neanderthal remains preserved *in situ*.

Lithoseries	Stratigraphic unit	Depositional environment
I	Layers 8 and 7 (subunits 7a, 7b and 7c)	Fluvial sediments
II, subseries IIa	Layer 6 (subunits 6c and 6d, and sublayers 6c/1 and 6c/2)	In situ cave sediments (aeolian deposition and rock fall debris, with anthropogenic and zoogenic components)
II, subseries IIb	Layers 6 (subunits 6a and 6b), 5 (subunits 5a and 5b) and 4	Redeposited colluvial sediments
III	Layers 3, 2 and 1	Redeposited colluvial sediments with *in situ* anthropogenic component

SI Table 1.1. Stratigraphic units in Chagyrskaya Cave and reconstructed modes of deposition (based on[1]).

Subseries IIb has a similar lithological composition but was redeposited by colluvial processes, so the artefacts and Neanderthal remains are in secondary context. The colluvial material likely originated from subseries IIa and lithoseries I sediments that were present

somewhere deeper in the cave. These sediments were redeposited and possibly mixed (as indicated by their micromorphological features) over a short time interval (a few millennia at most), as indicated by the narrow age range (49–59 ka at 95% probability) determined from optical dating of sediments from layers 6 and 5[1]. We also identify two remains that show a second degree relationship - one in the subseries IIa and another in subseries IIb.
These remains are *Chagyrskaya 60*, found in the intact sediments (subseries IIa), and *Chagyrskaya 1*, found in the colluvial sediments (subseries IIb) (**SI Figure 1.2** and **SI Figure 1.3**).

Independent support for the rapid accumulation of these layers is provided by the results of attributive analysis of the artefact assemblage[1], which indicates that subseries IIa and IIb contain similar lithic and bone assemblages in terms of composition and techno-typological characteristics. Furthermore, the Neanderthal DNA sequences obtained from the cave sediments are remarkably homogeneous and group with the DNA sequences obtained for *Chagyrskaya 8* and *Okladnikov S-84/349* (a Neanderthal from Okladnikov Cave), which is consistent with a short-lived occupation of Chagyrskaya Cave by Neanderthals[5].

Radiocarbon (^{14}C) and optical ages have been reported previously[1] for 20 bison remains and 23 sediment samples, respectively, from layers 5 and 6 (lithoseries II). Fourteen of the ^{14}C samples yielded infinite ages of >49 ka BP, consistent with a weighted mean optical age of 54.0 ± 2.5 ka (total uncertainty at 1σ) for these layers (**SI Table 1.2**).

**Lithoseries**	**Layer / subunit**	**Number of samples**	**Arithmetic mean age (ka)**	**Weighted mean age (ka)**
Subseries IIb	5	6	53.9 ± 2.8	53.7 ± 2.7
	6a	4	50.8 ± 2.5	50.6 ± 2.4
	6b	2	52.1 ± 2.5	52.1 ± 2.9
Subseries IIa	6c	9	55.1 ± 2.6	54.9 ± 2.5
	6d	2	61.3 ± 3.4	61.6 ± 3.8
**Both subseries**	5 + 6	23	54.3 ± 2.5	54.0 ± 2.5

SI Table 1.2. Optical ages for sediment samples from layers 5 and 6 at Chagyrskaya Cave. Total uncertainties on arithmetic and weighted mean ages are at 1σ. Weighted mean ages were calculated using the central age model[6]. The final row indicates the arithmetic and weighted mean ages for all samples from both subseries.

Here we report three additional ^{14}C ages for individual pieces of charcoal and one additional ^{14}C age on a human bone fragment. The charcoals were obtained from a hearth preserved in sublayer 6c/2. At the Oxford Radiocarbon Accelerator Unit (ORAU), samples were prepared using an oxidation protocol (modified ABOx-SC) to or remove or reduce contamination from younger carbon, and were then measured by accelerator mass spectrometry.

The human bone (from the remain *Chagyrskaya 9*) comes from sublayer 6a. The samples were pretreated at the Department of Human Evolution of Max Planck Institute for Evolutionary Anthropology (MPI-EVA), Leipzig, Germany, using the method described in[7], and[8]. The sample was sent to Curt Engelhorn Centre for Archaeometry (CEZA), Mannheim, Germany (Lab Code MAMS), where it was graphitized and dated[9].

**AMS- code**	**Sample**	**^{14}C age (yr**	**fM**	**Used (mg)**	**Yield (mg)**	**%C**	**δ13C**

		BP)					(‰)
OxA-37966	1. Chag 3442 L6v2 h-6	>51,100	0.0008 ± 0.00046	103.02	7.47	82.8	–24.9
OxA-37967	2. Chag 3517 L6v2 h-7	>53,800	0.00037 ± 0.00043	71.78	8.81	84.2	–25.3
OxA-38004	2. Chag 3517 L6v2 h-7	>53,400	0.00041 ± 0.00045	79.04	10.52	83.6	–25.9
MAMS-24965	3. Chag 3394 L6a	>50,000	0,0007 ± 0,0005	499.5	38.5	34.4	-15.5

SI Table 1.3. New ^{14}C ages for charcoal pieces from sublayer 6c/2 and the human bone date (MAMS-24965) from unit 6a at Chagyrskaya Cave OxA-37967 and -38004 are duplicate ages. 'fM' denotes the fraction modern value, after correction for background carbon values subtracted for pretreatment chemistry. 'Used' denotes the weight of material used (in mg), 'Yield' is the weight of extracted carbon (in mg), '%C' represents the carbon present and 'δ^{13}C' is the stable isotope ratio of carbon expressed in delta notation (in per mille, measured with a precision of ± 0.2‰) relative to the VPDB (Vienna Pee Dee Belemnite) standard.

The resulting ages of >51 ka BP (**SI Table 1.3**) also align with the optical ages, indicating that layers 5 and 6 accumulated sometime between 49 and 59 ka ago (95.4% confidence interval), probably over a few millennia or less. The time span of deposition cannot be resolved with higher precision because of the size of the age uncertainties. The optical ages are consistent also with the timing inferred from pollen, faunal and sedimentological data[1], but they are younger than the DNA-based age estimate of ~80 ka for *Chagyrskaya 8*[2]; potential causes of this mismatch are discussed in those two articles. The Y-chromosome data obtained in the present study give a genetic age for the Chagyrskaya Neanderthals that is more similar to the sediment chronology (**SI 4 - Dating of Y chromosomes**): ~63.9 ka (95.4% confidence interval: 48.5–82.9 ka), assuming a split time of ~100 ka for the *Mezmaiskaya 2*, *Spy 94a* and Chagyrskaya Y chromosomes[10].

Until this issue is resolved, we conservatively adopt an age range of 49–92 ka for the Neanderthal remains in Chagyrskaya Cave, which encompasses the 95.4% confidence intervals for the various age estimates. Neanderthals briefly occupied the cave at some point during this time interval[4] have suggested that given the limited area of living space in the cave, it may have been used mainly as a camp for the selective hunting and consumption of bison and horses, with a preference for non-adult individuals and females. The small number of individuals is consistent with genetic analyses indicating that the Neanderthal community in the region was likely small ([2]; **SI 8 - Mitochondrial and Y chromosome diversity** and **SI 9 - Diversity** and runs of homozygosity).

Many of the animal bones show signs of human modification[11], with cutting, scraping or other impact marks identified on 31% of the faunal remains retrieved from sublayer 6c/2[4]. In addition to the remains of adult Neanderthals, teeth and skeletal remains of juvenile and sub-adult Neanderthals have also been recovered from the cave deposits (**Extended Table 1**, **SI Figure 1.2** and **SI Figure 1.3**), indicating that children and teenagers were also present at the site.

Okladnikov Cave

Okladnikov Cave (51°43′53.59″ N, 84°02′22.73″ E) is located ~70 km northeast of Chagyrskaya Cave, on the left bank of the Sibiryachikha River in the Altai foothills. The cave faces south and is situated 14 m above the river at an elevation of 340 m above sea level (**SI Figure 1.1 A,C**). The cave complex consists of several chambers and galleries (the largest being ~33 m2 in area), as well as an overhanging ‘Shelter’ at the cave mouth that leads into a 3.3 m-long passage called the ‘Grotto’ (**SI Figure 1.4 A**). Archaeological excavations conducted between 1984 and 1986 resulted in the complete removal of the unconsolidated sediments.

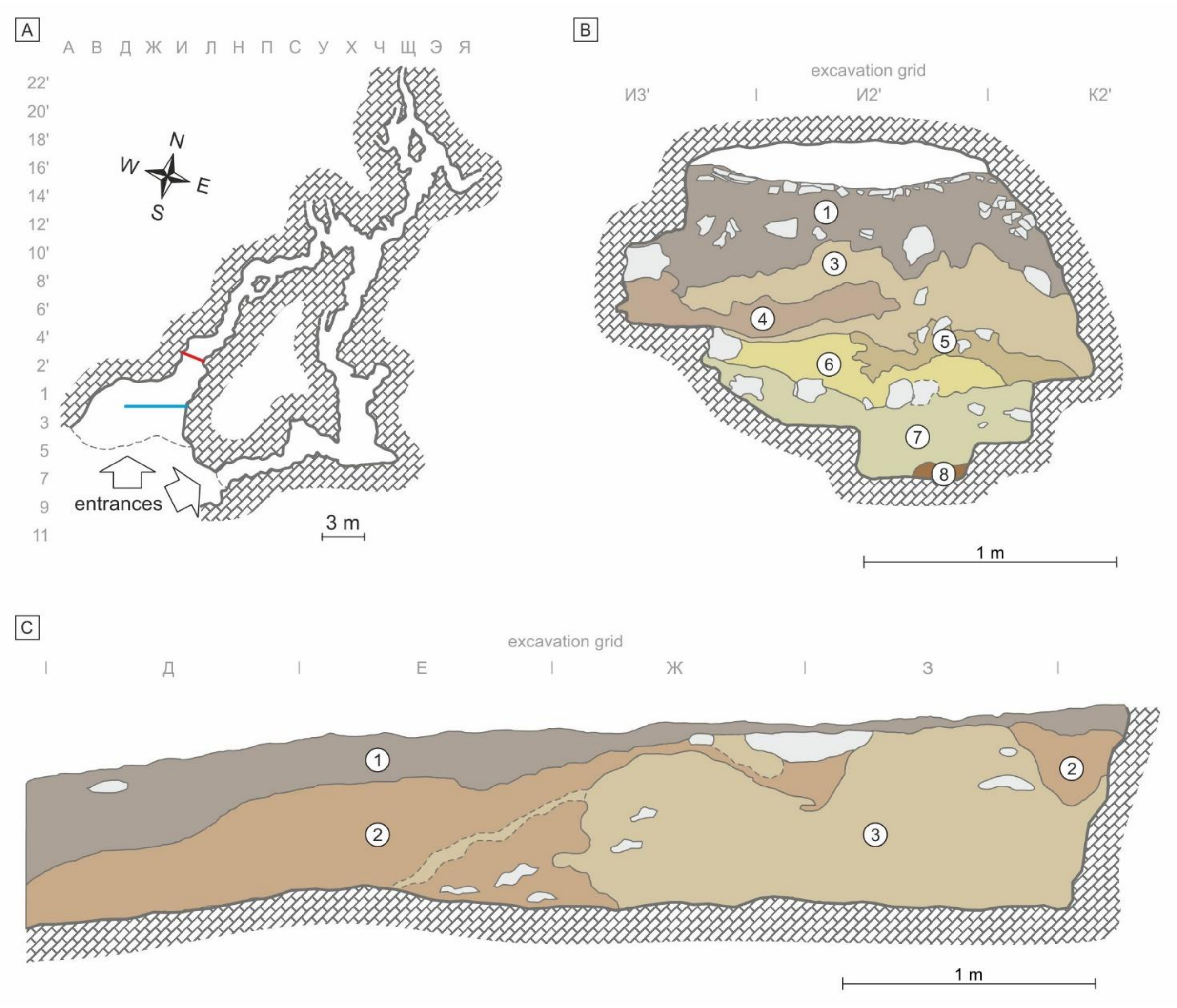

SI Figure 1.4. Plan of the interior of Okladnikov Cave (A). The excavation grid is labelled using alternate square numbers and letters, and the red and blue lines mark the positions of the stratigraphic profiles in the Grotto (B) and the Shelter (C), respectively. Lithological layers are numbered 1–8 and areas consisting of large rocky debris are shown in grey.

The stratigraphic sequence of Okladnikov Cave includes seven lithological layers in the Grotto (layers 1 and 3–8; **SI Figure 1.4 B**) and three lithological layers in the Shelter (layers

1–3; **SI Figure 1.4 C**). Full stratigraphic details are given in[12] and[13], with summary accounts in[14] and[15].

Layer 1 is a dark grey loam containing angular limestone fragments of various sizes, with an unconformable (erosional) contact with layer 2; **layer 2** is a yellowish brown loam with isolated limestone fragments; **layer 3** is a greyish brown loam with vertically-oriented, angular limestone fragments; **layers 4 and 5** are pale yellow and dark brown sandy loams, respectively, both of limited spatial extent and with rounded fragments of limestone in layer 4; **layers 6 and 7** are dark brown and reddish loams, respectively, with small limestone fragments in the middle part of layer 6 and pieces of green shale in the lower part of layer 7; and **layer 8** is a red clay containing small pieces of quartz.

Layer 1 is Holocene in age, layers 2–7 are Pleistocene and layer 8 is pre-Pleistocene. Archaeological and palaeontological remains are most abundant in layers 2 and 3, and the Neanderthal remains analyzed in the present study were recovered from these layers and near each other in the Shelter.

Both layers consist of redeposited colluvial sediments washed through the Grotto, aeolian materials deposited directly in the Shelter, limestone debris from the cave roof and walls, and zoogenic and anthropogenic components. The two layers are intercalated in the Shelter (**SI Figure 1.4 B**), due in part to post-depositional disturbance by cave fauna (e.g., hyena and rodent activities). The sequenced and/or directly dated Neanderthal remains from layer 2 (*Okladnikov 11* and *15*) and layer 3 (*Okladnikov 1* and *14*) (Extended Data Table 1) might therefore have similar ages or, if layer 3 was deposited much earlier than layer 2, represent a mixed-age assemblage. We consider it more likely that these individuals are broadly contemporaneous, because (a) the hydroxyproline-based ^{14}C ages reported here for *Okladnikov 1, 11* and *15* are all >40 ka (see below), and (b) the Neanderthals who occupied Chagyrskaya Cave (for a short period sometime between 59 and 51 ka) and are genetically similar to *Okladnikov 11, 14* and *15* (Figure 1 C, where they are labeled *Okladnikov A*, *2* and *B*, respectively), which implies that the two sites were inhabited at around the same time (i.e., within a few millennia of each other).

The stone artefact assemblages from layers 2–7 are characterized by a low proportion of cores (radial and Levallois cores being the most numerous) and a high proportion of tools (up to 25%). Tool kits are dominated by simple, trapezoidal and leaf-shaped convergent scrapers; plano-convex Keilmesser and plano-convex leaf-shape and crescent scrapers have also been found (**SI Figure 1.6**). The most abundant faunal remains are of horse, ibex/argali, wooly rhinoceros, bison and red deer, suggesting use of the site as a hunting camp[16]. The Okladnikov site has been dated previously using ^{14}C and U-series methods[13]; [17]: **Extended Table 1**). Seven ^{14}C ages have been reported for faunal remains from layers 1–3, in addition to four ^{14}C ages for two Neanderthal remains from layers 2 and 3, and four U-series ages on two animal bones from layers 3 and 7. All previous ^{14}C determinations are highly likely to be problematic, due to the incomplete removal of contamination and limitations in the pretreatment chemistry applied. For example, *Okladnikov S-84/164* (listed by [17] as *OK1*), the distal half of a juvenile right humerus from layer 3, was dated by three different laboratories using different chemical pretreatments, yielding ^{14}C ages of between 30 and 38 ka BP. This wide spread in ages, which are also surprisingly young, suggests that the pretreated samples of bulk collagen may be contaminated by younger carbon to differing extents. For this reason, we decided to re-date the Neanderthal bones from the site at

ORAU using a compound-specific approach, focusing on the amino acid hydroxyproline. This is the most robust method to remove contaminants from bone[18,19].

We obtained samples from three of the Neanderthal bone remains (now curated at the Institute of Archaeology and Ethnography, Siberian Branch of the Russian Academy of Sciences, Novosibirsk) in June 2017 (**SI Table 1.4** and **SI Figure 1.5**).

**OxA-X code**	**remain excavation ID and element**	**^{14}C age (yr BP)**	**fM**	**Used (mg)**	**Yield (mg)**	**%C**	**δ13C (‰)**	**C:N ratio**
2762-12	S-84/164 juvenile right humerus	>44,100	–0.00057 ± 0.00234	1451	23.04	40.7	–23.5	5.1
2737-20	S-84/78 adult humerus	>40,100	0.00311 ± 0.00185	664	23.16	50.2	–24.0	5
2762-13	S-84/349 juvenile left femur	>39,500	0.00325 ± 0.00203	1307	21.74	40.4	–26.4	5.1

SI Table 1.4. New hydroxyproline ^{14}C ages for Neanderthal remains from Okladnikov Cave. OxA-X is ORAU's prefix for samples prepared using non-routine chemistry. 'fM' is defined as in **SI Table 1.3**, 'Used' denotes the weight of drilled bone used (in mg), 'Yield' is the weight of extracted collagen (in mg), '%C' represents the carbon present in the combusted amino acid hydroxyproline, 'δ^{13}C' is the corresponding stable isotope ratio of carbon expressed in delta notation (in per mille, measured with a precision of ± 0.2‰) relative to the VPDB (Vienna Pee Dee Belemnite) standard, and 'C:N' is the carbon-to-nitrogen atomic ratio (for hydroxyproline, ratios of 4.9–5.1 are considered acceptable).

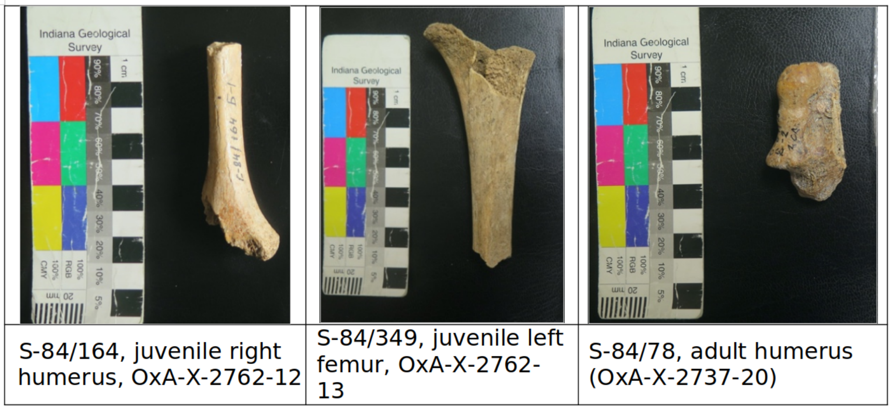

S-84/164, juvenile right humerus, OxA-X-2762-12	S-84/349, juvenile left femur, OxA-X-2762-13	S-84/78, adult humerus (OxA-X-2737-20)

SI Figure 1.5. Directly ^{14}C-dated Neanderthal remains from Okladnikov Cave (photographs T. Higham).

[20] has described the morphology of the bones. We took bone powder samples using an NSK drill kit with a tungsten carbide bit, and extracted collagen using a non-routine method consisting of decalcification using dilute HCl acid, followed by gelatinization and lyophilization. We then hydrolysed the collagen and separated the underivatized amino acids using preparative liquid chromatography (Prep-LC), employing the method described by[19] to collect the amino acid hydroxyproline (HYP). This was then combusted using an EA-IRMS system (Carlo Erba EA1108/Europa Geo 20/20) operating in continuous-flow mode, from which we obtained the C/N atomic ratios and other analytical data (**SI Table 1.4**). Finally, we graphitized the HYP fraction and measured it on the HVEE accelerator mass spectrometer at ORAU.

The results of our single amino acid analyses show that, where we have previous results for comparison, the new determinations are significantly older and, in fact, indistinguishable from the laboratory background for the HYP method (**SI Table 1.4**). *Okladnikov S-84/164* had been dated previously to between 30 and 38 ka BP, but our compound-specific determination indicates that it is >44,100 yr BP. Similarly, a fragment of an adult Neanderthal humerus (*Okladnikov S-84/78*), dated previously by the Leibniz Laboratory for Radiometric Dating and Stable Isotope research (KIA-27010) to 24,260 ± 180 yr BP[17], yielded a compound-specific age of >40,100 BP. These new ages lend further support to suggestions that residual contaminants may have significantly affected the bulk collagen ages obtained previously for the hominin remains—and presumably also the faunal remains—at Okladnikov Cave. Together with the new compound-specific age of >39,500 yr BP for *Okladnikov S-84/349*, the distal half of a juvenile Neanderthal femur from layer 3, the three ^{14}C determinations obtained on the HYP fractions are all minimum ages, indicating that the true age of the Neanderthal remains at the site is at least 44 ka cal. BP.

This minimum age for the directly-dated Neanderthal remains is also consistent with the U-series ages of >39 and >44 ka obtained for two animal bones from layers 3 and 7, respectively ([21]; [13]. Sample Okl-2 (layer 3) was analyzed as a bulk sample of the entire bone and as ‘scrapes’ from the outer surface of the bone, yielding ages of 10,287 +406/–404 yr and 38,725 +1,435/–1,419 yr, respectively. The large difference in age was attributed to uranium leaching, with the older age considered the more reliable but, nonetheless, still a minimum estimate[21]. Sample MB-5 (layer 7) was dated using the two uranium decay chains (238U and 235U) as a test of reproducibility, resulting in 234U/230Th and 235U/231Pa ages of 44,800 ± 4,400 yr, and 44,600 ± 3,300 yr, respectively. Although these ages are concordant, they should both be treated as minimum estimates[21], as any uranium migration will be common to both systems and both daughter/parent ratios will change in the same direction. Bones acquire uranium only after deposition and subsequently act as geochemically open systems, often with a complex (and unknown) history of uranium migration, so U-series ages for faunal remains are now commonly interpreted as minimum estimates[22]. Accordingly, the U-series and compound-specific ^{14}C chronologies for Okladnikov Cave indicate that the Neanderthal and faunal remains in layers 2 and below are older than 44 ka and, therefore, potentially contemporaneous with the Neanderthal remains recovered from layers 5 and 6 at Chagyrskaya Cave.

Sibiryachikha Neanderthals

The stone artefact assemblages at Chagyrskaya and Okladnikov Caves share many distinctive attributes and, together with a small assemblage from a newly discovered site, Upper Sibiryachikha Cave[23], are currently the only sites known to contain the 'Sibiryachikha variant' of the Altai Middle Palaeolithic[24]. Technological similarities include the predominance of radial flake cores and plano-convex bifaces (including Keilmesser types Bocksteinmesser and Klausennischemesser), while typological similarities include the predominance of simple scrapers and trapezoidal and leaf-shaped convergent scrapers of identical appearance in the two assemblages (**SI Figure 1.6**).

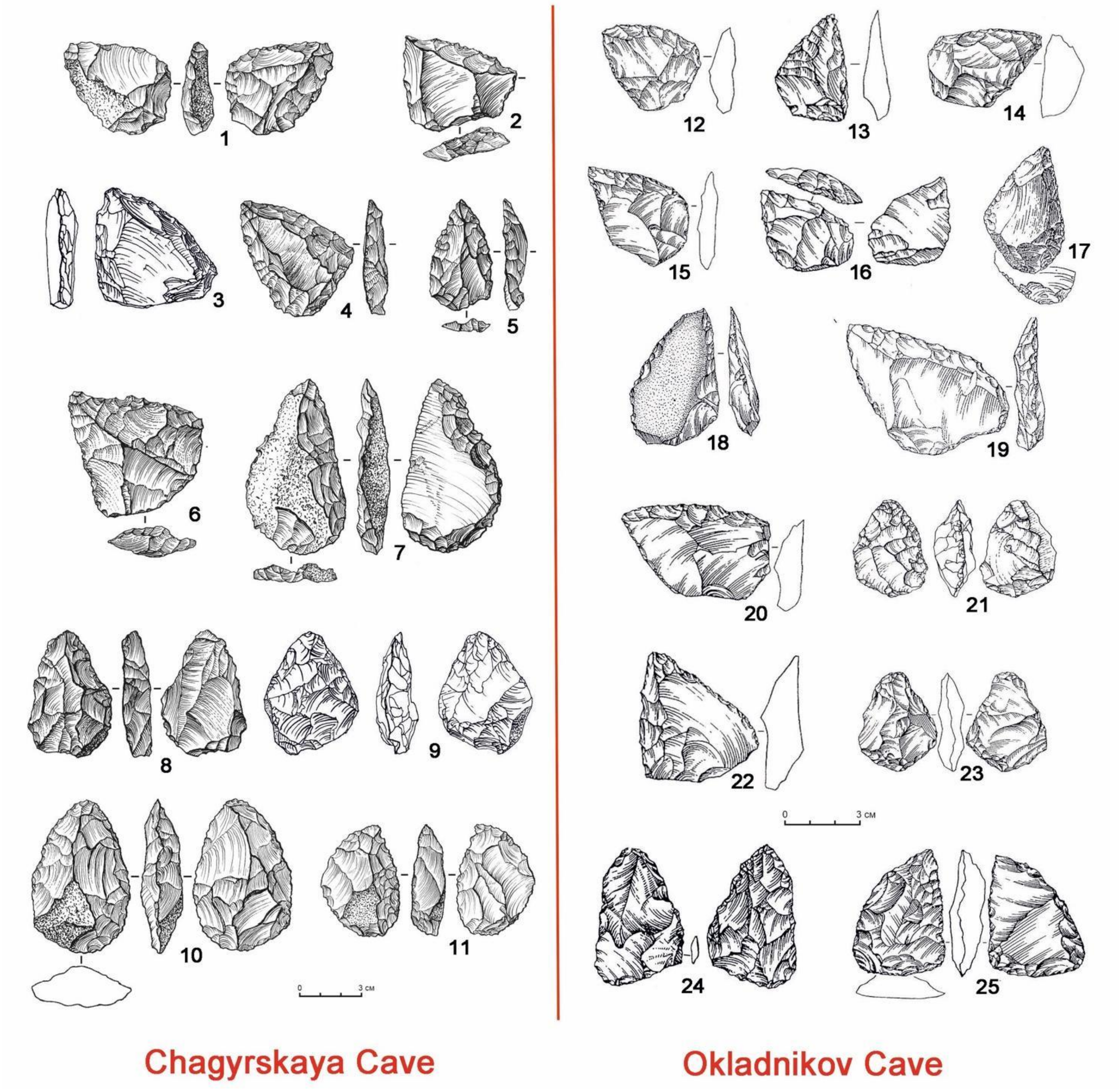

SI Figure 1.6. Comparison of lithic tools from Chagyrskaya and Okladnikov Caves: plano-convex Klausennischemesser (1, 21), unifacial trapezoidal scrapers (2–4, 6, 12–16, 19, 20, 22), leaf-shape retouched points (5, 7, 17, 18) and plano-convex bifacial scrapers (8–11, 23–25).

Both assemblages also include artefacts manufactured from jasperoids, the highest quality raw material used in the Altai Middle Palaeolithic, and together represent the most eastern manifestation of the KMG technocomplex in the Altai Mountains[1].

We consider it likely that the Neanderthal groups at Chagyrskaya and Okladnikov Caves were broadly contemporaneous, given (a) the geographical proximity of these two sites (**SI Figure 1.1 A**), (b) the exceptional typological and technological similarities of their lithic assemblages and their unique (Sibiryachikha variant) attributes (**SI Figure 1.6**), and (c) the potential overlap in timing of their occupation by Neanderthals (>44 ka at both sites and <92 ka at Chagyrskaya Cave). A close association between the two groups is supported also by the genetic similarities of the Okladnikov and Chagyrskaya remains examined in the present study, although they do not comprise a single population (based on a statistical analysis of the average number of genetic differences between any two Chagyrskaya remains compared to pairs of Chagyrskaya and Okladnikov remains; **SI 7 - Relationship between Chagyrskaya and Okladnikov remains**). Both caves, therefore, appear to have been occupied at a similar time by two groups of late Neanderthals that were closely related genetically.

Neanderthal remains

We provide a complete list of the anatomical remains used in this study in **Extended Table 1**.

Age estimates for remains

Two different approaches for the estimation of age using dental development are used in anthropology: tooth formation and tooth eruption. Of these, tooth formation is less dependent on environmental factors (for an overview see[25] and [26]), and for this reason was used where possible.

First, the development of each tooth was scored according to the stages devised by Moorees and colleagues[27]. This scoring system consists of six stages for the crown formation, five (for single-rooted teeth) or six (for molars) stages for root formation and two stages of apex closure. Age estimates for the formation stages are the modified Moorrees, Fanning and Hunt data from Shackleford and colleagues[28]. Where separate values for males and females were available, the genetic sex estimate was taken into account.

These studies of tooth formation use recent populations of European ancestry, and thus are not easily applicable to other geographic and temporal groups. Our understanding of the reasons and factors responsible for variability in tooth formation is still very limited, but as shown by Liversidge[29] these differences are not substantial. Nevertheless, it is important to keep in mind that the ages determined here are simply the ages of modern children with teeth at comparable developmental stages.

Over the last years, histological and synchrotron analyses of dental microstructures allowed us to calculate the age of several Neanderthal children with much higher precision (e.g. [30–32]). Though such data are not available for the Chagyrskaya assemblage, we compared these teeth to the developmental stages of Neanderthal individuals of known age.

Dental wear was scored following Molnar[33]. There are severe limitations to using dental wear on isolated teeth for age estimation, especially in fossil hominins, but in some larger assemblages this approach allowed not only to seriate dental remains by age, but even to attempt to estimate their ages[34]. An additional complication is that Neanderthal dentitions usually show strong wear, and a pattern of wear that differs from recent populations ([35,36]).

Chagyrskaya remains

Chagyrskaya 1 (SI Figure 1.7a,b)

Left lower deciduous canine, naturally exfoliated.

Shackelford and colleagues ([28], based on [27]'s data) provide 10.67 years (8.64-13.11, ± 2SD) as the mean for exfoliation of the lower canine in males. This fits well with Hillson's[37] estimate of 10-10.6 yrs for gingival emergence of the lower canine in recent populations.

The presence of an erupted lower permanent canine (and thus the shedding of the deciduous canine) in the Scladina juvenile, aged about 8 years old[30,31] indicates that a somewhat lower age is also possible.

Chagyrskaya 2 (SI Figure 1.8c)

Immature atlas fragment, left side.

Age estimation: both posterior and anterior synchondrosis are open, these start fusing around the age of 4-5 yrs for the posterior arch and 5-6 yrs for the anterior arch[38].

Chagyrskaya 6 (SI Figure 1.8d)

Right hemimandible with C-M_2

Age: The roots of all available teeth are fully developed, and the third molar, though not preserved, must have been erupted as shown by the distal interproximal facet on the M_2. The teeth are in the following wear stages (using the scale of [33]): canine – 4, P_3–3, P_4–3, M_1–4, M_2–3. Compared to other adult dental remains from the cave, the wear on these teeth is relatively low, which could indicate that this is a young adult individual. The strong alveolar resorption on the buccal side and the formation of a "peripheral buttress" on the lingual side are signs of periodontal disease, which is not so frequent in young adults, but could be linked to dietary or other oral health factors.

Chagyrskaya 7

Vertebral process fragment. Probably adult based on size.

Chagyrskaya 8 (SI Figure 1.8a)

Manual distal phalanx. For a more detailed description see [2], SI 1.

Age: Epiphyses fused, no epiphyseal line visible in the µCT scans. Adult.

Chagyrskaya 9 (SI Figure 1.8e)

Left ulna, proximal fragment

remain described in detail in[39].

Adult, based on completely fused proximal epiphysis. The presence of large osteophytes on the olecranon could indicate a somewhat higher age, but the lack of pathological changes to the articular surfaces fit better with a younger adult. [39] interpreted the exostoses as possibly indicative of diffuse idiopathic skeletal hyperostosis (DISH), also known as Forestier's disease.

Chagyrskaya 12 (SI Figure 1.7c,d)

Left lower P_3

Wear: Wear in general very similar to P_3 of *Chagyrskaya 6*. Large dentine exposure on protoconid, 5.5 (MD) x 1.4 (BL). Two wear facets labially of the dentine exposure, with the mesial somewhat larger. 2.5x1.9 IPF mesially, 4.6 x 2.8 distally.

Age:

Fully formed roots with a closed apex give a minimum age of over 12 years ([28], female average for apex closure 12.24, 9.98-15.0 ± 2SD). Wear Molnar stage 3, comparable to *Chagyrskaya 6* mandible. Adult, likely young.

Chagyrskaya 13 (SI Figure 1.7m,n)

Left upper I^1

Wear: Medium, dentine exposed along incisal edge, but not along mesial and distal marginal ridges.

Age: Roots fully developed and distal half covered in cementum. The surface is damaged on the apical third of the root, with cementum and dentine flaking away on the mesial and labial surface. An apical foramen of about 0.5 mm diameter remains, but it is not clear whether it has been enlarged by the damage, or not yet fully closed. In the µCT scans (see **SI Figure 1.9 a,b**), the pulp cavity tapers towards the apex, but remains relatively wide until it exits the root. The most likely developmental stage is the apex half closed (A ½). The crown is moderately worn, with dentine exposed along the whole incisal edge but not on the mesial or distal marginal ridges.

In recent populations, the I^1 completes root formation by 8.6 yrs ([28], males, 7-10.6 ± 2SD). Apex closure happens around 9.8 yrs [40]. The wear in this tooth is stronger than that seen in recent ten-year-olds, and would in most modern human populations only be seen in an adult.

In Neanderthals, the I^1 apex is closed in the 8 year old Scladina individual[30,31], indicating a faster root formation than in recent humans. Interestingly, the wear seen in Scladina, and the 11.6-12.1 year old *Le Moustier 1* juvenile is less than that in *Chagyrskaya 13*, though still much stronger than in recent juveniles, but the strong anterior dental wear in Neanderthals has been recognized for a long time[35,41,42].

Chagyrskaya 14 (SI Figure 1.7k, l)

Lower left I_2

Wear: About half of the crown worn away, remaining crown height labially 5.4 (in projection, parallel to tooth axis). Distal IPF placed close to the labial margin of distal surface, 1.9x1.9. Mesial IPF less distinct, but seems to extend further labially. Molnar wear stage 4.

Age: Based on wear an adult, assuming higher dental attrition in Neanderthals likely a young adult. Wear stage fits well with that expected for the incisors of the *Chagyrskaya 6* mandible, in agreement with the genetic data.

Chagyrskaya 17 (SI Figure 1.7e,f)

Right lower P_4

Wear: Slight. Wear facets on protoconid and metaconid, polish on accessory cusps. IPFs visible, but not very marked, probably did not remove much material.

Age: Slight wear, with facets on protoconid and metaconid, and polish on accessory cusps (Molnar Stage 2). Roots fully developed and apex closed, giving a minimum age of about 14 years ([28], female average for apex closure 13.74, 11.2-16.8 ± 2SD). Taking into account the only slight wear, this individual is probably a (late) adolescent.

Chagyrskaya 18 (SI Figure 1.7q,r)

Left upper dm^1

Age: Whole crown is strongly worn, with dentine exposed over the whole crown. Roots are mostly resorbed, with the longest root remnant below the mesiobuccal corner of the crown, likely leading to natural exfoliation. Shackelford and colleagues[28] provide 10.1 years (8.2-12.4, ± 2SD) as the mean for exfoliation of the first deciduous molar in females, but it is important to keep in mind that the original data used for the study[27] was collected on lower deciduous molars. Hillson ([37], reporting data from [43] and [44]) provides an average age of 9.4-9.7 yrs for the gingival eruption of the upper P^3, which happens after the exfoliation of the dm^1.

Of the Neanderthal individuals for which we have more precise age estimates, the 8 year old (([30,31])) Scladina I-4A juvenile's dm1 is in root resorption stage Res3/4[32], so somewhat less resorbed than *Chagyrskaya 18*. The time period from stage Res3/4 to exfoliation is approximately 0.7 years in the [27] dataset. Thus, an age of about 9-10 years for *Chagyrskaya 18* seems plausible.

Chagyrskaya 19 (SI Figure 1.7s,t)

Left upper dm2

Age: Strong wear. Large dentine exposures on the protocone and paracone confluous, somewhat smaller exposures on the hypocone and metacone. Roots mostly resorbed, with approximately 3.5 mm remaining mesiobuccally, 2.9 mesiolingually, 0.7 distobuccally and 1.4 distolingually (all measured from the cementoenamel junction). In the distal part of the crown resorption has reached the cervix, indicating that the tooth probably exfoliated naturally. The

[27] dataset, as reported by [28] has an average age at exfoliation of 11.6 yrs (9.4-14.3, ± 2SD), but this is based on lower molars. This is somewhat older than the average age for gingival emergence for the P^4 reported by [37], which is 10.7-11.1.

The 8 year old Scladina I-4A individual ([30,31]) again serves as a good comparison. The roots of the dm^2 in this case are approximately halfway resorbed. Taking into account the approximately two to two and a half year difference seen between the Res1/2 and exfoliation stages seen in the [27] dataset, an age of approximately 10 years (9-11) at the exfoliation of the dm2 seems reasonable.

Chagyrskaya 20 (SI Figure 1.7u,v)

Right upper deciduous canine

Age: The roots of this exfoliated deciduous canine are resorbed to the cervix on the lingual side, while about 1.5 mm of the root remain labially. Exfoliation of the canine according to Moorrees and colleagues' data ([27], as listed in [28] occurs at 9.5 years in recent females (7.7-11.8 ± 2SD), but this data is for lower canines. As the eruption of the upper canine lags the lower canine by between six months and a year depending on population (see [29], Table 4.4), it is probable that deciduous root resorption and exfoliation also happens somewhat later in the upper jaw. Upper canines erupt between about 9.8 and 11 years in recent females (averages for different populations, [29]), making an age of around 10 years (9-11) based on modern standards likely.

We have little data on when the upper deciduous canine exfoliated in Neanderthals. The eight-year-old Scladina individual had the upper canine erupted and with the root almost fully formed ([30,31]), indicating that the deciduous canine was lost well before this age. The 6-8.1 year old [45] Obi-Rakhmat 1 individual's upper canine does not show any evidence of occlusal wear or polishing (own observations, [46]), and the quarter developed root (Moorrees stage R¼) makes it likely that it still retained the deciduous canine. At the same time, the advanced development of the canine root probably indicates that exfoliation would have happened earlier than in the recent samples, probably within the year following the death of the individual. Bearing in mind this evidence for an accelerated dental development of the upper canine in Neanderthals, an estimate of about 7-10 years seems more likely.

Chagyrskaya 41 (SI Figure 1.7g,h)

Right lower P_3

Age: The crown of this P_3 shows extensive wear, removing most occlusal relief. There is a large dentine exposure on the protoconid, and a very large distal interproximal facet, exposing dentine over most of the mesial surface of the crown (this is exaggerated by some enamel breakage along the cervix). The strong wear indicates that this individual is probably older than *Chagyrskaya 6, 17* and *50*. Adult.

Chagyrskaya 50 (SI Figure 1.7i,j)

Left lower P_3

Both the crown and the approximately half of the root remaining are strongly chemically eroded, possibly due to digestion.

Age: The crown shows significant wear, exposing an about 5x 1.4 mm area of dentine on the protoconid. The wear is stronger than that seen on *Chagyrskaya 6* and *17*, but less than on *Chagyrskaya 41*, possibly indicating a somewhat younger, but still adult individual.

Chagyrskaya 63 (SI Figure 1.7o,p)

Left upper M^2

Age: The roots are broken just below the cervix, and thus the developmental stage of this tooth can not be assessed. It is clear though that it was erupted, as there is polish on the mesial cusps and a clear wear facet on the protocone.

M^2 eruption occurs between on average 10.9 and 13.2 years of age in recent humans, depending on population, but differing definitions of eruption complicate the issue [29]. Few studies report the time span between clinical eruption and the time the tooth comes into occlusion, but for the lower M_2 for example, [29] found almost three years between alveolar eruption (9.29 yrs) and occlusal eruption (12.25 yrs).

The situation is complicated by the presence of the lower M_2 in occlusion in the eight year old Scladina Neanderthal[30,31]. The eruption of the lower M_2 precedes the eruption of the upper M^2 by about half a year in the data reported by [29] slightly varying by population. This also fits with the advanced development of the upper M^2 in Scladina, which is at developmental stage (Root ¼) usually seen in recent children about a year older. If the upper M^2 in at least some Neanderthals erupted earlier than in present day populations, then *Chagyrskaya 63* could derive from an individual as young as 9-10 years and up to about 12-14 years.

Chagyrskaya 60 (SI Figure 1.8b)

Manual intermediate phalanx.

Age: Epiphyses fused, no epiphyseal line visible in the µCT scans. Adult.

Age estimation of associated Chagyrskaya remains

The Chagyrskaya assemblage contains three associated sets of remains, based on the genetic data.

The first of these is *Chagyrskaya 8*[2], a distal manual phalanx and *Chagyrskaya 12*, a lower P_3. Both of these are fully developed, with fused epiphyses in the phalanx and a closed root apex in the P_3. The relatively light wear of the P_3, comparable to that seen in the *Chagyrskaya 6* mandible, indicates that this was a relatively young individual, either a late adolescent or a young adult. These remains were found in the same stratigraphic level, Layer 6b horizon 2, and relatively close together in squares N10 and O12.

The second set are the *Chagyrskaya 6* mandible fragment and *Chagyrskaya 14*, a left lower incisor. They are compatible in wear, and likely also derive from a young adult, based on the erupted M_3 (as evidenced by a distal IPF on the M_2), but relatively slight wear on the M_2. The remains derive from the same square (N10) and stratigraphic level (6b horizon 3).

The third set consists of three teeth, the *Chagyrskaya 13* I^1, the *Chagyrskaya 19* dm^2 and the *Chagyrskaya 63* M^2. As outlined in the individual descriptions, the dental development of this individual is comparable to a recent 10-12 year old, with the exception of the relatively strong occlusal wear on the I^1. Among Neanderthals, the 8 year old[32] Scladina I-4a individual is the closest parallel. Scladina shows a more developed (apex closed instead of partly open), but less worn I^1, and slightly less resorbed dm^2 roots, and an unerupted M2, indicating that it might be somewhat younger than the Chagyrskaya individual. The almost completely resorbed roots of *Chagyrskaya 19* suggest that it was exfoliated naturally, but it is also possible that the individual died shortly before exfoliation would have happened.

Chagyrskaya 13 was found in Sq. O12 (Layer 6b, Hor.2), *Chagyrksaya 19* in O11 (Layer 6a) and 63 in Sq. I7 (6A). While 13 and 19 were found close together, even though in different layers, 63 was found about 8 m away, both indicating postdepositional transport.

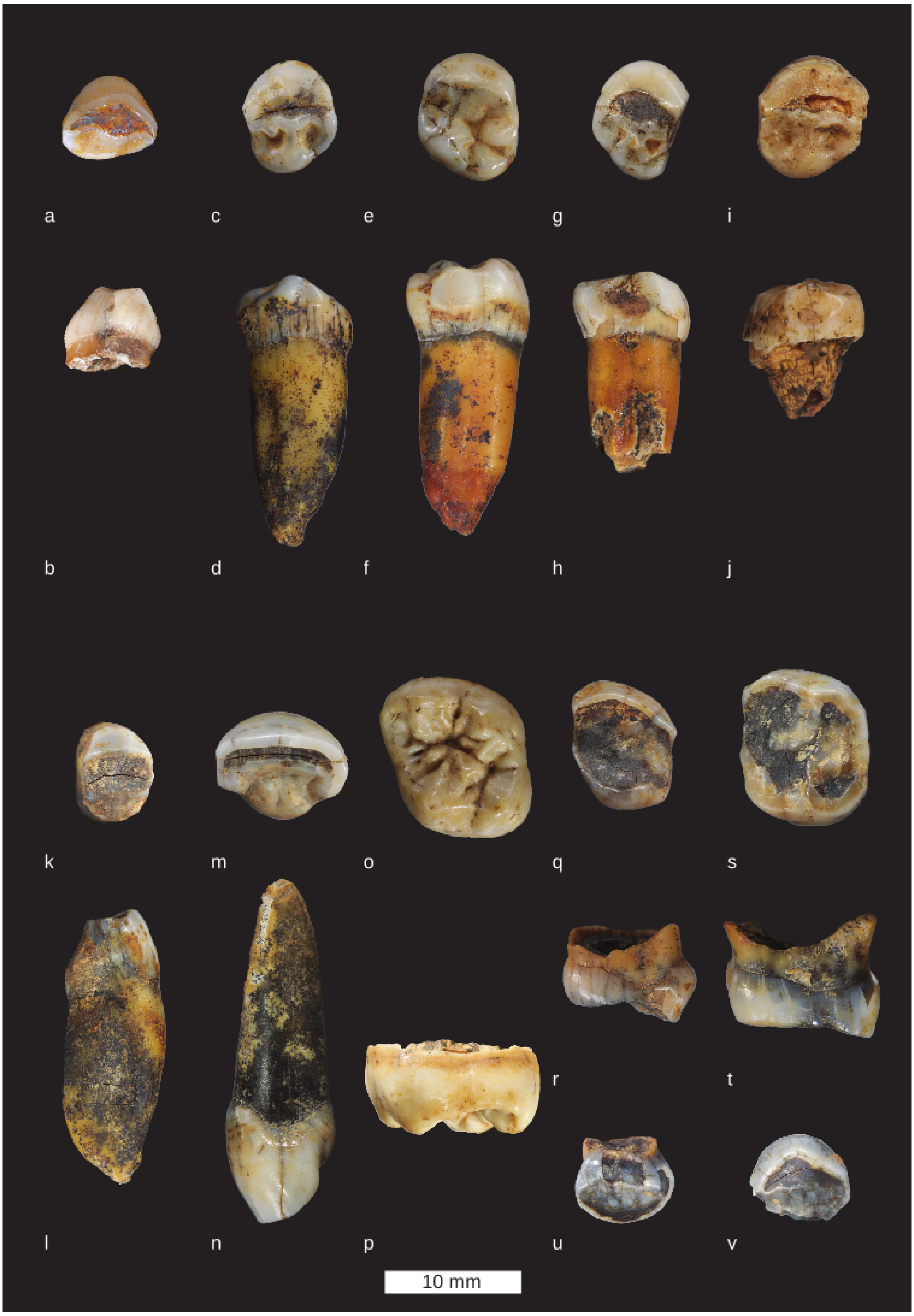

SI Figure 1.7: The dental remains from Chagyrskaya Cave used in this study. a. *Chagyrskaya 1*, occlusal view; b. *Chagyrskaya 1*, mesial view; c. *Chagyrskaya 12*, occlusal view; d. *Chagyrskaya 12*, mesial view; e. *Chagyrskaya 17*, occlusal view; f. *Chagyrskaya 17*, distal view; g. *Chagyrskaya 41*, occlusal view; h. *Chagyrskaya 41*, distal view; i. *Chagyrskaya 50*, occlusal view; j. *Chagyrskaya 50*, mesial view; k. *Chagyrskaya 14*, occlusal view; l. *Chagyrskaya 14*, mesial view; m. *Chagyrskaya 13*, occlusal view; n. *Chagyrskaya*

13, distal view; o. *Chagyrskaya 63*, occlusal view; p. *Chagyrskaya 63*, distal view; q. *Chagyrskaya 18*, occlusal view; r. *Chagyrskaya 18*, distal view; s. *Chagyrskaya 19*, occlusal view; t. *Chagyrskaya 19*, mesial view; u. *Chagyrskaya 20*, lingual view; v. *Chagyrskaya 20*, occlusal view

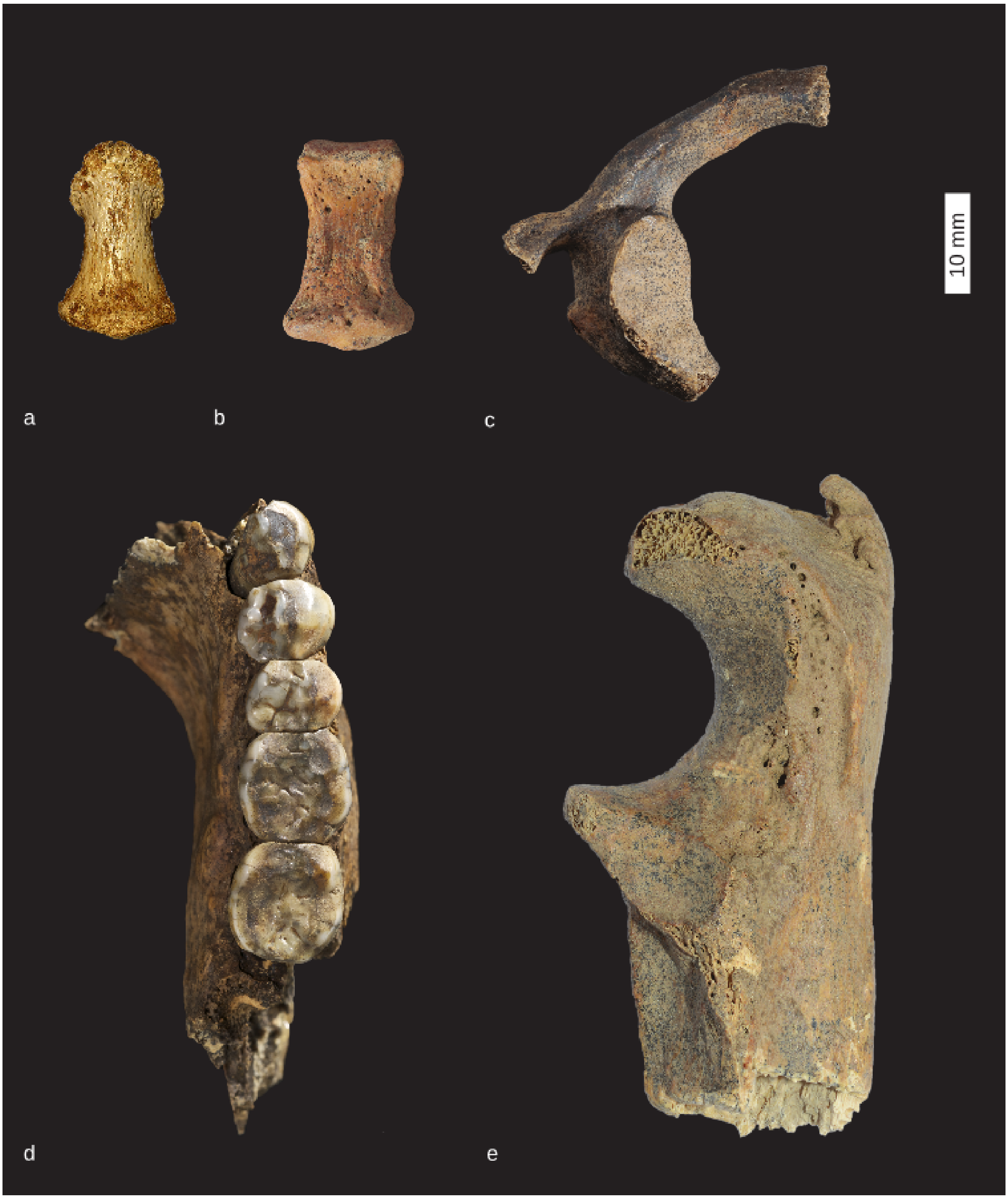

SI Figure 1.8: Other remains used in this study. a. *Chagyrskaya 8*, dorsal view; b. *Chagyrskaya 60*, palmar view; c. *Chagyrskaya 2,* inferior view; d. *Chagyrskaya 6*, occlusal view; e. *Chagyrskaya 9*, lateral view

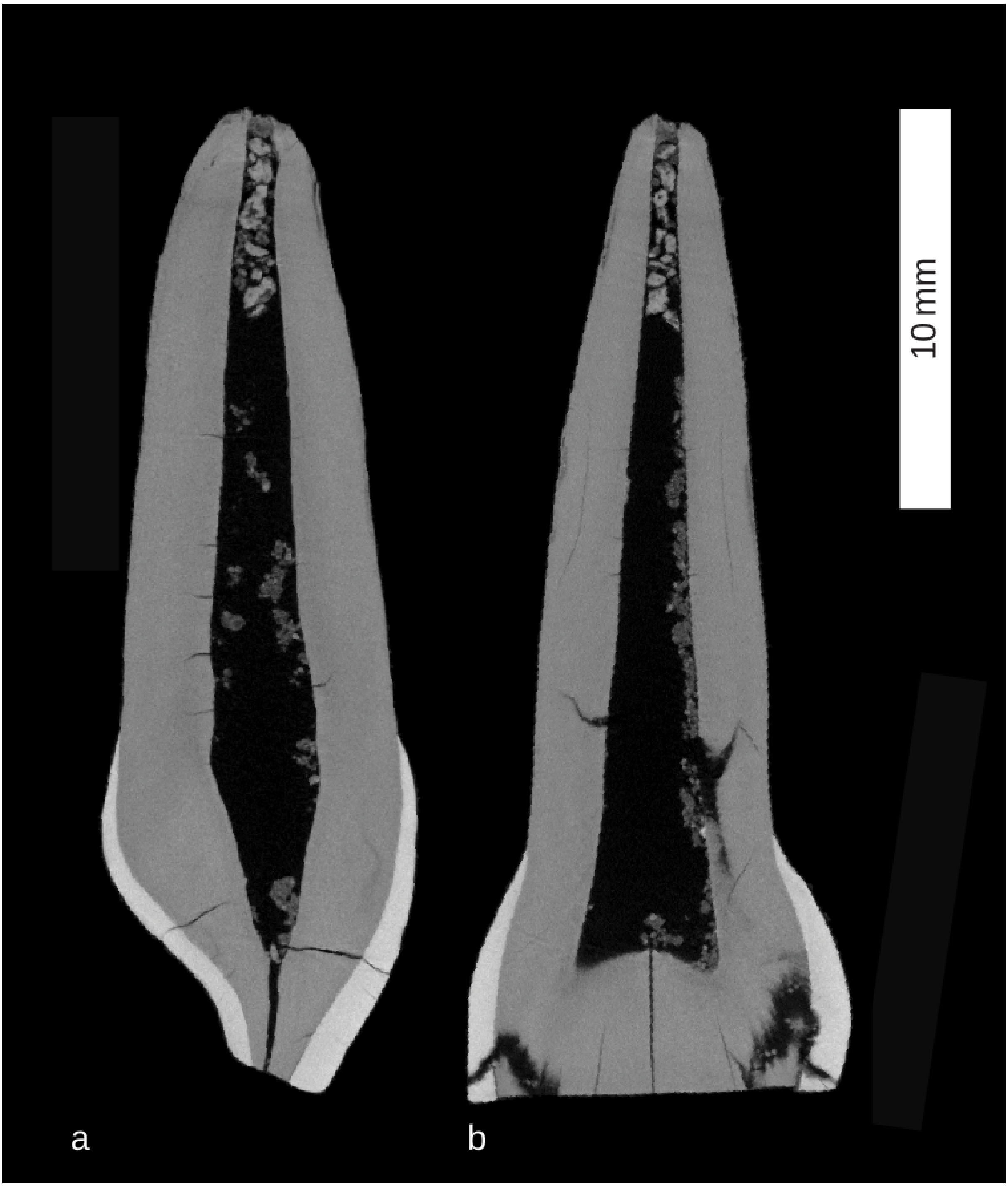

SI Figure 1.9: µCT sections through the *Chagyrskaya 13* incisor.

Okladnikov Cave remains

Okladnikov 11 (S-84/276, SI Figure 1.10a)

Right proximal femur fragment

The proximal half of a juvenile femur, preserving the neck but lacking the epiphyses of the head and greater and lesser trochanter. The remain was described[47].

Age: The remain is slightly smaller [47] than the femur of Teshik-Tash (about 9-11 years old, but estimates vary, see [48]), and also fits with recent children in the 8-10 year old range. The unfused epiphyses allow us to give a maximum age estimate, as fusion of the femoral head epiphysis starts around 12-14 years in recent females, while around 14-16 in recent males [38]. The presence of well developed muscle insertions, including the gluteal tuberosity and spiral line allow a minimum age estimate, as these start developing around 7-8 years in recent children[38]. An estimate of 8-10 years seems likely.

The distal portions of the preserved part of the shaft show a porous subperiosteal bone growth. Such periosteal reactions are not specific, and can be caused by various pathological processes such as anemia, scurvy, rickets, subperiosteal haematomes and various inflammatory processes[49].

Two other remains from Okladnikov cave, the right distal humerus *Okladnikov 14* (S-84/164, **SI Figure 1.10d**) and the left distal femur *Okladnikov 1* (S-84/349, **SI Figure 1.10b**) are potentially associated with this remain. *Okladnikov 1* was found in the neighbouring square meter, but in the overlying horizon 2, while *Okladnikov 14* was found about 5 m away in the same layer as 11. The distance, and the difference in stratigraphic provenance could be probably explained by postdepositional processes in the shallow deposits of the grotto.

Both *Okladnikov 1* and *14* derive from children of a comparable age: [50] estimated 9-12 years for *Okladnikov 14*, and 8-10 years for *Okladnikov 1*; [47] 8-10 years and 7-8 years, respectively.

Okladnikov 1 also shows subperiosteal appositions on the posterolateral surface of the femoral shaft, around the midshaft level, similar to *Okladnikov 11*, supporting the assumption that these two remains belong to the same individual. No such changes are visible on *Okladnikov 14*, but this remain shares the mtDNA sequence with *Okladnikov 11*[51]- when excluding the Dloop.

Okladnikov 15 (S-84/78, SI Figure 1.10c)

Right distal humerus fragment

A small right distal humeral fragment, originally broken along the proximal border of the olecranon fossa. The remain was described by [50] and [47].

Epiphyseal fusion is complete, the epiphyseal line is not visible on the surface nor in µCT scans. In modern humans, epiphyseal fusion of the distal humerus happens at about 11-15 years in females and 12 to 17 years in males[38], so the individual must be older than this, and the absence of any visible traces of the separation indicates that the individual is probably

over 20 years. The absence of any pathological alterations of the articular surface suggests a relatively young age.

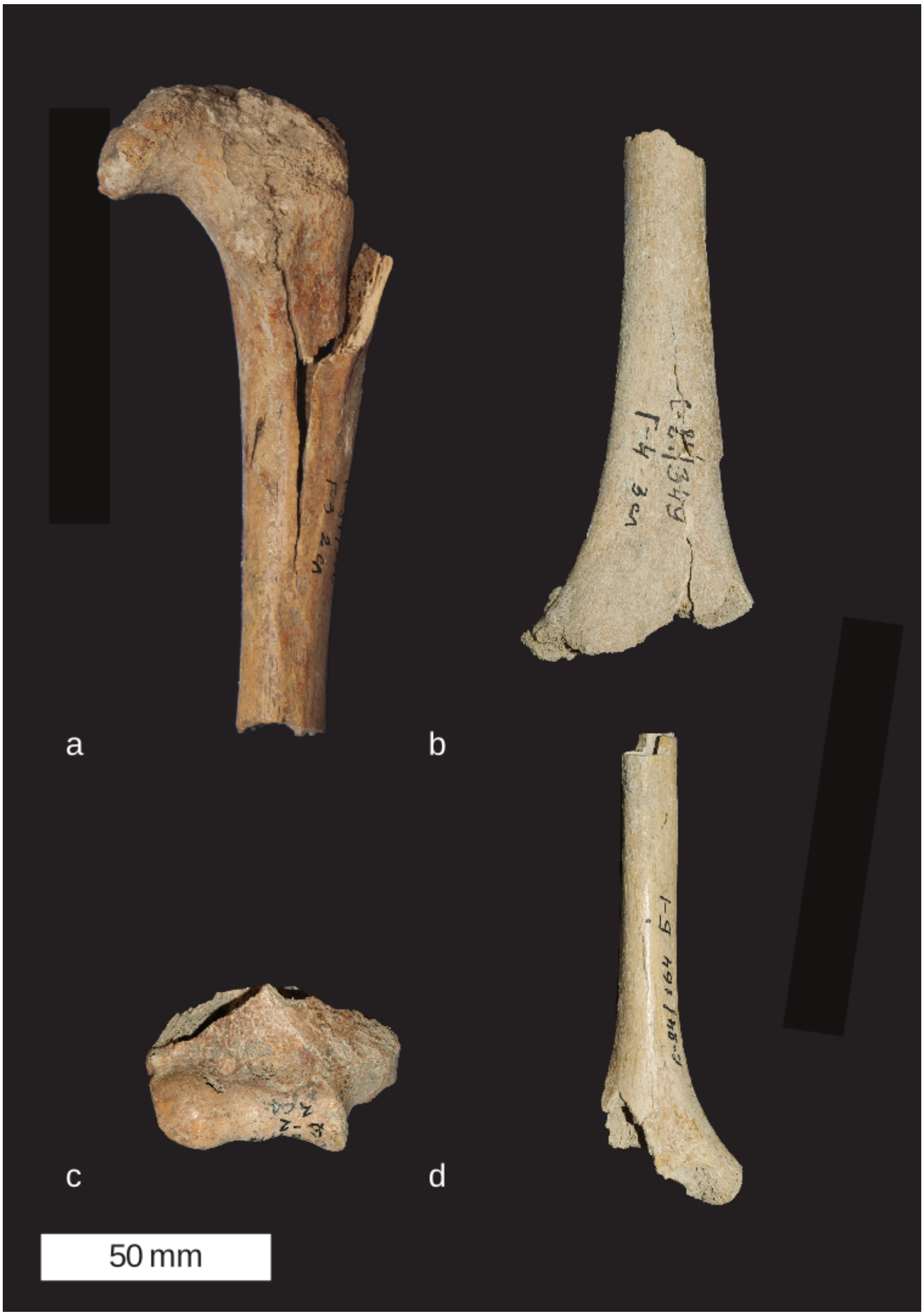

SI Figure 1.10: The remains from Okladnikov Cave discussed in this study. a. Okladnikov 11, posterior view; b. *Okladnikov 1*, anterior view; c. *Okladnikov 15*, anterior view; d. *Okladnikov 14*, anterior view

SI 2 DNA extraction, lib prep, sequencing

DNA Extraction and library preparation

The remains from Chagyrskaya and Okladnikov Caves were sampled in the dedicated clean room facility of the Max Planck Institute for Evolutionary Anthropology in Leipzig, Germany. For each remain, a thin layer of approximately 1 mm of surface material was removed from the selected sampling area using a sterile dentistry drill. In a series of experiments, between 1 and 63.8 mg of tooth or bone powder from Chagyrskaya remains, and between 5 and 59.3 mg of bone powder from Okladnikov remains, were obtained by drilling (**SIDataset1 - Initial shotgun tab**). For some of the remains, DNA was extracted from the surface powder as well (SIDataset1).

Following a previously described protocol for extraction of highly degraded DNA, the sampled material was digested in either 300 µl, 500 µl or 1,000 µl of lysis buffer, depending on the input amount of tooth or bone powder, and the DNA was subsequently purified either manually from the entire lysate[52,53]) or using an automated version of the protocol from 150 µl aliquots using buffer ‘D’ for DNA binding[53]. The extracts were then converted into single-stranded DNA libraries either manually[54] or using an automated protocol[55]. Negative controls were carried throughout all laboratory steps to monitor levels of present-day DNA contamination.(**SIDataset1 - list of negative controls tab**). We used quantitative PCR or digital droplet PCR assays to measure the number of unique molecules in each library[54], and to evaluate the efficiency of the library preparation based on the conversion of a control-oligonucleotide spiked into each reaction[56]. The libraries were amplified and barcoded using a unique pair of either 7 or 8 base pair (bp) indices[57] with modifications as described elsewhere[52], see[55] for the automated protocol). Amplified libraries were then purified, pooled, and shotgun sequenced on a MiSeq or HiSeq 2,500 Illumina machine in double-index paired-end configuration (2x76 or 2x75 cycles plus 2x7 or 2x8 cycle index reads). Automated laboratory procedures were carried out on an Agilent Bravo NGS Workstation.

In addition to the described DNA extractions performed with no prior pre-treatment of sampled powder, we also applied at least one of three DNA decontamination strategies[52] to sampled bone or tooth powder. These strategies can result in a reduction of the microbial and/or present-day human DNA contamination frequently found in ancient remains. Between 19.3 and 33.5 mg of bone powder were treated with 0.5% sodium hypochlorite solution prior to extraction (**SIDataset1 - Initial shotgun tab**). Moreover, fractions of approx. 30 mg of collected powder from *Chagyrskaya 9* were each pre-treated with different methods: either with a 5-minute pre-digestion in lysis buffer, followed by the re-extraction of the bone pellet (modified from[58]); or by washing with 0.5M sodium phosphate solution[52] (SIDataset1 - Initial shotgun/Data generation setup). Thereafter, DNA was extracted, converted into single-stranded DNA libraries, and sequenced following the same procedures as above.

Initial shotgun sequencing

For processing the shotgun sequence data, we used *Bustard* (Illumina) to perform base calling and *leeHom* to trim adapters and merge overlapping paired-end reads[59]. The Burrows-Wheeler Aligner (BWA[60]) with parameters adjusted for ancient DNA was used to

align sequences to the human reference genome GRCh37 from the 1000 Genomes project, which additionally includes the revised Cambridge Reference Sequence (rCRS; NC_01290), the 1000 Genomes Phase 2 decoy sequences, the ΦX174 genome (NC_001422), and the human herpesvirus (NC_007605). We then assigned sequences to individual libraries by requiring a perfect match of the index sequences to the expected combinations. PCR duplicates were removed using *bam-rmdup* (https://github.com/mpieva/biohazard-tools/; version 0.2), and sequences shorter than 35 bp and with a mapping quality lower than 25 were excluded from downstream analyses in order to avoid spurious alignments originating from microbial sequences.

The ends of ancient DNA molecules are prone to carry uracils (U) arising over time through deamination of cytosines (C[61]). Uracils are subsequently read as thymines (T) by DNA polymerases during single-stranded library preparation, leading to apparent C>T substitutions at the ends of ancient DNA sequence alignments. Therefore, the elevated frequencies of C>T substitutions at the 5' and 3' ends of sequences can be used to evaluate the presence of DNA of ancient origin in a given sample. The frequencies of C>T substitutions in the libraries prepared from extracts of the Chagyrskaya and Okladnikov remains (**SIDataset1**) indicate the preservation of ancient DNA molecules in most of the analysed remains. However, there is varying degree of preservation among libraries and/or applied DNA extraction pre-treatments. We screened 113 libraries from 27 remains but only found evidence of ancient DNA preservation in 85 libraries from 17 remains (**Extended Table 1**).

Additional shotgun sequencing

We deeply sequenced eight libraries of the *Chagyrskaya 7* remain, six of which had first undergone a size selection procedure using a denaturing polyacrylamide gel to remove fragments shorter than 35bp[55] . The results are shown in **SIDataset1 - Additional shotgun sequence tab**.

SI 3 Mitochondrial (mt) DNA captures and sequencing

Captured libraries

An aliquot of each amplified library was additionally enriched for human mitochondrial DNA (mtDNA) through two successive rounds of on-bead hybridization capture[62] or in-solution hybridization capture[63], following an automated protocol described elsewhere[64], using probes that were generated based on rCRS[65] and printed on a microarray[66]. The enriched libraries, along with corresponding negative controls, were pooled, sequenced, and processed as described above for the generation of shotgun sequences, with the difference that the generated sequencing data from these libraries were aligned to the rCRS.

In total, we enriched 85 libraries from the 17 remains for human mtDNA - see **SIdataset1 - MT capture tab**.

The number of mitochondrial DNA sequences generated from each library and the frequency of their C>T substitutions at the 5' and 3' ends are presented in **SIdataset1 - MT capture tab**. The elevated C>T substitutions provide evidence for the presence of mitochondrial sequences of ancient origin in most of the libraries prepared from the Chagyrskaya and Okladnikov remains. The negative control libraries did not exhibit evidence for the presence of ancient DNA as expected (no more than 13 reads with a C>T or G>A substitution in the first/last 3 positions of the read were observed in a single library; in addition, the lower bounds of the 95% binomial confidence intervals of the C>T substitutions frequencies were consistently below 10%; see **SIDataset1 - MT capture tab**).

We filtered all BAM files for DNA fragments longer than 35 bp (L>=35) and mapped them to the rCRS [65] with a mapping quality of at least 25 (MQ>=25).

Modern human contamination estimates

To estimate the levels of present-day human DNA contamination, we estimated the proportion of sequences that carry the modern human allele at 47 positions where mitochondrial genomes of 312 present-day humans ([67]; including rCRS) differ from all 24 Neanderthals whose mitochondrial genomes have been reconstructed previously[2,51,67–77].
We report contamination estimates for each library in **SIdataset1 - MT capture tab.** For most remains, we used data only from libraries estimated to contain less than 20% contamination, since we require 80% of sequences to agree for consensus calling. However for *Chagyrskaya 9* we also included two libraries (R1919 with contamination estimate of 30% and R1921 with contamination estimate of 22.9%) to increase the number of reads. For the Okladnikov remains, we only used deaminated reads (i.e., reads displaying a C>T change at the first 3 positions or 3 last position) for consensus calling, to reduce the effect of the high contamination rates estimated (mean contamination estimate of 58% for *Okladnikov 11* and 65% for *Okladnikov 15*).

Reconstruction of the mitochondrial genomes

To reconstruct the mitochondrial genomes of the Chagyrskaya and Okladnikov remains and avoid reference bias to a distantly related reference sequence, we re-aligned the sequences from all 17 remains to the mitochondrial genome of *Chagyrskaya 8*[2] and processed the data as described in **SI 2**.

We then called a consensus base at each position covered by at least five sequences and where at least 80% of the sequences carried the same allele. We masked all Ts in the first three and/or last three positions in forward reads, and all As in the first three and/or last three positions in reverse reads, as these may represent errors due to ancient DNA damage. For each Chagyrskaya remains, we called two consensus genomes, one using all sequences and a second one using only sequences with at least one C>T substitution within the three first and/or last positions from the alignment ends. As the two consensus genomes for each remain were identical at all positions where we could call a base, we retained the consensus genomes reconstructed using all sequences for subsequent analyses.

We found that the consensus of *Chagyrskaya 9* was unresolved at 75 positions due to a low consensus support (i.e., <80% of sequences have the same allele) despite the high coverage of 263-fold. A closer inspection of the coverage distribution along the mitochondrial genome of *Chagyrskaya 9* revealed regions where the coverage reached up to 1,574-fold (compared to an average coverage of 263-fold for the full mitochondrial genome; **SI Figure 3.1**), suggesting the presence of potentially exogenous sequences aligning to these regions. Further investigation of the recovered mtDNA sequences from *Chagyrskaya 9* using Blast [78] showed a high percent of sequences matching the *Crocuta crocuta* (spotted hyena) mitochondrial genome, thus indicating the presence of hyena DNA contamination. This is consistent with previous observations of hyena mtDNA contamination on other Neanderthal remains from the Altai region [79].

Therefore, we re-aligned the sequences of *Chagyrskaya 9* to both rCRS and the mitochondrial sequence of a hyena (isolate Kira, JF894377.1). We then only used the sequences that mapped to rCRS in this combined reference and processed the data as described for the other remains. After remapping, only 34 positions remained unresolved and the coverage depth decreased from an average of 263- to 247-fold, with a maximum coverage depth of only 575-fold, suggesting that most hyena DNA sequences were effectively removed with this strategy **SI Figure 3.1**. We do not observe a similar increase in coverage in other Chagyrskaya or Okladnikov remains indicating that if there is contamination in these remains it is to a much lesser extent compared to *Chagyrskaya 9*.

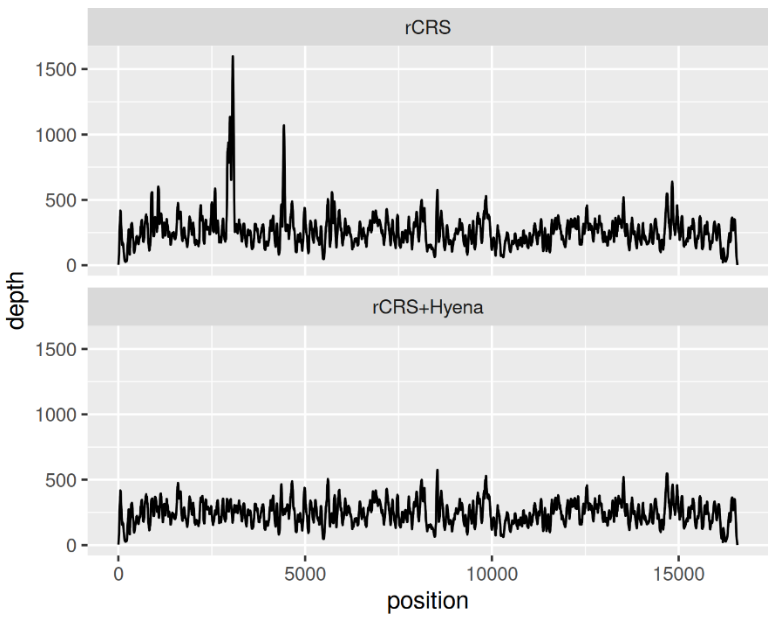

SI Figure 3.1: Coverage depth in the *Chagyrskaya 9* dataset for sequences aligned to the revised Cambridge Reference Sequence (rCRS; upper panel) or the rCRS part of a concatenated reference sequence including rCRS and a Hyena mitochondrial genome (JF894377.1, lower panel).

The varying levels of present-day human DNA contamination in *Chagyrskaya 9* and the other Chagyrskaya remains may explain the low consensus support (<80%) observed at some of the still unresolved positions when using all sequences. At these positions, we introduced in our reconstructions the base that had at least 80% consensus support among putatively deaminated sequences exhibiting at least one C>T substitution within the first three and/or last three positions. Present-day human DNA sequences should be rare in these subsets of the data, as shown from the contamination estimates (**SI Dataset1 - MT capture tab**).

After editing these positions, a maximum of five positions remained unresolved for the mitochondrial genomes of the Chagyrskaya remains, with the exception of *Chagyrskaya 6* and 9, with 655 and 7 unresolved positions, respectively. Because the analysis of the nuclear DNA revealed that *Chagyrskaya 6* and *14* belong to the same individual (see **SI 7 - Identical individuals**), we compared the reconstructed sequences of these two remains and found no differences among the positions called in both remains.

Finally, the remaining five unresolved positions in the genomes of different remains were either in regions difficult to align due to their repetitive content, or represent heteroplasmies and artefacts, which are not accounted for in a consensus call. We resolved these positions in the next section.

Curated data set

In **SI Table 3.1** we show a summary table for the curated merged BAM files and their respective fasta consensus sequences.

remain name	contamination (%)	contamination 95% CI (%)	genome size	average coverage	n reads	Ns in consensus sequence
Chagyrskaya 1	2.48	2.30-2.67	16,569	1,832	565,540	2
Chagyrskaya 2	0.56	0.53-0.60	16,569	7,247	1,665,859	1
Chagyrskaya 6	1.49	0.48-3.44	16,569	23	6,645	655
Chagyrskaya 7	1.3	1.08-1.55	16,569	612	173,385	2
Chagyrskaya 9	12.18	11.15-13.26	16,569	247	78,200	7
Chagyrskaya 63	2.06	1.91-2.21	16,569	2,299	706,654	3
Chagyrskaya 12	0.69	0.58-0.82	16,569	1,414	473,003	1
Chagyrskaya 13	0.66	0.60-0.72	16,569	4,323	1,214,776	2
Chagyrskaya 14	4.88	4.53-5.24	16,569	1,030	343,147	1
Chagyrskaya 17	5.81	5.50-6.13	16,569	1,630	559,227	1
Chagyrskaya 18	0.3	0.24-0.38	16,569	1,838	568,586	0
Chagyrskaya 19	0.65	0.60-0.70	16,569	6,348	1,663,955	2
Chagyrskaya 20	0.28	0.22-0.35	16,569	1,792	553,167	0
Chagyrskaya 41	1.7	1.57-1.84	16,569	2,542	842,934	0
Chagyrskaya 60	5.51	5.36-5.68	16,569	4,274	1,020,196	1
Okladnikov 11	6.68	4.82-9.00	16,568	53	18,726	177
Okladnikov 15	10.82	8.05-14.21	16,567	34	10,230	495

SI Table 3.1. We show the amount of contamination, number of sites covered, average coverage, number of reads and Ns in the consensus sequence for all 17 remains. Note that this includes all reads from libraries with around/or less than 20% contamination estimates for remains from Chagyrskaya and only deaminated reads from Okladnikov remains. contamination = present-day human DNA contamination; CI = confidence interval; n = number; N = unresolved/uncalled positions

Heteroplasmies

Heteroplasmies are common in present-day humans, for example Payne et al. identified heteroplasmies with a frequency greater than 2% in all individuals in their study (n=32)[80]. In a larger study around 40% of individuals had at least one heteroplasmy with a frequency higher than 10% (n= 56,434)[81]. Around 20% of mitochondrial heteroplasmies are found in both mothers and their children[82]. The number of shared heteroplasmies are lower between siblings (17.7%) and grandparents-grandchildren (16.4%) and very low for father-children (1.5%)[83]. Thus, we might be able to use heteroplasmies to infer that different individuals represented in our dataset are closely related individuals. We identified four of the five unresolved positions in our consensus sequences as heteroplasmies and we show the four putative heteroplasmic positions in **SI Table 3.2** and discuss them in turn. The last unresolved position (16,181) is a T inserted into a poly-C stretch.

**5 unresolved positions**	**alt**	**ref**	**n**	**Frequency (%) (95% CI)**
**Heteroplasmies (poly C-stretch)**				
**Position = 303-318**				
Chagyrskaya 63	73	21	94	77.7 [69.2-86.1]
Chagyrskaya 13	291	115	406	71.7 [67.3-76.1]
Chagyrskaya 19	467	208	675	69.2 [65.7-72.7]
Chagyrskaya 12	23	28	51	45.1 [31.4-58.8]
**Heteroplasmies (single positions)**				
**Position = 545**				
Chagyrskaya 7	126	134	260	48.5 [42.4-54.6]
Chagyrskaya 9	24	55	79	30.3 [20.1-41.6]
Chagyrskaya 6	3	12	15	20.0 [4.3-48.0]
Chagyrskaya 14	123	341	464	26.5 [22.5-30.5]
**Position = 3,961**				
Chagyrskaya 63	639	206	845	75.6 [72.7-78.5]
Chagyrskaya 13	1,149	573	1,722	66.7 [64.5-68.9]
Chagyrskaya 19	1,741	1,044	2,785	62.5 [60.7-64.3]
**Position = 16,146**				
Chagyrskaya 2	264	1,123	1,387	19 [16.9-21.1]
**Insertion**				
**Position = 16,181**				
Chagyrskaya 7	-	5	5	100
Chagyrskaya 9	-	8	8	100
Chagyrskaya 14	-	16	16	100
Chagyrskaya 6	no data	no data	no data	no data
Chagyrskaya 18	-	24	24	100
Chagyrskaya 20	-	35	35	100
Chagyrskaya 41	-	37	37	100

SI Table 3.2. We show the positions of 4 heteroplasmies and 1 insertion along with the remains that carry the variant.

Position = 303-318

We first set to resolve the poly-C stretch (positions 303-318 in the coordinate system when using *Chagyrskaya 8* as a reference genome), which corresponds to two repetitions of a variable number of Cs separated by a single T. To infer the number of Cs for each remain and avoid erroneous C>T substitutions due to ancient DNA damage, we only looked at sequences that fully overlapped positions 300 to 320 (coordinates according to the Chagyrskaya 8 reference genome) and aligned in the reverse orientation.

For *Chagyrskaya 12*, the number of Cs that show the highest support among all sequences is different from the most supported number of Cs among sequences exhibiting signs of ancient DNA damage (10C1T5C vs. 9C1T5C). In fact, both patterns are almost equally represented among all sequences (28 sequences with 10C1T5C and 23 sequences with 9C1T5C; in contrast to at most 3 sequences for other patterns) and among sequences with signs of ancient DNA damage (5 sequences with 9C1T5C and 4 sequences with 10C1T5C).

We also observed support for both 10C1T5C and 9C1T5C for three other remains (*Chagyrskaya 63*, *Chagyrskaya 13* and *Chagyrskaya 19*) that all represent the same individual, according to our nuclear genome analysis (**SI 7 - Identical individuals**).

For this individual, the proportion of sequences that support these two alleles is different from *Chagyrskaya 12*, but consistent across the three remains (**SI Table 3.2**). No other Chagyrskaya remain showed reasonable support for an alternative allele. However it is worth noting that *Chagyrskaya 1* and *Chagyrskaya 2* have the configuration 8C1T5C (they do not group in the phylogenetic tree), while *Chagyrskaya 1, 6, 9, 14, 17, 18, 20, 41* and *60* have the configuration 7C1T5C. *Chagyrskaya 12, 13, 19* and *63* also do not group in the phylogenetic tree. It would seem this particular mutation has a very high mutation rate as has been previously shown [84].

Because an artefact is extremely unlikely to happen at similar frequencies and only among the remains derived from the same individual (and yet at different frequencies between individuals), the most likely explanation is that the high support for both 9C1T5C and 10C1T5C represent a shared heteroplasmy between *Chagyrskaya 8/12* and *Chagyrskaya 63/13/19.* We edited the number of Cs in the reconstruction of each of the mitochondrial genomes to match the pattern with the highest support, except for these heteroplasmic samples where we chose the pattern 10C1T5C to be consistent with the published sequence of *Chagyrskaya 8*. However, the high level of present-day human DNA contamination in the two Okladnikov remains and the low coverage depth among sequences that exhibit signs of ancient DNA damage did not allow us to resolve this region in the genomes of these two remains.

Position = 545, 3961 and 16146

The next three unresolved positions correspond to single nucleotide differences among the sequences that cover the following positions: position 545 (G/A for *Chagyrskaya 7*, *Chagyrskaya 9, Chagyrskaya 6* and *Chagyrskaya 14*), position 3,961 (C/T for *Chagyrskaya*

63, Chagyrskaya 13 and *Chagyrskaya 19*) and position 16,146 (C/T for *Chagyrskaya 2*) in the coordinates of the *Chagyrskaya 8* reference genome.

As we observe both alleles in both the forward and reverse orientation at all three positions, errors due to ancient DNA damage cannot alone explain these mismatches. Present-day human DNA contamination that would introduce alternative alleles is also an unlikely explanation as the contamination estimates are not particularly high (0.2% - 13.2%) in these remains (**SI Table 3.1**), and the frequencies of both alleles are similar between all sequences and those that exhibit at least one C>T substitution within the first three and/or last three positions (**SI Table 3.3**). In addition, both alleles are present in all libraries of the same remain at similar frequencies (**SI Table 3.4**). Finally, the regions are not in repetitive regions, so we would not expect mapping to be an issue. Thus, we conclude that all three mismatches appear to be genuine heteroplasmies.

Position	remain	sequences	Depth of coverage	% of alternative allele
545<br>Reference: A<br>Alternative: G	Chagyrskaya 7	all	260	48.5
		deaminated	136	50.7
	Chagyrskaya 9	all	79	30.3
		deaminated	38	31.5
	Chagyrskaya 14	all	464	26.5
		deaminated	242	25.2
	Chagyrskaya 6	all	15	20
		deaminated	10	20
3,961<br>Reference: C<br>Alternative: T	Chagyrskaya 63	all	845	75.6
		deaminated	158	74.7
	Chagyrskaya 13	all	1,722	66.7
		deaminated	249	61.8
	Chagyrskaya 19	all	2,785	62.5
		deaminated	446	59.6
16,146<br>Reference: T<br>Alternative: C	Chagyrskaya 2	all	1,387	19.0
		deaminated	88	23.9

SI Table 3.3. Support for both alleles of three single nucleotides in heteroplasmic remains. We focused on sequences in the orientation that allow unambiguous identification of the base, avoiding ancient DNA damage, i.e. reverse for the C/T polymorphisms and forward for the G/A polymorphism. We used the mitochondrial genome of Chagyrskaya 8 as a reference.

Position	remain	Library ID	Depth of coverage	% of alternative allele
545<br>Reference: A<br>Alternative: G	Chagyrskaya 7	G8326	56	42.8
		G8327	86	52.3
		G8328	58	43.1
		R5788	23	57.6
		R9878	27	48.2
	Chagyrskaya 14	A25735	124	29.0
		F3118	199	26.1
		F8792	141	24.8
	Chagyrskaya 6	A25726	9	33.3
		R5790	2	50.0
		R9876	8	12.5
	Chagyrskaya 9	L5123	41	31.7
		R1919	43	30.2
		R1920	51	35.2
		R1921	67	28.4
		L5258	41	31.7
		L5259	41	31.7
		L5260	41	31.7
		L5261	41	31.7
3,961<br>Reference: C<br>Alternative: T	Chagyrskaya 63	A24563	171	74.9
		A24564	228	74.1
		A24565	176	76.7
		A25747	20	85.0
		F3260	158	81.7
		F8803	92	66.3
	Chagyrskaya 13	A25732	1,236	67.6
		F3117	217	65.9
		F8791	269	63.2
	Chagyrskaya 19	A25740	2,377	62.1
		F3251	98	55.1
		F8795	310	68.1
16,146<br>Reference: T<br>Alternative: C	Chagyrskaya 2	A25718	1,331	18.1
		R5789	30	50.0
		R9875	26	26.9

SI Table 3.4. Support for both alleles of the three single nucleotides for each library of the heteroplasmic remains. We focused on sequences in the orientation that allow unambiguous identification of the base, avoiding ancient DNA damage, i.e. reverse for the C/T polymorphisms and forward for the G/A polymorphism. We used the mitochondrial genome of *Chagyrskaya 8* as a reference and show the results for all sequences.

Position = 16,181

The last unresolved region is another poly-C stretch starting at position 16,181. Unfortunately, there are very few Cs in the reverse orientation in that region so that few sequences in the reverse orientation span the complete poly-C stretch and have at least one C>T substitution within the three first or last positions. Among all sequences in the reverse orientation that overlap the stretch (including those without C>T substitutions; from position 16,179 to 16,194), it was not possible to determine the length of the repeat for all individuals, which may be 10, 11 or 12 Cs long (**SI Table 3.5**). Contamination is unlikely to produce such high support for the alternative alleles; instead, these alternative alleles most likely stem from errors introduced by the DNA polymerase when that poly-C stretch is long. Indeed, there were few errors for the remains with a poly-C stretch interrupted by a T (*Chagyrskaya 7, 9, 14, 18, 20* and *41;* **SI Table 3.5**). For the other remains, we masked the stretch in the mitochondrial genome reconstruction using 12 Ns, which is the length of that stretch for most published Neanderthal mitochondrial genomes.

remain	dataset	# sequences	pattern
Chagyrskaya 1	All sequences	23	10C
		26	12C
		41	11C
	deaminated sequences	4	11C
		6	12C
Chagyrskaya 2	All sequences	90	10C
		133	11C
		145	12C
	deaminated sequences	4	10C
		5	11C
		14	12C
Chagyrskaya 6	All sequences	2	6C1T4C
	deaminated sequences	NA	NA
Chagyrskaya 7	All sequences	**79**	**6C1T4C**
	deaminated sequences	**5**	**6C1T4C**
Chagyrskaya 9	All sequences	2	5C1T4C
		3	11C
		**53**	**6C1T4C**
	deaminated sequences	**8**	**6C1T4C**
Chagyrskaya 63	All sequences	10	12C
		11	11C
		12	10C
	deaminated sequences	3	10C
Chagyrskaya 12	All sequences	8	10C
		10	11C
		14	12C
	deaminated sequences	2	10C
		3	12C
Chagyrskaya 13	All sequences	25	10C
		37	12C
		42	11C
	deaminated sequences	2	12C

		2	3C1A8C
		6	10C
Chagyrskaya 14	All sequences	3	5C1T4C
		**75**	**6C1T4C**
	deaminated sequences	**16**	**6C1T4C**
Chagyrskaya 17	All sequences	4	5C1T4C
		9	11C
		15	12C
	deaminated sequences	2	12C
		4	11C
Chagyrskaya 18	All sequences	3	5C1T4C
		**236**	**6C1T4C**
	deaminated sequences	**24**	**6C1T4C**
Chagyrskaya 19	All sequences	52	12C
		56	10C
		67	11C
	deaminated sequences	7	10C
		7	12C
		8	11C
Chagyrskaya 20	All sequences	2	5C1T4C
		**219**	**6C1T4C**
	deaminated sequences	**35**	**6C1T4C**
Chagyrskaya 41	All sequences	8	5C1T4C
		**187**	**6C1T4C**
	deaminated sequences	2	5C1T4C
		**37**	**6C1T4C**
Chagyrskaya 60	All sequences	66	10C
		75	12C
		85	11C
	deaminated sequences	4	12C
		5	10C
		5	11C
Okladnikov 11	All sequences	18	6C1T4C
		28	11C
		28	12C
	deaminated sequences	3	11C
Okladnikov 15	All sequences	2	12C
		3	11C
		13	5C1T4C
	deaminated sequences	NA	NA

SI Table 3.5. Support for the three most represented alleles among sequences that align to the poly-C stretch at position 16,181. We focused on sequences that align to the mitochondrial genome of *Chagyrskaya 8* in the reverse orientation, to avoid C>T substitutions due to ancient DNA damage, and that cover at least positions 16,179 to 16,194. We report the number of sequences with an allele observed at least twice among the sequences of a remain. The grey shading indicates the subset of sequences with evidence of at least one substitution due to ancient DNA damage within the three first or last

positions. The alleles marked in bold are those present in the mitochondrial genome reconstruction. For the other remains, we masked the complete stretch with 12 Ns.

Phylogenetic relationship

We made a multiple sequence alignment of the reconstructed mtDNA genomes from our 17 remains along with previously published mitochondrial sequences as detailed below.

While molecular dating has already been performed on the *Chagyryskaya 8* remains, we wanted to compare the divergence times of different mitochondrial (mtDNA) genomes of all individuals from the Chagyrskaya and Okladnikov Caves.
The updated calibration curve IntCal20[85] also necessitates updating the molecular dating of archaic hominins.
In order to do this we aligned mtDNA genomes of 54 present-day[67] and 10 ancient modern humans[63,66,86–89], 42 Neanderthals (i.e., the 17 mtDNA genomes newly reconstructed here; 24 previously-published mtDNA genomes[2,51,67–77]; and one sequence reconstructed from a sediment sample from layer 6c in Chagyryskaya Cave [5]., 4 Denisovans [87,90–92], one Sima de los Huesos individual[93] and a chimpanzee[94] to the revised Cambridge reference sequence (rCRS)[65] using MAFFT[95].
We performed the following analysis on both the full mtDNA genome and just the mtDNA coding region (577-16,023 based on rCRS) due to previous concerns about bias from including the variable D-loop region[74].

We then used BEAST2[96] to construct a phylogenetic tree and estimate divergence times. We used a path sampling approach from the MODEL_SELECTION[97] package in BEAST2 to identify the best fitting clock and tree model for the analysis.

For this test we used 40 path steps, a chain length of 25,000,000 iterations, a parameter alpha of 0.3, a pre-burn-in of 75,000 iterations and an 80% burn-in of the whole chain. For the full mtDNA genome a mutation rate of 2.53E-08 mutations per base pair per year was used[86] and for the analysis restricted to the coding mtDNA genome a mutation rate of 1.57E-08 mutations per base pair per year was used.

As in previous analyses, a normal distribution was used with the mean set to the relevant mutation rate with a sigma of 1.00E-10[63] for both the relaxed log normal and strict clock models. The substitution model Tamura-Nei 1993 (TN93)[98] was used for all models as this was previously estimated to be the best model for archaic hominins.

The tree was calibrated with updated INTCAL20 calibrated radiocarbon dates from previously published radiocarbon dates of ancient modern humans and Neanderthals (**SI Table 3.6**). For individuals with no upper limit (i.e. El Sidrón 1253) an upper limit of 70,000 was set.

Individual	Radiocarbon date ID	Raw radiocarbon date	Publication of the date	Calibrated date with IntCal20 and OxCal 4.4.2, cal BP 95.4% (round to 10)
Modern humans (n=10)				
Ust'Ishim	OxA-25516 & 30190	41,400±950	Fu et al, 2014	45,930-42,900
Tianyuan	BA-03222	34,430±510	Fu et al, 2013	40,850-38,070
Kostenki14	OxA-X-2395-15	33,250±500	Marom et al, 2012	39,380-36,670
Vestonice13	Aix12027	27,040±100	Fewlas et al, 2019	31,240-31,030
Vestonice14	Aix12028	26,760±100	Fewlas et al, 2019	31,150-30,850
Loschbour	OxA-7338	7,205±50	Toussaint et al, 2007	8,170-7,930
Iceman	Oxa37371-6 and OxA-3419-21	4,540+-55	Bonana et al 1992, Bonana et al 1994, Hedges et al 1992	5,450-4,970
Eskimo Saqqaq	OxA20656	4,044 ± 31	Rasmussen et al 2010	4,790-4,410
Oberkassel	OxA-4790	11,570±100	Fu et al, 2013	13,730-13,180
Boshan	MAMS-13530	7,368±34	Fu et al, 2013	8,320-8,030
Neanderthals (n=28)				
ElSidrón 1253	OxA-21776	48,400 ± 3,200	Wood et al, 2012	>50,000 - 46,970
Feldhofer 1	ETH-20981	39,900 ± 620	Schmitz et al, 2002	44,290-42,530
Feldhofer 2	ETH-19660	39,240 ± 670	Schmitz et al, 2002	44,040-42,250
Fonds-de-Forêt	OxA-X-2767-13, OxA-38322	38,800 ± 900, 39,500 ± 1,100	Deviese et al, 2021	44,220-41,900; 44,860-42,070
Goyet Q305-4	GrA-46176	40,690 +480, −400	Rougier et al, 2016	44,490-42,980
Goyet Q56-1	GrA-46170	38,440 +340, −300	Rougier et al, 2016	42,740-42,160
Goyet Q57-1	GrA-46173	41,200 +500, −410	Rougier et al, 2016	44,880-43,160
Goyet Q57-2	GrA-54024	36,590 +300, −270	Rougier et al, 2016	42,000-41,100
Goyet Q57-3	GrA-60019	38,260 +350, −310	Rougier et al, 2016	42,670-42,080
Les Cottés Z4-1514	MAMS-26196	39,485 ± 271	Hajdinjak et al, 2018	43,150-42,540
Mezmaiskaya 2	OxA-21839	39,700 ± 1,100	Pinhasi et al, 2011	44,970-42,150
Spy94a	GrA-32623; OxA-X-2762-21	35,810 +260, -240; 41,500 ± 1,800	Semal et al, 2009; Deviese et al, 2021	41,360-40,460; 49,740-42,330
Vindija 33.16 (previously Vi-80)	? [Uppsala]	38,310 ± 2,130	Serre et al, 2004	49,130-39,380
Vindija 33.19	OxA-X-2717-11	44,300 ± 1,200	Deviese et al, 2017	49,930-44,690
Vindija 33.25				
Vindija 33.17				
Altai Nea/Denisova 5				
Denisova 11				
Denisova 15				
Goyet Q305-7				
Goyet Q374a-1				
Hohlenstein Stadel Neanderthal (HST)				
Mezmaiskaya 1				
Okladnikov 2				
Scladina I-4A				
Sima de los Huesos				
Chagyrskaya 8				
sediment layer 6c				

SI Table 3.6. We show 24 previously published ancient remains with raw and calibrated carbon dates used for calibrating the BEAST tree.
For remains that had dates with non-symmetrical confidence intervals (e.g. +480, -400), the larger value was used for calibration. For remains where multiple radiocarbon dates were available the most recent date and/or the date with the largest confidence intervals was also used (in bold) for the BEAST tree calibration.

For samples without radiocarbon dates, dates were restricted as described in previous studies[73]: Modern samples were set to 0, Neanderthals of unknown age to a range of 30,000 to 200,000 years (except for Sima de los Huesos which was constrained to between 200,000 and 780,000 years), *Denisova 3* to 30,000 to 100,000, and the three other Denisovans (*Denisova 2*, *4*, and *8*) to 30,000 to 300,000 years. A uniform prior over the allowed range of dates was used for each individual. The Neanderthals, modern humans, and Denisovans with the Sima de los Huesos individual were constrained to monophyletic groups, enabling us to estimate the time to their most recent common ancestor (TMRCA).
For the full mtDNA genome a model with a relaxed log normal clock and a tree with a Bayesian skyline population was significantly better than all other models (Bayes Factors >5), except for a model with a strict clock and Bayesian skyline population (see **SI Table 3.7** for marginal likelihood estimates). As there was no significant difference between these two models, the latter was selected as it is the simpler model. For the coding region of the mtDNA genome the model with a relaxed log normal clock and a tree with a Bayesian skyline population was only significantly better than a model with a strict clock and a constant population, while all other models were not significantly different (**SI Table 3.7**). Therefore, the model with a relaxed log normal cloak and a tree with a Bayesian skyline population was selected as the simplest method that was not significantly worse than any other model.

Target	Clock Model	Tree Model	Marginal log likelihood
Full mtDNA genome	**Strict**	Constant	-41,632.48
		**Bayesian skyline**	**-41,614.08**
	Relaxed log normal	Constant	-41,620.24
		Bayesian skyline	-41,600.33
Coding	**Strict**	Constant	-35,723.22
		**Bayesian skyline**	**-35,716.27**
	Relaxed log normal	Constant	-35,715.80
		Bayesian skyline	-35,705.42

SI Table 3.7. We show the marginal log likelihood for different models. The best fitting models are highlighted.

Using this model, three Markov chain Monte Carlo (MCMC) runs of 75,000,000 iterations (10,000,000 pre-burn iterations) and sampling every 2,000 trees were completed. Logcombiner2 from BEAST2 was used to combine the log and tree files from these runs. A summary of a single tree was identified by using the tree annotator program from BEAST2 on the combined tree file. The programs Tracer (version 1.7)[99] and Figtree (version v1.4.4)(https://github.com/rambaut/figtree) and BEAST2[96] were used to examine the resulting tip dates and TMRCA estimates (**SI Table 3.8** and **SI Figure 3.2**).

Parameter	Target	Mean tip date (kya)	95% HPD Interval (kya)	ESS
**Chagyrskaya and Okladnikov remains**				
Chagyrskaya sediment layer 6c	Coding	86	59-111	136
	Full mtDNA	85	62-106	99
Chagyrskaya 8	Coding	74	51-99	121
	Full mtDNA	77	56-97	91
Chagyrskaya A 1	Coding	80	57-104	115
	Full mtDNA	81	60-100	88
Chagyrskaya B 2	Coding	72	49-96	118
	Full mtDNA	73	51-94	95
Chagyrskaya C 14	Coding	89	66-114	133
	Full mtDNA	83	59-103	96
Chagyrskaya D 7	Coding	88	66-115	134
	Full mtDNA	83	60-104	96
Chagyrskaya E 9	Coding	88	65-114	135
	Full mtDNA	83	59-103	96
Chagyrskaya F 12	Coding	74	51-99	122
	Full mtDNA	77	55-97	91
Chagyrskaya G 19	Coding	72	49-96	118
	Full mtDNA	75	54-95	92
Chagyrskaya H 17	Coding	66	44-91	120
	Full mtDNA	71	50-93	94
Chagyrskaya I 18	Coding	88	66-116	132
	Full mtDNA	83	60-103	96
Chagyrskaya J 20	Coding	88	63-113	132
	Full mtDNA	83	59-103	96
Chagyrskaya K 41	Coding	89	65-115	127
	Full mtDNA	83	59-104	96
Chagyrskaya L 60	Coding	66	44-91	120
	Full mtDNA	71	50-93	94
Okladnikov 2	Coding	92	69-116	235
	Full mtDNA	78	60-95	190
Okladnikov A 11	Coding	92	69-116	236
	Full mtDNA	75	56-93	206
Okladnikov B 15	Coding	72	49-96	120
	Full mtDNA	70	48-92	98
**Denisova Cave Neanderthals**				
Altai	Coding	136	102-173	528
	Full mtDNA	118	91-144	292
Denisova11	Coding	101	80-122	290
	Full mtDNA	92	77-108	125
Denisova15	Coding	132	97-167	490
	Full mtDNA	116	89-142	298
**Denisovans**				
Denisova2	Coding	186	134-238	1,187

	Full mtDNA	163	123-203	385
Denisova3	Coding	89	68-100	599
	Full mtDNA	86	66-100	254
Denisova4	Coding	94	65-114	257
	Full mtDNA	91	66-110	177
Denisova8	Coding	155	101-207	1,295
	Full mtDNA	139	97-180	389
**Late European Neanderthals**				
ElSidrón 1253	Coding	57	47-68	8,736
	Full mtDNA	54	47-61	1,376
GoyetQ305-7	Coding	42	33-47	227
	Full mtDNA	42	36-47	180
GoyetQ374a-1	Coding	42	34-47	222
	Full mtDNA	42	36-47	179
Vindija33.17	Coding	52	43-59	212
	Full mtDNA	50	44-55	179
Vindija33.25	Coding	45	35-55	228
	Full mtDNA	45	38-51	176
**Early European Neanderthals**				
HST	Coding	137	90-189	1,147
	Full mtDNA	111	79-147	682
Mezmaiskaya1	Coding	111	80-144	504
	Full mtDNA	103	77-128	214
Scladina I-4A	Coding	126	91-162	528
	Full mtDNA	109	83-137	282
Sima de los Huesos	Coding	324	223-430	400
	Full mtDNA	278	203-345	209
**TMRCAs**				
Humans	Coding	142	116-169	13,802
	Full mtDNA	124	106-143	12,710
Neanderthals	Coding	280	240-322	1,007
	Full mtDNA	222	194-250	386
Humans-Neanderthals	Coding	428	381-478	2,342
	Full mtDNA	328	296-361	637
Denisovans	Coding	248	202-293	838
	Full mtDNA	211	175-248	272

SI Table 3.8. We show the mean tip dates for different remains along with their 95% highest posterior density intervals (HPD).

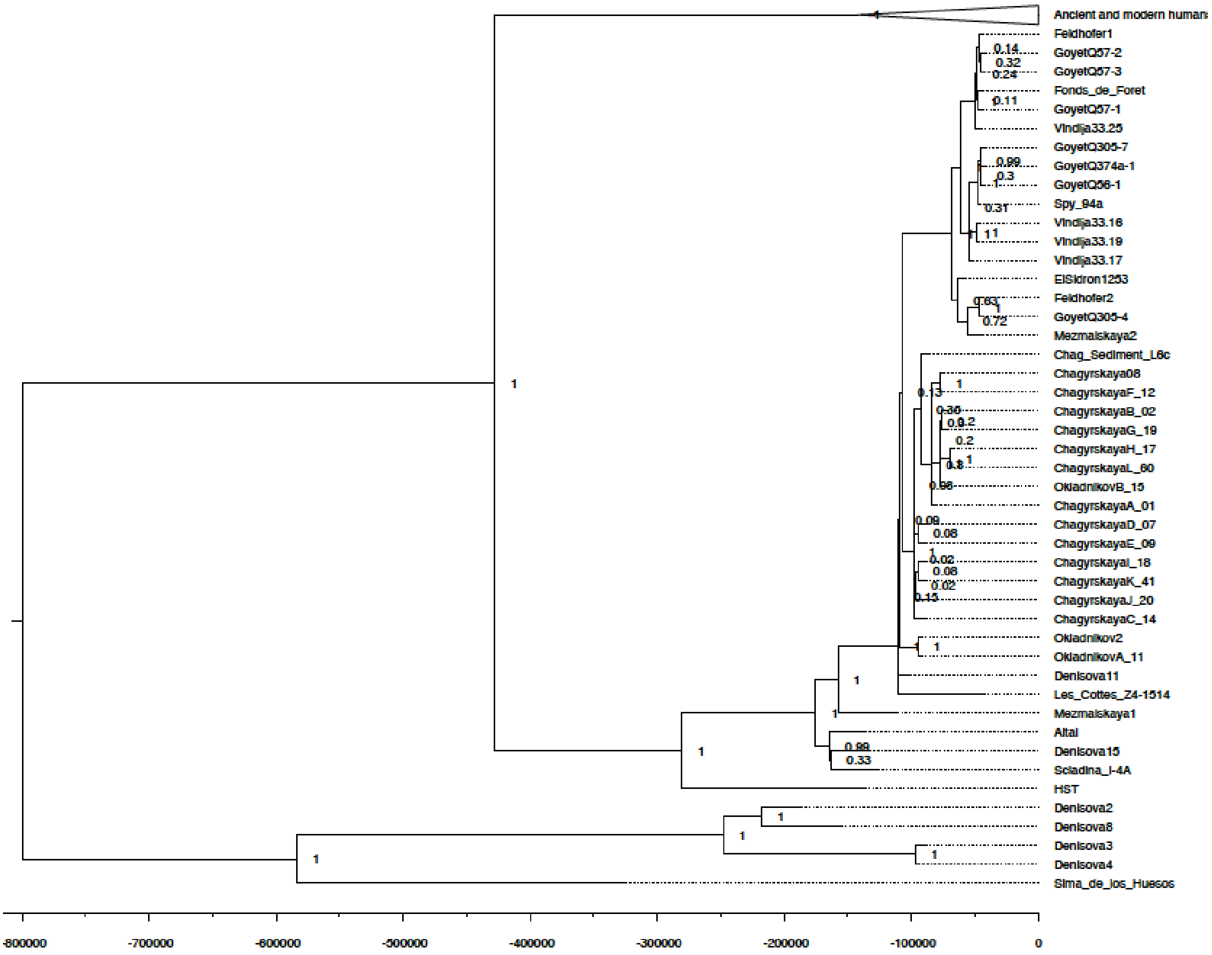

SI Figure 3.2 BEAST2 tree of the mtDNA coding region for Neanderthals, Denisovans and a selection of ancient modern humans

A chimpanzee mtDNA genome was used to root the tree, but is not pictured here. The posterior for each branch point is shown.

We find that the Chagyrskayas remains are dated to between 46 and 125 thousand years ago (95% HPD) based on their mitochondrial DNA genomes.

We show all the variable positions between the Chagyrskaya and Okladnikov samples in **SI Table 3.9**.

Ind	Specimen	Sex	150	151	184	721	4739	5451	6296	10747	11017	13142	14293	15647	16090	16187	16261	16273	16462
H	Chagyrskaya 17	F	t	t	a	a	c	t	g	a	g	a	g	g	t	c	t	g	a
L	Chagyrskaya 60	F	t	t	a	a	c	t	g	a	g	a	g	g	t	c	t	g	a
B	Chagyrskaya 02	M	t	t	a	a	c	t	g	a	g	g	g	g	t	c	t	g	g
	Chagyrskaya 13	M	t	t	a	a	c	t	g	a	g	g	g	g	t	c	t	g	a
	Chagyrskaya 19	M	t	t	a	a	c	t	g	a	g	g	g	g	t	c	t	g	a
G	Chagyrskaya 63	M	t	t	a	a	c	t	g	a	g	g	g	g	t	c	t	g	a
B	Okladnikov 15	F	n	t	n	a	c	t	g	a	g	g	g	g	t	n	t	g	n
A	Chagyrskaya 01	M	t	t	a	a	c	t	g	a	a	g	g	g	t	c	t	g	a
F	Chagyrskaya 12	F	t	t	a	a	c	t	g	a	a	g	g	a	t	c	t	g	a
D	Chagyrskaya 07	M	t	t	a	a	c	t	a	g	a	g	g	g	t	t	t	a	a
E	Chagyrskaya 09	M	t	t	a	a	c	t	a	g	a	g	g	g	t	t	t	a	a
	Chagyrskaya 14	M	t	t	a	a	c	t	a	g	a	g	g	g	t	t	t	a	a
C	Chagyrskaya 06	M	n	t	n	a	c	t	a	n	a	g	g	n	t	t	n	n	a
I	Chagyrskaya 18	F	t	t	a	a	c	t	a	g	a	g	g	g	t	t	t	a	a
J	Chagyrskaya 20	F	t	t	a	a	c	t	a	g	a	g	g	g	t	t	t	a	a
K	Chagyrskaya 41	M	t	t	a	a	c	t	a	g	a	g	g	g	t	t	t	a	a
A	Okladnikov 11	M	c	t	g	g	t	c	a	g	a	g	a	g	c	n	c	g	a
	Okladnikov 2	M	c	c	a	g	t	c	a	g	a	g	a	g	c	n	n	g	a

SI Table 3.9. List of all varying positions (D-loop region aka hyper variable regions are marked in red) for the Chagyrskaya and Okladnikov remains. Positions are relative to the alignment. Derived alleles are colored in orange and missing alleles are colored in gray.

TMRCA for mitochondrial sequences

We wanted to calculate the time to the most recent common ancestor for the mitochondrial sequences using equation (7), (8) and (9) from a previous study[100]. In essence we can obtain a probability density function for the coalescence time given that we observe k differences between two sequences. The probability density function is:

$$f_{coaltime}(t|k) = \frac{(1+\theta)^{1+k}}{k!} t^{k} e^{-(1+\theta)t}$$

We set N=100 individuals, mu = 1.57e-8 mutation per base pairs per year, generation time to 29 years[101], length of the mitochondrial sequence (L) to 15,446 base pairs, θ to 2 * N * mu * L.

**Differences (k)**	**Mean coal time (years)**	**Min 95% CI coal time (years)**	**Max 95% CI coal time (years)**
0	1205	31	3617
1	2417	295	5734
2	3630	739	7644
3	4849	1329	9382
4	6053	1971	11085

SI Table 3.10. The mean coalescence time in years along with the 95% confidence intervals between two mitochondrial sequences with k differences.

Note that **SI Table 3.10** shows the mean coalescence time. If we have two sequences that have 0 differences between them that means that the total branch length separating them is 2 * 1205 - this branch point includes the common ancestor. Thus the mean time that could have passed from the ancestor to both individuals is 2 * 1205 = 2410 years.

SI 4 Y chromosome DNA capture and sequencing

Array design

We enriched an aliquot of each amplified library from fossils identified as male for ~6.9 Mb of Y chromosome regions as described previously[10]. Briefly, we targeted non-repetitive regions of the modern human Y chromosome where 50% of 35 k-mers can be mapped uniquely, and removed regions that were shorter than 99 contiguous base pairs (bp). A total of 2,049,846 52-bp probes were designed based on the modern human reference (hg19) using a 3-bp tiling[10].

The captured regions span both the X-degenerate, ampliconic, X-transposed and heterochromatic regions and regions marked as 'other'[102]. 76% of sites of the array target the X-degenerate regions. **SI Table 4.1**.

Regions classes	Length of region in hg19 (bp)	Overlapping bases of array and region (bp)	Proportion of bases on array that overlap with region class (%)
Pseudo-autosomal	2,978,885	0	0.00%
X-degenerate	8,418,392	5,327,700	76.89%
X-transposed	3,400,750	38,300	0.55%
Ampliconic	9,940,791	1,467,755	21.18%
Others	683,506	54,097	0.78%
Heterochromatic	3,1795,067	24,869	0.36%

SI Table 4.1. Intersection of the Y chromosome capture array and region classes on the human reference Y chromosome. The total number of sites captured in the array is 6,928,959.

Coding genes and transcription factors on the Y chromosome array

We downloaded a list of known genes and transcription factors that overlap with the array from UCSC table browser (https://genome.ucsc.edu/cgi-bin/hgTables)[103]. We selected **assembly:** hg19, **track:** UCSC genes, **table:** knowngene

We found 15 single copy genes, 8 single copy transcription units, 3 pseudogenes and 2 non-coding RNAs.

Additional Y chromosomes used

In addition to the 11 male fossils from Chagyrskaya and Okladnikov we also used Y chromosome sequences from:

4 modern humans

- hg19 reference genome
- S_Han-2 and S_French-1 from SGDP[104]
- Merged data from two haplogroup A00 individuals[105,106].

2 Denisovans and 3 Late Neanderthals[10]

- *Denisova 4* (1.4X coverage)
- *Denisova 8* (3.5X coverage)
- *Mezmaiskaya 2* (14.3X coverage)
- *Spy 94a* (0.8X coverage)
- *El Sidrón 1253* (7.9X coverage but only of a 560 kb region)

Captured libraries

We captured 30 libraries from the 11 male Chagyrskaya and Okladnikov remains following the same protocol as in[10] - capture stats are in **SI Dataset1 - Y chrom capture tab**. In addition to Y chromosome capture data we also use all shotgun sequences that map to the Y chromosome for *Chagyrskaya 7*.

We filtered all BAM files for reads with a mapping quality greater than 25 (MQ>25) and a read length greater than 35 (L>35). In addition to this we required all reads to map to the targeted regions of the ~6.9 Mb of the Y chromosome capture array.

The number of Y DNA sequences generated from each library and the frequency of their C>T substitutions at the 5’ and 3’ ends are presented in **SIdataset1 - Y chrom capture tab**. The elevated C>T substitutions provide evidence for the presence of Y sequences of ancient origin in most of the libraries prepared from the Chagyrskaya and Okladnikov remains. We do not observe strong ancient DNA damage patterns for the negative control libraries (a maximum of 37 reads with a C>T or G>A substitution in the the first/last 3 positions of the read in a single library (**SIDataset1 - Y chrom capture tab**))

The coverage of the captured Y chromosome regions per individual ranges from 0X to 42X - see **SI Dataset1 - Y chrom capture tab**.

Modern human contamination estimates

To estimate levels of present-day human DNA contamination in the Y chromosome capture data we defined sites that are shared by all modern humans, but are not seen in any of the archaic humans or the Chimpanzee (panTro4), along with the sites which vary among modern humans. For this purpose, we use sites in the X-degenerate region that fall on branches of modern human Y haplogroups A1, A1b, BT, CT and F (using the nomenclature of the ISOGG database) from ISOGG (http://www.isogg.org/tree/index.html) and other

studies[107].

We also identify sites that are derived in all modern humans. For this purpose, we use four modern human Y chromosomes; the hg19 reference genome, S_Han-2 and S_French-1 and an individual from the most divergent modern human haplogroup A00. We require that the same allele is found in all four individuals while only a different allele is present and non-missing in at least one Denisovan, one Neanderthal (using 2 Denisovans and 3 Late Neanderthals from [10] and considering an allele called if it is supported by minimum 3 reads and 90% of the reads carry the same allele) and the Chimpanzee Y chromosome (panTro4 mapped to hg19). The allele present in all modern humans is set to the derived allele. We show the number of sites for each branch (note these are not haplogroups but branches) in **SI Table 4.2**. For a complete list of sites used see **SI Dataset2**.

**Branch**	**Sites**
All modern humans	206
A1	8
A1b	20
BT	181
CT	135
F	54
Total	604

SI Table 4.2. For each branch we show the number of sites where either four modern humans (A00, S_Han-2, S_French-1 and the hg19 ref, first row) or three modern humans (S_Han-2, S_French and the hg19 ref, other rows) carry the derived allele.

Our contamination estimate will correspond to the number of reads carrying the derived alleles which overlap any of the 604 sites, divided by the total number of reads overlapping the 604 sites. To calculate 95% confidence intervals for our contamination estimates we use the Wilson score interval, which is based on a binomial distribution[108,109]. In contrast to a normal approximation, this ensures that confidence limits for our contamination rates stay between 0% and 100%.

Testing contamination estimates

To test our contamination estimator we simulated reads from a Neanderthal Y chromosome, *i.e.* we create a set of simulated reads where wea modify the human reference Y chromosome by ancestralizing the 604 sites which are derived in modern humans and adding 674 sites which are derived in two sequenced Neanderthal Y chromosomes[10]. Apart from these 1,278 sites, the simulated Neanderthal Y chromosome and the human reference Y chromosome are identical.

We then used *gargammel*[110] to simulate reads with a length distribution and deamination

profile that mimics those seen in the *Chagyrskaya 41* libraries (an arbitrarily selected fossil for preservation at Chagyrskaya cave). Then we mapped these reads to the human reference Y chromosome using bwa[60] with ancient parameters (bwa aln {refgenome} {fastq} -n 0.01 -o 2 -l 16500).

To determine if our contamination estimates are robust to ancient DNA damage we simulated reads from the ~6.9 Mb regions on the Neanderthal Y chromosome to a coverage of 1X with 1% contamination and assessed whether the contamination estimates are consistent across mutation types (for instance C>G, A>T and so on). We do this because ancient DNA damage results in C>T changes on the forward strand and G>A changes on the reverse strand in single stranded libraries[54,111]. If the derived allele is a T and the ancestral base is a C then we would mistake deamination for contamination.

Indeed, we see that the C>T and G>A sites are estimated to have a higher contamination than all other sites (where 1% of the simulated reads are contaminant). Since using these sites would lead to an overestimate of the amount of contamination we removed them from all further contamination analyses leaving us with 388 sites (**SI Figure 4.1)**.

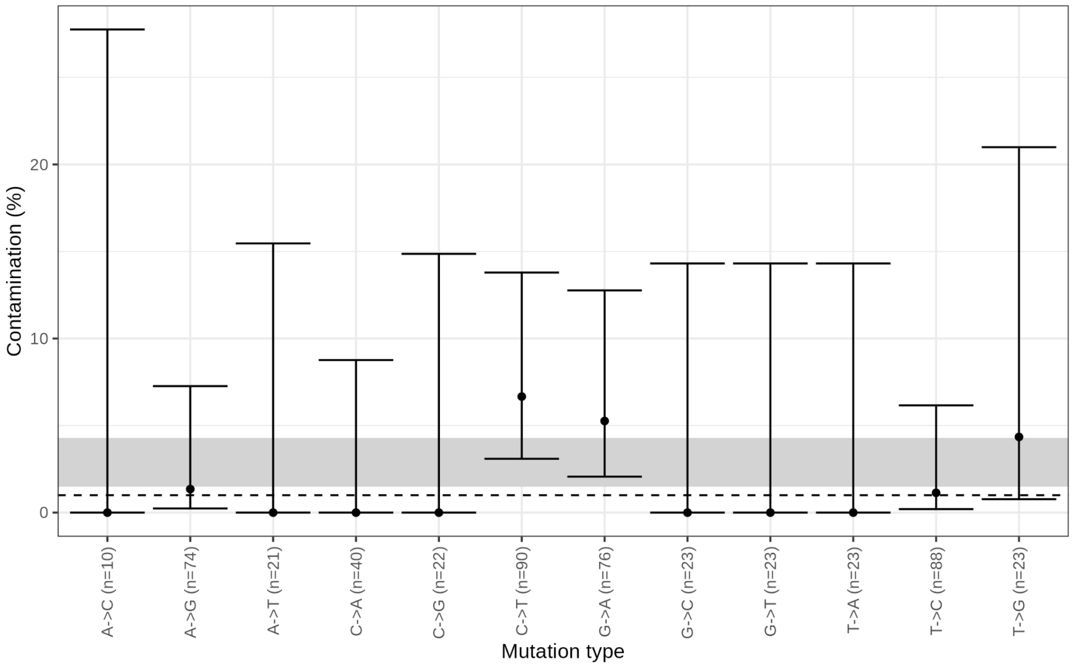

SI Figure 4.1. Contamination estimates for different mutation types. For each mutation type the mean contamination estimate is marked with a dot and the error bars are 95% confidence intervals. n is the number of reads which overlap informative sites. The horizontal dashed line indicates the actual simulated contamination (1%). The grey box is the contamination estimate when all sites are used.

Next we tested how accurate our contamination estimator was by simulating reads from the ~6.9 Mb regions of the Neanderthal Y chromosome to a coverage of 1X and adding contamination (the human reference Y chromosome) ranging from 0-100%. The most contaminated read set is thus 100% reads generated from the human reference Y chromosome.

For this simple test there is a strong correlation between the simulated contamination and

estimated contamination (r2 = 0.9951) - see **SI Figure 4.2**.

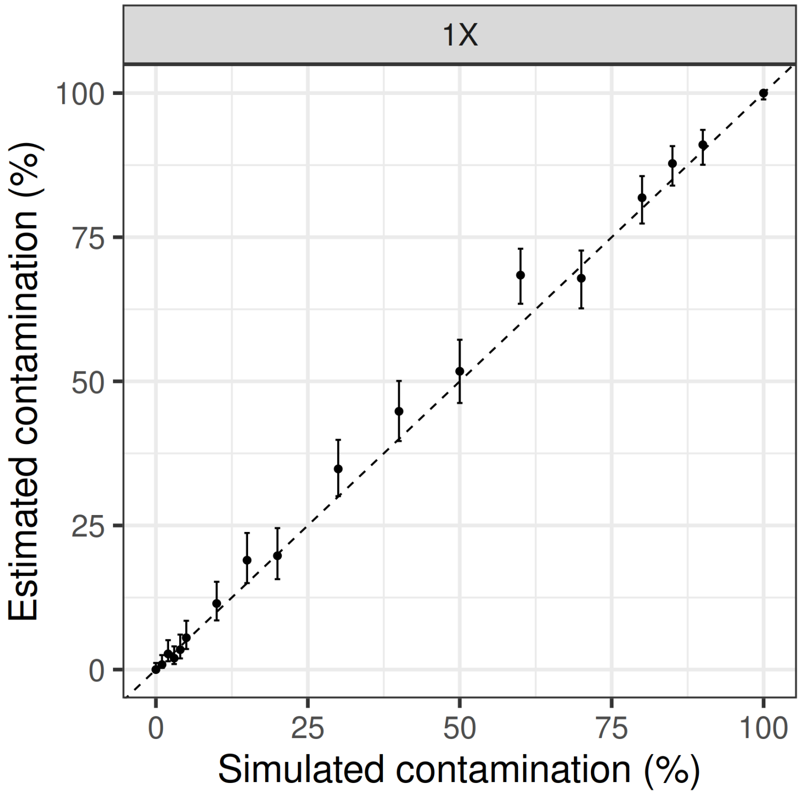

SI Figure 4.2. The center of the error bar is the estimated contamination as a function of simulated contamination. We do not use C>T or G>A differences between modern humans and archaics+chimpanzee for our estimates, so we have n=388 sites for estimating contamination for each data point. The error bars represent 95% confidence intervals using those n=388 sites.

Finally, we investigated how coverage might affect our contamination estimates. We simulated sets of reads yielding between 0.001X and 10X coverage of the Y chromosome, adding 1% contamination to each set as before. We see that as coverage increases we are able to narrow our confidence intervals but we also observe that the true contamination rate (1%) is always within our confidence intervals - see **SI Figure 4.3**.

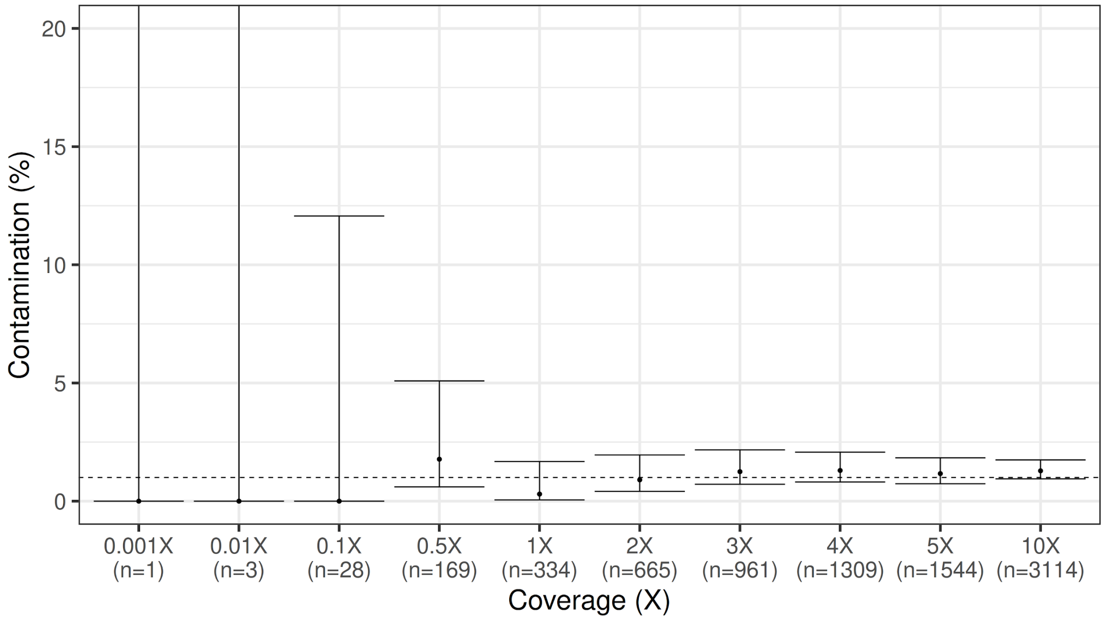

SI Figure 4.3. Contamination estimate as a function of coverage on the Y chromosome. We do not use C>T or G>A mutations for our estimates. As the coverage increases our contamination estimates become more accurate. The point represents the mean contamination estimate and the error bars represent 95% confidence intervals where n is the number of reads overlapping the 388 informative positions (shown below each coverage). The dotted horizontal line shows the simulated contamination of 1%.

Chagyrskaya and Okladnikov contamination estimates

We estimated contamination in the 40 libraries from our 11 male remains both for deaminated reads and for all reads - see **SI Dataset1** using 388 sites for contamination. We then either used all reads for downstream analyses if the contamination estimate was less than 3%, or only deaminated reads if the contamination estimate in all reads was greater than 3% and the contamination estimate for deaminated reads only is less than 3%. If both the deaminated reads and all reads show a contamination estimate bigger than 3% we exclude that library from our downstream analyses.

Curated data set

We merged all libraries originating from the same remain **(SI 7)** based on their contamination estimates (**SI Table 4.3**). We also removed sequence reads which have insertions or deletions from all downstream analyses.

**Individual**	**remain(s)**	**Con-tamination**	**Contamination 95 % CI**	**Sites covered**	**depth of coverage**	**number of reads**
Chagyrskaya A	Chagyrskaya 1	1.1	0.19-5.96	1,692,340	0.3	39,076
Chagyrskaya B	Chagyrskaya 2	0.07	0.04-0.13	6,711,243	42.19	4,071,341
Chagyrskaya C	Chagyrskaya 6	0	0.0-8.38	506,861	0.08	8,899
	Chagyrskaya 14	0	0.0-35.43	151,460	0.02	3,213
	Chagyrskaya 6_14	0	0.0-7.27	645,415	0.11	12,112
Chagyrskaya D	Chagyrskaya 7	0.2	0.05-0.72	6,312,975	2.85	357,235
Chagyrskaya E	Chagyrskaya 9	0	0.0-17.59	370,830	0.06	7,825
Chagyrskaya G	Chagyrskaya 13	1.48	0.64-3.43	4,219,294	1.2	152,419
	Chagyrskaya 19	1.34	0.37-4.76	2,513,399	0.51	67,018
	Chagyrskaya 63	0	0.0-14.87	285,610	0.04	5,195
	Chagyrskaya 13_19_63	1.38	0.67-2.82	4,962,496	1.75	224,572
Chagyrskaya K	Chagyrskaya 41	1.19	0.77-1.83	6,173,098	6.52	953,785
Okladnikov A	Okladnikov 11	0	0.0-29.91	217,732	0.03	4,698

SI Table 4.3. Summary statistics for the merged Y chromosome BAM files for the 11 remains belonging to 8 individuals. We show the contamination estimates in percent, along with the number of called sites (out of ~6.9 Mb), average coverage and the number of reads overlapping target regions.

Detailed coverage across the Y chromosome

Previous studies of human Y chromosomes have revealed that they are prone to large scale deletions and insertions[112–115]. Therefore we looked for those in both Neanderthal and Denisovan individuals.

Results on simulated data

We first assess our power to detect copy number variations from low-coverage data in simulations. We calculated the coverage in non-overlapping windows of 10 kb along the regions captured with the Y chromosome capture array in our simulated datasets (described in SI 4 - **Modern human contamination estimates**). Because we simulate reads from the reference Y chromosome there should be no major deletions/duplications and the coverage should be uniform.

We see that as the coverage increases the estimated coverage across the captured Y chromosome regions fluctuates less (**SI Figure 4.4**). However, we also observe that it is much harder to estimate coverage in the X-transposed and heterochromatic regions, due to the small number of captured sites (**SI Figure 4.4**) - 52% of the 10 kb windows from the heterochromatic region and 73% from the X-transposed region had less than 1,000 bp captured by the array. In the X-degenerate region, and ampliconic regions, only 2% and 19% of windows had less than 1,000 bp called, respectively. We therefore focus our analysis of copy number variation in Neanderthals and Denisovans on the ampliconic and X-degenerate regions, excluding those 10 kb windows with fewer than 1,000 bases covered.

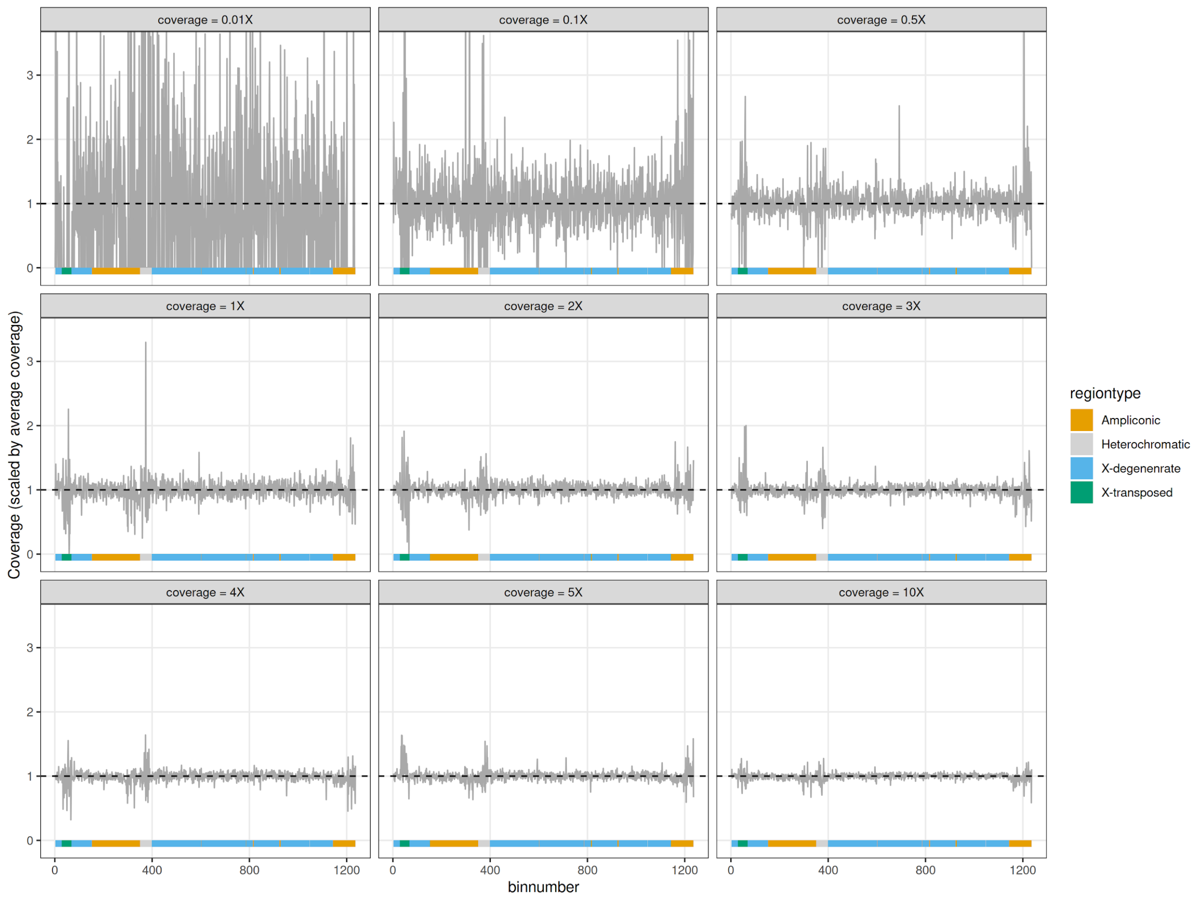

SI Figure 4.4. Coverage across all 10 kb bins (bin numbers) on the Y chromosome. The y-axis is the estimate of the coverage normalized by the mean coverage across all bins. The colors indicate which region class the 10 kb window overlaps.

Results for Neanderthal and Denisovan libraries

In our simulations the reads are evenly distributed across the Y chromosome. However our data might be subject to additional capture biases such as varying GC content. Another concern is that the captured regions (minimum length of 100 bp) are captured by overlapping probes spaced by 3 bp. Thus a site in the middle of a captured region would have more probes overlapping it compared to sites at the beginning of the captured regions.

Indeed, when examining the first problem of the coverage of the end vs middle of a region we see that the coverage at the ends of the captured regions is lower than coverage in the middle of the regions (**SI Figure 4.5)**. Thus, for further analysis for all libraries we exclude the first and last 40 bp of the captured regions.

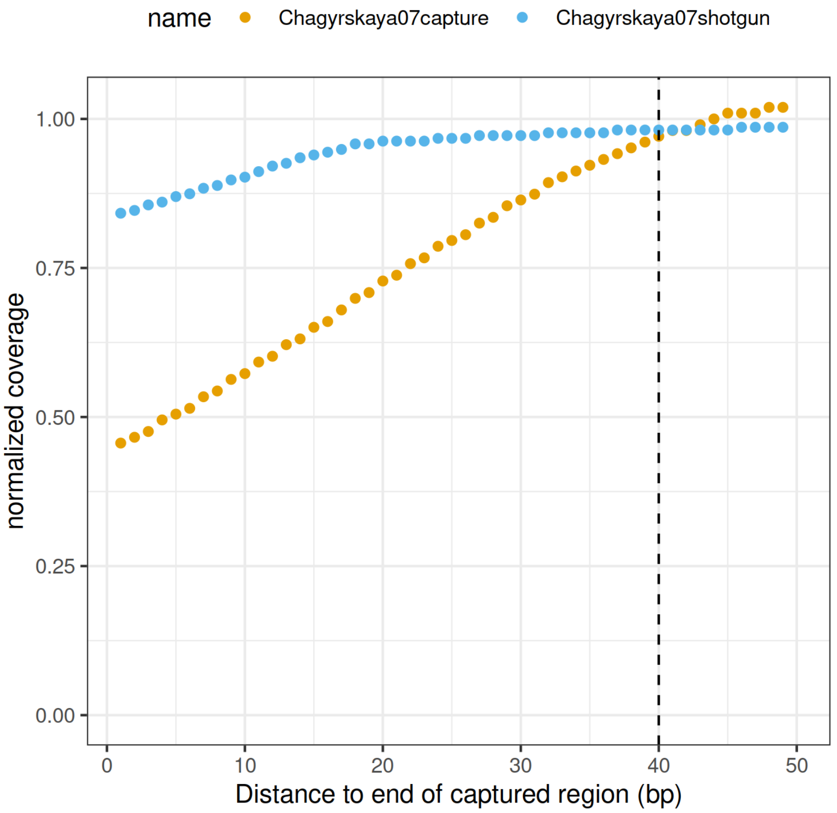

SI Figure 4.5. Normalized coverage as a function of distance to the end of the captured region. The dashed vertical line shows the cutoff we chose. The effect is less pronounced for shotgun data.

Second, we noticed that the coverage increases with the increased GC content of the probe and decreases for shotgun data (**SI Figure 4.6)**. Previous studies have shown a strong GC effect in ancient DNA shotgun data, where coverage decreases for higher GC-content[76]. The capture data show the opposite pattern with regions with higher GC-content being captured more efficiently.

We fit a linear regression for each remain and correct our observed coverage, as shown below:

$$coverage_{corrected} = coverage \cdot \frac{1}{(a \cdot GC_{content} + b)}$$

where *a* is the slope and *b* is the intercept.

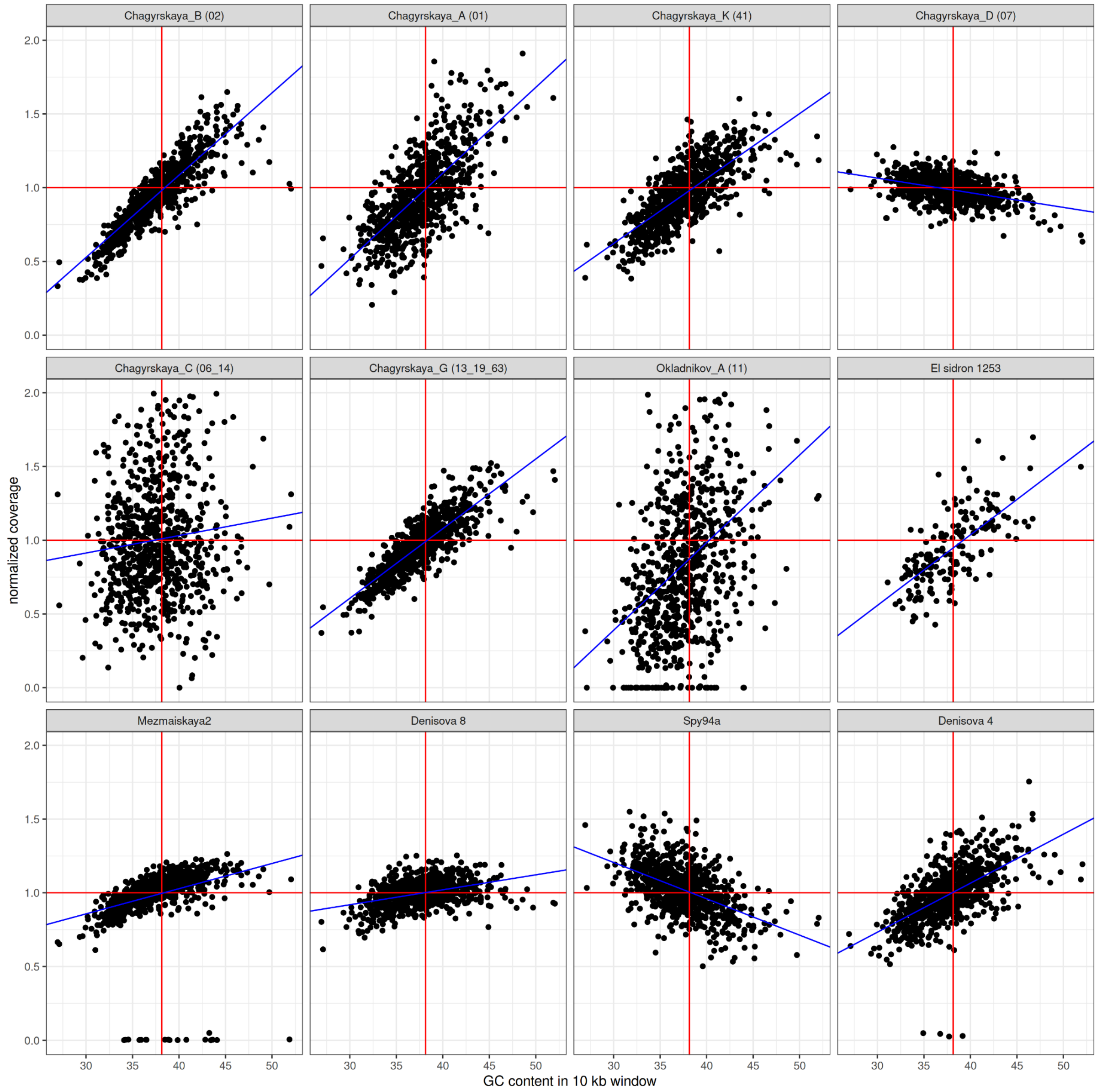

SI Figure 4.6. For each remain we show the correlation between mean GC content in a window of 10 kb vs normalized coverage. The mean GC content (38%) is shown as a vertical red line and the mean coverage is shown as a horizontal red line. The blue line is the fitted linear regression.

We investigated the average coverage of the captured regions in our 8 individuals along with the Y chromosome sequences of *Denisova 4, Denisova 8, Spy 94a, Mezmaiskaya 2, El Sidrón 1253* and two modern human Y chromosomes (S_Han-2, S_French-1). We merge *Chagyrskaya 14_6* into one individual (*Chagyrskaya C*) and *Chagyrskaya 63_13_19* into another individual (*Chagyrskaya G*) (see **SI 7 - Identical individuals**).

The results are shown in **SI Figure 4.7**. We identify 8 structural variants larger than 10 kb (normalized coverage > 1.75 for duplications or normalized coverage < 0.25 for deletions and the coverage should be > 1X).

We compared the structural variants which we identified here to a set of 133 known structural variants identified in 1,244 males from the 1000 genomes project and 411 UK males[114,116].

Position	Size	Found in	Structural variant type	Description	variable in humans
4.89 Mb - 6.81 Mb	1920 Kb	Mezmaiskaya 2	Deletion	Deletion of AMELY, PCHH11Y, TSPY2 And transcription units	no
8.64 MB – 8.66 Mb	20 Kb	All Neanderthals	Deletion	Deletion of transcription unit TTTY11	no
9.64 Mb – 9.66 Mb	20 Kb	All Chagyrskaya Neanderthals, El Sidrón 1253, Spy 94a and both Denisovans	Duplication	Duplication of transcription unit TTTY8	yes
18.0 Mb – 18.20 Mb	200 Kb	All Chagyrskaya Neanderthals	Duplication	Duplication of 200 kb distal to Palindrome 7	no
23.25 Mb – 23.29 Mb	40 Kb	Denisova 4	Deletion	Deletion in X-degenerate	no
23.60 Mb - 23.64 Mb	40 Kb	El Sidrón 1253	Duplication	Duplication of 40 Kb	no
24.35 Mb – 24.38 Mb	30 Kb	Denisova 4 and Denisova 8	Duplication	Duplication of 30 kb of the proximal part of the spacer in Palindrome3	no
24.87 Mb – 24.88 Mb	10 Kb	Denisova 4 and Denisova 8	Duplication	Duplication of 10 kb	no

SI Table 4.4. The genomic location and size of identified duplications and deletions in archaic humans. We also report which remains carry the structural variation and whether the

structural variant is known to be variable in present day human populations.

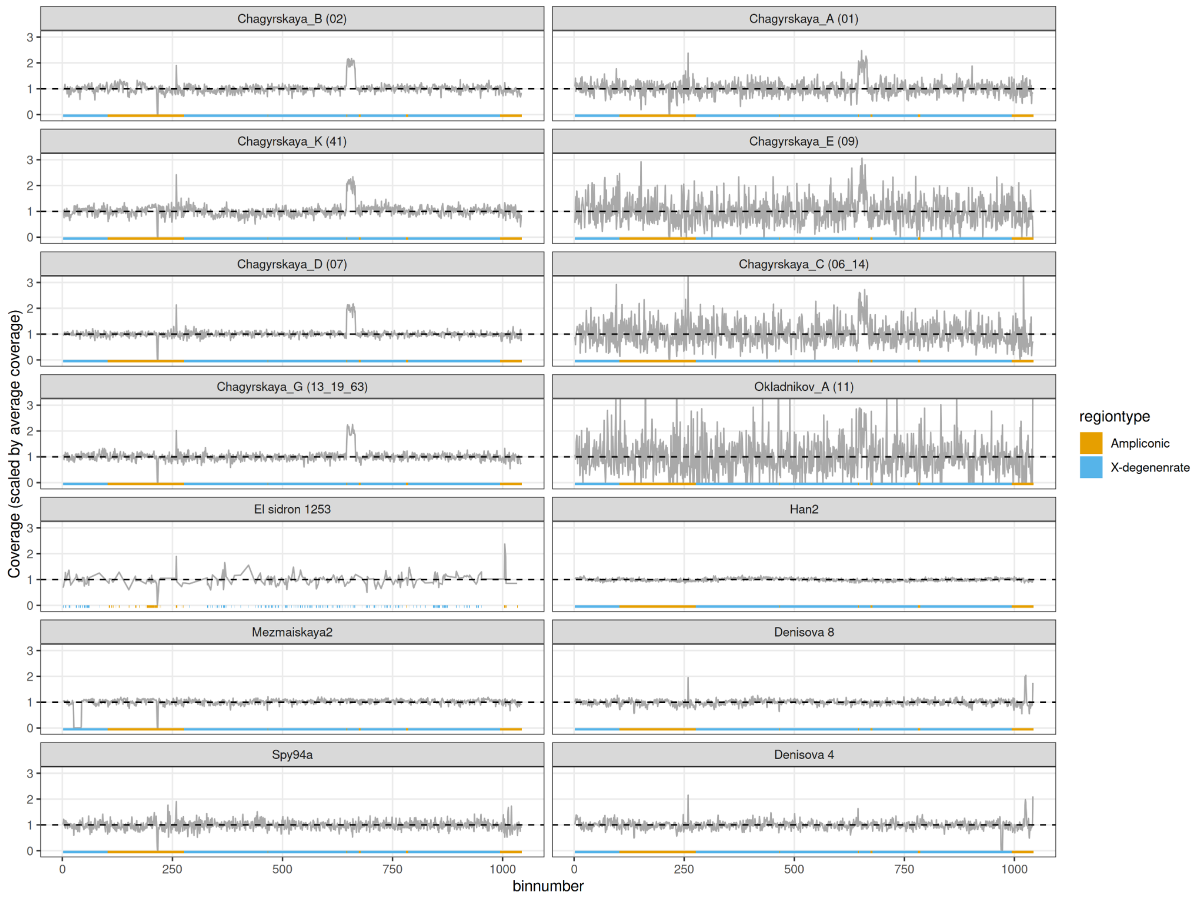

SI Figure 4.7. Coverage across all 10 kb bins (bin numbers) on the Y chromosome. Bins with less than 1,000 bp and bins in the X-transposed and heterochromatic regions from the capture array were removed. The y-axis is the estimate of the coverage normalized by the mean coverage across all bins (dotted line). The colors indicate which region class the 10 kb window overlaps. We show the results for each individual denoted by letters and also show which remains make up that individual in parentheses.

Two of the duplications are located around Palindrome 7 and Palindrome 3. The palindrome arms on the Y chromosome are highly mutable and large scale deletions and insertions are often caused by non-allelic homologous recombination (NAHR)[113,116].

Duplication of sequence up- or downstream of palindrome arms are also frequent in present day humans from the 1000 genomes project[117] and in great apes[118]. We show the duplication distal to palindrome 7 for *Chagyrskaya D* who carries the duplication, and for Mezmaiskaya 2 who does not carry the mutation in **SI Figure 4.8**.

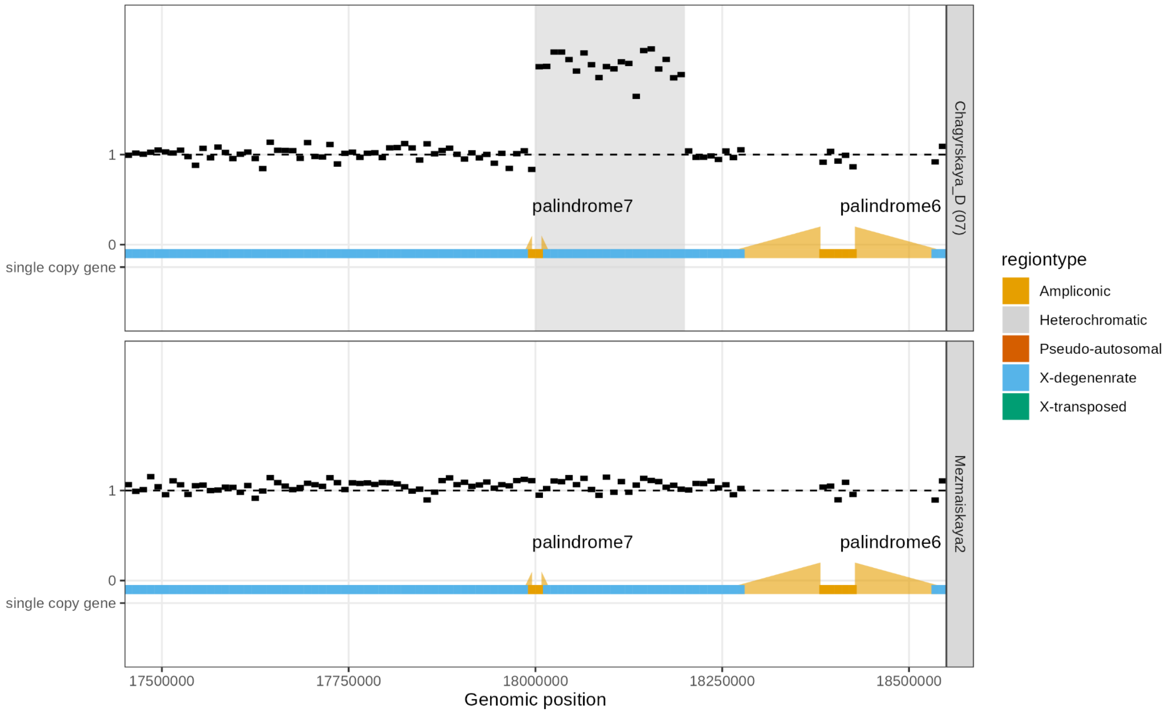

SI Figure 4.8. The duplication of 200 kb of sequence in Chagyrskaya Y chromosomes. We show *Chagyrskaya D*, who carries the duplication (marked in light grey), and *Mezmaiskaya 2*, who does not have the duplication. The X-axis shows the genomic position and the Y-axis the coverage in bins of 10 kb normalized by the Y chromosome wide average coverage. The color of the bins indicate which region classes of the Y chromosome they belong to. Darker regions indicate that they are covered by the Y chromosome capture array (the palindrome arms are not included in the array). We also show the known coding genes and transcription units. The palindromes are marked as triangles and named.

Another noteworthy deletion is carried by the *Mezmaiskaya 2* Neanderthal - see **SI Figure 4.9**. The deleted region contains multiple genes and transcription units including *AMELY*. A 3 Mb deletion containing *AMELY* has previously been reported to be at 2.7% frequency in men from Singapore[119], but it does not seem to have a strong deleterious effect. However it might present a problem when using proteomics to determine sex of an individual as is commonly done in paleoproteomics[120] especially if this deletion exists at an appreciable frequency in late European Neanderthals.

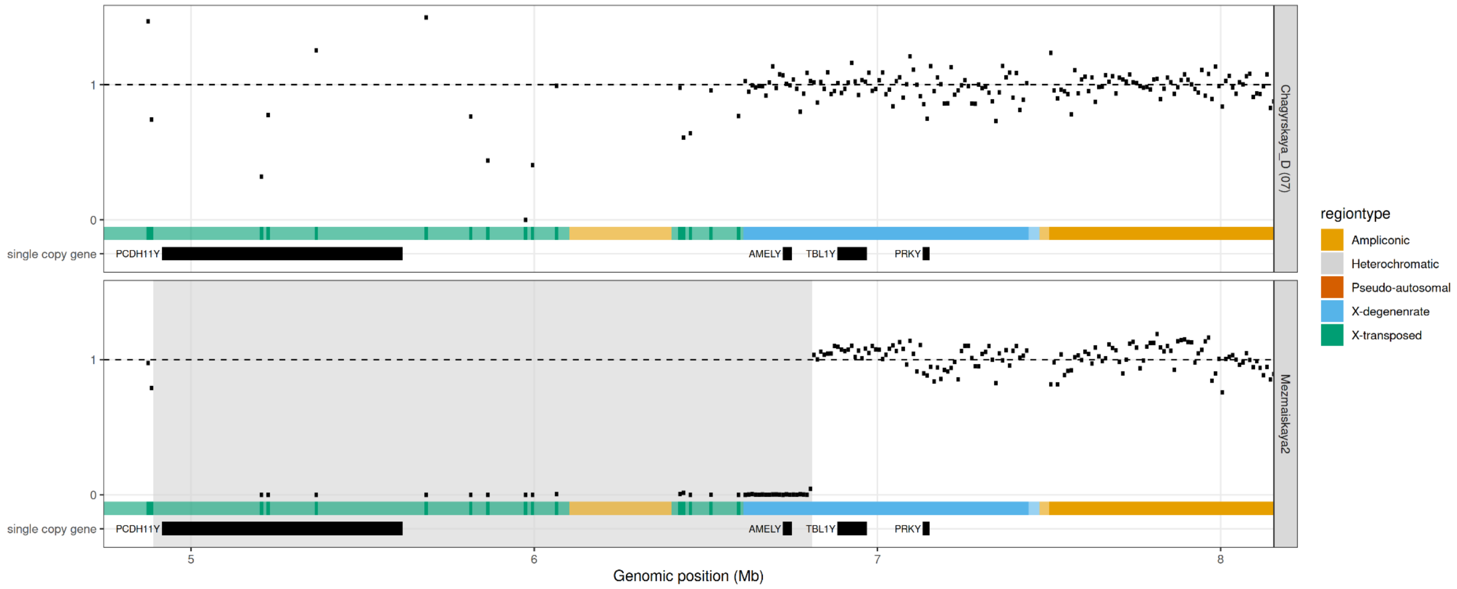

SI Figure 4.9. The deletion of 1.8 Mb of sequence in the Mezmaiskaya 2 Y chromosome (the deletion is marked in lightgrey). We show *Chagyrskaya D* (who does not have the deletion) and *Mezmaiskaya 2*. The X-axis shows the genomic position and the Y-axis the coverage in bins of 10 kb normalized by the Y chromosome wide average coverage. The color of the bins indicate which region classes of the Y chromosome they belong to. Darker regions indicate that they are covered by the Y chromosome capture array. We also show the known coding genes.

Variant calling

For calling variants on the Y chromosome we employed a similar approach as [10]. In short, we only call a base at a given site if the base is supported by at least 3 reads and require that 90% of the reads support the base.

We first tested out the accuracy of this approach using the simulated data described in the **Modern human contamination estimation** section. The results are shown for varying coverages in **SI Table 4.5**. While all the non reference base calls are correct, we do see instances where the reference base has been changed into an alternative base. All the non-reference bases are C>Ts or G>As. This happens because 3 reads overlapping these positions all had a deamination.

We therefore added an additional filter to our C>T and G>A calls. We require at least one of the T's to be on the reverse strand or at least one of the A's to be on the forward strand in any individual carrying the derived allele, if T or A is the derived allele respectively.

		Base is the same as hg19		Base if Neanderthal (n = 1278)	
**coverage**	**cont-amination**	**Ref base Called**	**Wrong alt Base called**	**Alt base Called**	**Wrong Ref Base called**
0.01X	1%	14	0 (0)	0 (0)	0
0.1X	1%	1027	0 (0)	1 (1)	0
0.5X	1%	69961	2 (0)	13 (12)	0
1X	1%	417274	4 (1)	75 (72)	0
2X	1%	1813084	6 (2)	295 (285)	0
3X	1%	3441784	13 (2)	565 (547)	0
4X	1%	4799709	9 (3)	753 (733)	0
5X	1%	5724855	12 (2)	914 (898)	0
10X	1%	6926681	4 (0)	1078 (1074)	0

SI Table 4.5. The number of correctly genotyped reference and alternative bases for varying coverage. The contamination is always kept at 1% and we always require a minimum of 3 reads to overlap the position and that 90% of reads agree on a consensus base. The numbers in brackets are when applying the criteria of minimum one T on the reverse strand if the mutation is a C>T, or requiring a minimum of one A on the forward strand if the mutation is G>A.

Next we investigated the effect of increasing contamination while keeping the coverage at 1X (**SI Table 4.6)**. We notice that variant calls are reliable at our contamination rates of less than 3% (**SI Table 4.3**), and only deteriorate when contamination rates exceed 20%.

		**Base is the same as hg19**		**Base if Neanderthal (n = 1278)**	
**cov-erage**	**cont-amination**	**Ref base Called**	**Wrong alt Base called**	**Alt base Called**	**Wrong Ref Base called**
1X	0%	417990	1 (0)	70 (66)	0
1X	1%	417274	4 (1)	75 (72)	0
1X	2%	414923	2 (0)	75 (71)	0
1X	3%	421168	4 (1)	58 (55)	0
1X	4%	419952	1 (0)	67 (65)	0
1X	5%	419818	0 (0)	61 (57)	0
1X	10%	417360	0 (0)	51 (49)	0
1X	20%	420464	2 (0)	30 (28)	1
1X	30%	422142	0 (0)	17 (17)	0
1X	40%	427384	0 (0)	16 (16)	4
1X	50%	428093	2 (0)	6 (6)	6
1X	60%	433684	0 (0)	5 (4)	20
1X	70%	429835	0 (0)	1 (1)	26
1X	80%	432606	0 (0)	1 (1)	31
1X	90%	434994	0 (0)	0 (0)	53
1X	99.99%	434217	0 (0)	0 (0)	81

SI Table 4.6. We show the number of correctly genotyped reference and alternative bases for varying contamination. The coverage is always at 1X and we always require a minimum of 3 reads to overlap the position and that 90% of reads agree on a consensus base. The numbers in brackets are those obtained when applying the criteria of minimum one T on the reverse strand if the mutation is a C>T or a minimum of one A on the forward strand if the mutation is G>A.

We genotyped variants for all 7 Chagyrskaya individuals, 1 Okladnikov individual, 3 Neanderthals and 2 Denisovans using a set of merged BAM files from curated libraries. We removed variants that fell in the duplicated regions described in **SI 4 - Detail coverage across the Y chromosome.**

As we have less than 20% contamination in our curated merged dataset the biggest source of errors likely stems from deamination of cytosine bases. In order to call variants, we first determine which allele is the ancestral and which is the derived one by using a chimpanzee (panTro4), and previously listed modern humans, Denisovans and Neanderthals.

To check if we are overcalling transitions, we calculated the transition/transversion ratio in 448 present day human males from a previous study[107]. We found 7,284 transitions and 4,453 transversions - thus the transition/transversion ratio is 1.64. In **SI Table 4.7** we list all identified variants.

branch	transitions	transversions	total	Transition/ transversion ratio
Fixed derived in Denisovan	669	422	1,091	1.59
● Variable in Denisovans	27	17	44	1.59
Fixed derived in Human/Neanderthal	467	280	747	1.67
Fixed derived in Neanderthal	841	498	1,339	1.69
● Fixed derived in Mezmaiskaya 2	120	84	204	1.43
● Fixed derived in Spy 94a	30	57	87	0.53
● Fixed derived in El Sidrón 1253	8	11	19	0.73
● Fixed derived in Chagyrskaya	75	49	124	1.53
● Variable in Chagyrskayas	13	2	15	6.5
● Fixed derived in Mezmaiskaya 2 and Spy 94a	6	2	8	3
● Fixed derived in Chagyrskaya and El Sidrón 1253	4	1	5	4
Fixed derived in Human and branch specific	670	391	1,061	1.71
Cannot determine ancestral/derived	354	214	568	1.65
recurrent (breaks topology)	88	16	104	5.5
Total	3,372	2,044	5,416	1.64

SI Table 4.7. We show the number of variants that fall on each major branch (Denisovan, modern Human/Neanderthal, Neanderthal and modern human). We also show variants private to *Mezmaiskaya 2, Spy 94a* and the Chagyrskaya Neanderthals.

We also show variants that could not be placed on the tree (i.e., where we cannot determine ancestral/derived) because the base could not be called in the Chimpanzee Y chromosome. We observe a few mutations that are recurrent (104/5,416 = 1.9%). We note that part of this could be due to errors in the Chimpanzee reference genome as only 3/104 sites are recurrent when the Chimpanzee Y chromosome is removed from the analysis. However, the transition/transversion ratio of 5.5 suggests that ancient DNA damage could also explain some of the sites.

We note that variants that are variable among the Chagyrskaya Neanderthals are still enriched for transitions.

On the other hand, variants that are private to *Spy 94a* and *El Sidrón 1253* are depleted for transitions. This is because we apply a more strict filter to transitions. For instance, if the mutation is C>T we need a T on the reverse strand, and when the coverage is around 3X we might not observe this just by chance.

However, variants that are observed in multiple individuals (such as all Chagyrskaya Neanderthals) have a transition/transversion ratio much closer to the expected value of 1.64.

Lastly we note that due to low coverage on the Denisova Y chromosomes we are likely not identifying all variants. The highest coverage Denisova Y chromosome has a coverage of 3.5X. From simulated data presented in **SI Table 4.5** we estimate that we likely only find between 42.8% (at 3X) and 57.3% (at 4X) of all the variants. If we undercall variants on one branch by say 50% we expect the total coalescence time to be decreased by 25%. When we do the BEAST analysis in **SI Figure 4.10** the coalescence time for Denisovan and Neanderthals is around 500 ka which is 70% less than previously reported 700 ka[10] - this is consistent with the coalescence time being 700 but due to undercalling of varying its reduced by around 25%.

Phylogenetic analysis

We constructed a phylogenetic tree using a total of 6.5e6 sites (7,152 sites are varying including 5,416 sites from **SI Table 4.7** and 1,736 sites that vary between the hg19 reference genome, S_Han-2, S_French-1 and A00), excluding *Chagyrskaya A, Chagyrskaya C, Chagyrskaya E* and *Okladnikov A* due to low amounts of data (**SI Table 4.8**).

We used the Tamura-Nei 1993 (TN93)[98] substitution model with a strict clock of 7.9e-10 mutations per year and bayesian skyline coalescent model. Using this model, three Markov chain Monte Carlo (MCMC) runs of 10,000,000 iterations (75,000 pre-burn iterations) and sampling every 5,000 trees were completed. A summary of a single tree was identified by using the tree annotator program from BEAST2 on the combined tree file. The programs Tracer (version 1.7)[99] and Figtree (version v1.4.4)(https://github.com/rambaut/figtree) and BEAST2 [96] were used to examine the resulting tip dates and TMRCA estimates (**SI Figure 4.10**).

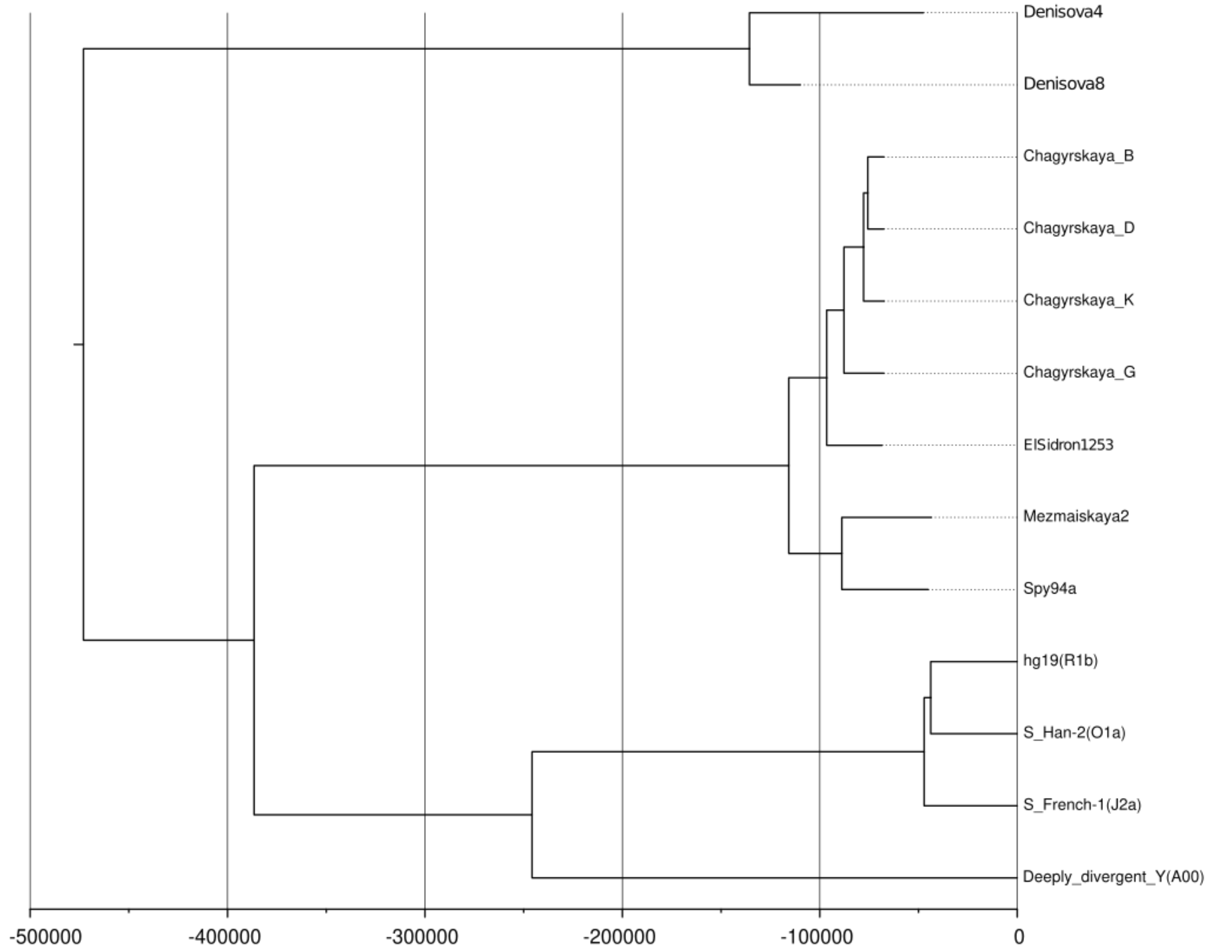

SI Figure 4.10. Maximum likelihood tree using BEAST2. Branch lengths are drawn to scale measured in years given a mutation rate of 7.9e-10 mutations per year. For present-day human Y chromosomes we add the haplogroup in brackets.

As can be seen from **SI Figure 4.10** our topology differs from [10] in that *Mezmaiskaya 2* and *Spy 94* form a clade (instead of *El Sidrón 1253* and *Mezmaiskaya 2* forming a clade as in [10].

In [10] only transversions were considered when constructing the phylogenetic tree. There are two positions supporting the *El Sidrón 1253* and *Mezmaiskaya 2* grouping together and these positions are Y:6,932,831 and Y:6,932,839. *Mezmaiskaya 2* and *El Sidrón 1253* have the derived variant, and *Spy 94a* has the ancestral allele at positions Y:6,932,831 and Y:6,932,839.
These two variants being human-like in *Spy 94a* are likely due to present-day human contamination in *Spy 94a*. The position is covered by four reads, all which also have the derived state at Y:6,932,831, which is a defining variant on the human BT branch. In addition all reads have a read length > 90bp, which is much longer than the data-set average length of 66bp. Long reads are more likely to be contaminants [121,122].

Another estimate that differs is the coalescence time of Denisovan and modern human/Neanderthals. This is due to undercalling of variants on the Denisovan branch which is discussed above.

Placing low coverage samples on phylogenetic tree

We note that the phylogenetic tree (**SI Figure 4.10**) is mainly based on 4 individuals with high coverage - the other 4 individuals only have more than 3 reads at less than one percent of sites (**SI table 4.8**).

Individual name	remain Name(s)	Sites with >= 3 reads	total sites	percentage
Chagyrskaya B	Chagyrskaya 2	5,134	5,416	94.79
Chagyrskaya K	Chagyrskaya 41	3,096	5,416	57.16
Chagyrskaya D	Chagyrskaya 7	2,388	5,416	44.09
Chagyrskaya G	Chagyrskaya 13,19,63	1,171	5,416	21.62
Chagyrskaya A	Chagyrskaya 1	31	5,416	0.57
Chagyrskaya C	Chagyrskaya 6,14	8	5,416	0.15
Chagyrskaya E	Chagyrskaya 9	3	5,416	0.06
Okladnikov A	Okladnikov 11	0	5,416	0

SI Table 4.8. For each individual we show the numbers of varying sites on the Y chromosome supported by 3 or more reads.

In order to place the low coverage individuals (*Chagyrskaya A* (01), *Chagyrskaya C* (06_14), *Chagyrskaya E* (09) and *Okladnikov A* (11)) on the tree we counted the proportion of derived reads at diagnostic positions from **SI Table 4.7** for the Neanderthal/modern human branch, the Neanderthal branch, the *El Sidrón 1253*/Chagyrskaya branch and the private Chagyrskaya branch. (Note the numbers will not amount to 100% due to present-day human contamination and deamination).

	Human nean			Neanderthal			El Sidrón 1253 and Chagyrskaya			Chagyrskaya		
**Individual name**	**derived**	**total**	**percent**	**derived**	**total**	**percent**	**derived**	**total**	**percent**	**derived**	**total**	**percent**
Chagyrskaya B	26,659	26,775	99.57	27,705	27,873	99.4	130	132	98.48	2,609	2,625	99.39
Chagyrskaya K	4,378	4,455	98.27	2,901	3,087	93.97	12	12	100	284	301	94.35
Chagyrskaya D	1,437	1,456	98.7	1,950	2,006	97.21	10	10	100	194	197	98.48
Chagyrskaya G	1,082	1,114	97.13	867	934	92.83	3	4	75	93	97	95.88
Chagyrskaya A	180	186	96.77	152	157	96.82	1	1	100	12	13	92.31
Chagyrskaya C	68	72	94.44	64	68	94.12	0	0	0	11	11	100
Chagyrskaya E	30	30	100	36	39	92.31	0	0	0	6	6	100
Okladnikov A	13	13	100	15	15	100	0	0	0	3	3	100

SI Table 4.9. For each individual we show the number of reads mapping to sites on a branch, how many of these are derived, and the percentage of derived reads highlighted in green. If there are no reads supporting a branch it is highlighted in yellow.

We find that the low coverage samples have most of the derived alleles for Human-Neanderthal-shared. Neanderthal-specific and Chagyrskaya-specific branches (**SI Table 4.9)**

Variant annotation

We wanted to investigate if any of the 5,416 mutations had any putative function so we annotated the variants using UCSC Variant Annotation Integrator[123] and searched for non-synonymoys mutations. We find 12 missense variants and one gained stop codon (**SI Table 4.10**). We do not have any reads overlapping 3 previously reported variants from[124]: Y:2,844,774 (which is contained in the Y chromosome array), Y:4,967,724 and Y:5,605,569 (which are in part of the X-transposed region not included in our Y chromosome array).

Our analysis confirms the previous findings that many Y chromosome mutations occur in genes that produce male-specific minor histocompatibility (H-Y) antigens[124].

position (hg19)	Reported previously in [124]	ref	alt	mutation (anc>dev)	variant type	Gene	branch
6,932,032	yes	G	C	G->C	missense_variant	*TBL1Y*	Fixed derived in Neanderthals
6,932,181	no	C	G	C->G	missense_variant	*TBL1Y*	Fixed derived in Denisovans
6,955,466	no	A	G	A->G	missense_variant	*TBL1Y*	Fixed derived in Denisovans
14,832,610	yes	A	G	A->G	missense_variant	*USP9Y*	Fixed derived in Neanderthals
14,832,620	yes	G	T	T->G	missense_variant	*USP9Y*	Fixed in branch F in humans
14,838,553	yes	G	A	G->A	missense_variant	*USP9Y*	Fixed derived in El Sidrón 1253
14,952,462	no	T	A	A->T	missense_variant	*USP9Y*	Fixed derived in Humans/Neanderthals
15,816,262	yes	C	A	-	stop_gained	*TMSB4Y*	Stop is fixed in Neanderthals
21,629,240	no	C	G	G->C	missense_variant	*BCORP1*	Fixed derived in humans
21,868,167	yes	C	T	T->C	missense_variant	*KDM5D*	Fixed derived in humans
21,871,613	no	C	T	C->T	missense_variant	*KDM5D*	Fixed derived in Chagyrskayas
21,877,887	no	C	T	C->T	missense_variant	*KDM5D*	Variable in Chagyrskayas
21,905,071	yes	C	T	T->C	missense_variant	*KDM5D*	Fixed derived in humans

SI Table 4.10. Amino acid changing mutations found in our dataset.

Dating of Y chromosomes

We wanted to estimate the age of the Chagyrskaya remains using the Y chromosomes using molecular branch shortening as done in [2].

We use a mutation rate of 7.3e-10 mutation per bp per year as estimated in the previous study [10]. We use *Chagyrskaya B* since it has the highest coverage (we can access the full ~6.9 Mb of Y chromosome regions). Since there are fewer than 5 differences between any pair of Chagyrskaya Y chromosomes, we expect the results for the other individuals to be very similar..

As "anchor points" we use both the divergence time of the modern human and Neanderthal Y chromosomes which is 370,000 years ago (95% CI is 326,000 - 420,000 years ago) and the divergence time of the *Mezmaiskaya 2* and *Spy 94a* Neanderthals which is 89,863 years ago (95% CI is 74,454-108,808).

We find that the genetic age of *Chagyrskaya B* is approximately 64,000 years (95% CI is 48,000-83,000 years ago) when using the divergence time of the Neanderthal Y chromosomes.

**Anchor point**	**mutations**	**Divergence time**	**Branch length estimate**
Human/Nean	1470	370 (326 – 420)	79 (35-129)
Mez2,Spy94a,Chagyrskaya	131	90 (74-109)	64 (48-83)

SI Table 4.11. Number of mutations, estimated divergence time and genetic age using Y chromosome branch shortening estimate.

SI 5 Nuclear DNA capture and sequencing

The vast majority of libraries generated for this project have low complexity and low percentage mapped **(SI Data 1 - Initial Shotgun)**. Thus, we use a capture approach to enrich for sites that are of Neanderthal origin, focusing on a novel array that targets Neanderthal variation. In this section, we first describe the new array and the selected sites, and then the subsequent sequencing and processing of the data.

Array design

We designed an array to capture nuclear variation in archaic populations. The array was designed in the following way:

Samples

The array is based on 504 African individuals from the 1000 genomes phase 3 dataset[125]:

- ESN (Esan in Nigeria, n=99)
- GWD (Gambian in Western Division, n=113)
- LWK (Luhya in Webuye, Kenya, n=99)
- MSL (Mende in Sierra Leone, n=85)
- YRI (Yoruba in Ibadan, Nigeria, n=108)

and on four high-coverage archaic genomes:

- Altai Neanderthal (Denisova 5)[75]
- Vindija 33.19[76]
- Chagyrskaya 8[2]
- Denisova 3[111]

Filtering

Arrays are filtered for:
SNPs

- biallelic transversions
- combined Manifesto-filter (Altai, Vindija, Denisova, Chagyrskaya)[2,75,76,111] which includes:
  - -a filter that excludes tandem repeats and inserts-deletions (called with GATK), coverage filter stratified by GC content (with a minimum of 10X)
  - -a minimum mapping quality score of 25
  - -a mappability filter that requires all 35mers to be unique[76].
- flanks (105 bases)
- mappability-track 100 [75]
- hg19 bases in {A,C,T,G}
- 105 aligned panTro4 bases in {A,C,T,G,a,c,t,g}
- Repeat Masker (hg19 + panTro4)
- Tandem Repeat Finder (hg19 + panTro4)

Ascertainments

We use two strategies to enrich for sites of interest. The archaic variation component focuses on variation between the four sequenced archaic humans. The African variation panel, on the other hand, focuses on SNPs that are at high-frequency in Africans. Such SNPs are likely to be old and were thus likely variable in the common ancestor of humans and Neanderthals:

African variation component (AFR_combined n=465,978)

- SNPs with derived allele frequency > 10% (AFR_high n=444,899)
- SNPs uniformly sampled from 2%-frequency-bins (AFR_uni n=209,800)

Archaic variation component (ARC_combined n=396,750)

- SNPs varying among the four archaic genomes (ARC_var n=301,751)
- fixed differences between archaics and Africans (ARC_fix n=12,123)
- sites fixed derived in archaics, while not fixed derived in Africans (ARC_fixdev n=92,190)

The chimp reference genome (*panTro4*) was used to infer the ancestral state. Sites within components do not sum up because of duplicates between ascertainment schemes. A summary of how sites overlap between the ascertainment schemes is shown below in **SI Table 5.1**.

	**AFR_high**	**AFR_uni**	**ARC_var**	**ARC_fixdev**	**ARC_fix**
**AFR_high**	444,899				
**AFR_uni**	188,721	209,800			
**ARC_var**	76,253	42,432	301,751		
**ARC_fixdev**	72,979	43,651	0	92,190	
**ARC_fix**	0	0	0	9,314	12,123

SI Table 5.1. Overlapping sites between different ascertainment schemes.

There are 712,373 unique sites in the array and 150,355 sites are present in both the ARC_combined and AFR_combined components **SI Table 5.2**.

	AFR_combined component	Present in both components	ARC_combined component
**Number of sites**	465,978	150,355	396,750

SI Table 5.2. Overlapping sites between array components.

Probe Design

Probe sequences were generated based on the sequence of the *hg19* modern human reference genome. For each SNP there are a total of four probes, each of 52 bp in length:

- two probes with the SNP in the middle, one with the REF, the other with the ALT allele
- two probes flanking the SNP without overlapping it

An 8-nucleotide (nt) linker sequence (CACTGCGG) was attached to the 3' end of each probe, for a total probe length of 60 nt, and the probes synthesized on 1 million feature arrays (Agilent Technologies) and converted into probe libraries as described elsewhere[63].

Captured libraries

We captured an aliquot of each of the 49 amplified libraries from the 17 Chagyrskaya and Okladnikov remains by two rounds of in-solution hybridization capture[63] automated on the Bravo NGS Workstation[64] (see **SIdataset1 - Nuclear capture tab)** and sequenced pools of enriched libraries on the HiSeq 2500. In addition to the nuclear capture data we also use all shotgun sequences available for *Chagyrskaya 7*.

We aligned all BAM files to the hg19 human reference genome and filtered them for reads with a mapping quality greater than 25 (MQ⩾25) and a read length greater than 35 (L⩾35) - later we additionally included reads longer than 30 bp (L⩾30). In addition to this all reads must overlap the target SNPs contained on the capture array.

We counted the number of C to T changes, from the 5' and 3' end of the read for each library. The results are shown in **SIdataset1 - Nuclear capture tab.** We observe that all libraries show a damage pattern that is consistent with the presence of authentic ancient DNA. We do not observe strong ancient DNA damage patterns for the negative control libraries (a maximum of 60 reads with a C>T or G>A substitution in the the first/last 3 positions of the read in a single library (**SIDataset1 - Nuclear capture tab**)).

To reduce reference bias we mapped the sequence reads to both the human reference genome and to a modified genome carrying an alternative allele for each of the variants on the array [73]. We retain only reads that have a length > 30 bp.

Capture efficiency and bias

After the alignment and filtering of the BAM files using a length cutoff of 35 base pairs and a quality cutoff of 25, we obtained the coverage per captured site for each sequenced library.

Six sites were covered by all libraries, and 11,932 sites were never covered in any of our libraries. The mean coverage of the target sites per library is 1.03 and the mean GC content per probe is 0.41 (GC content was calculated by counting the proportion of G and C nucleotides within 52bp up- and downstream of the target site). Sites that were never covered are in probes that have a low GC content (average GC content of 0.25) compared to sites that are covered (which are in the probes that have an average GC content of 0.42). Since some libraries originate from the same bone/tooth, we merged BAM files from all libraries from the same remain using samtools (version 1.3.1-21), which resulted in 35,605 sites covered by all remains. See **SI Figure 5.1**

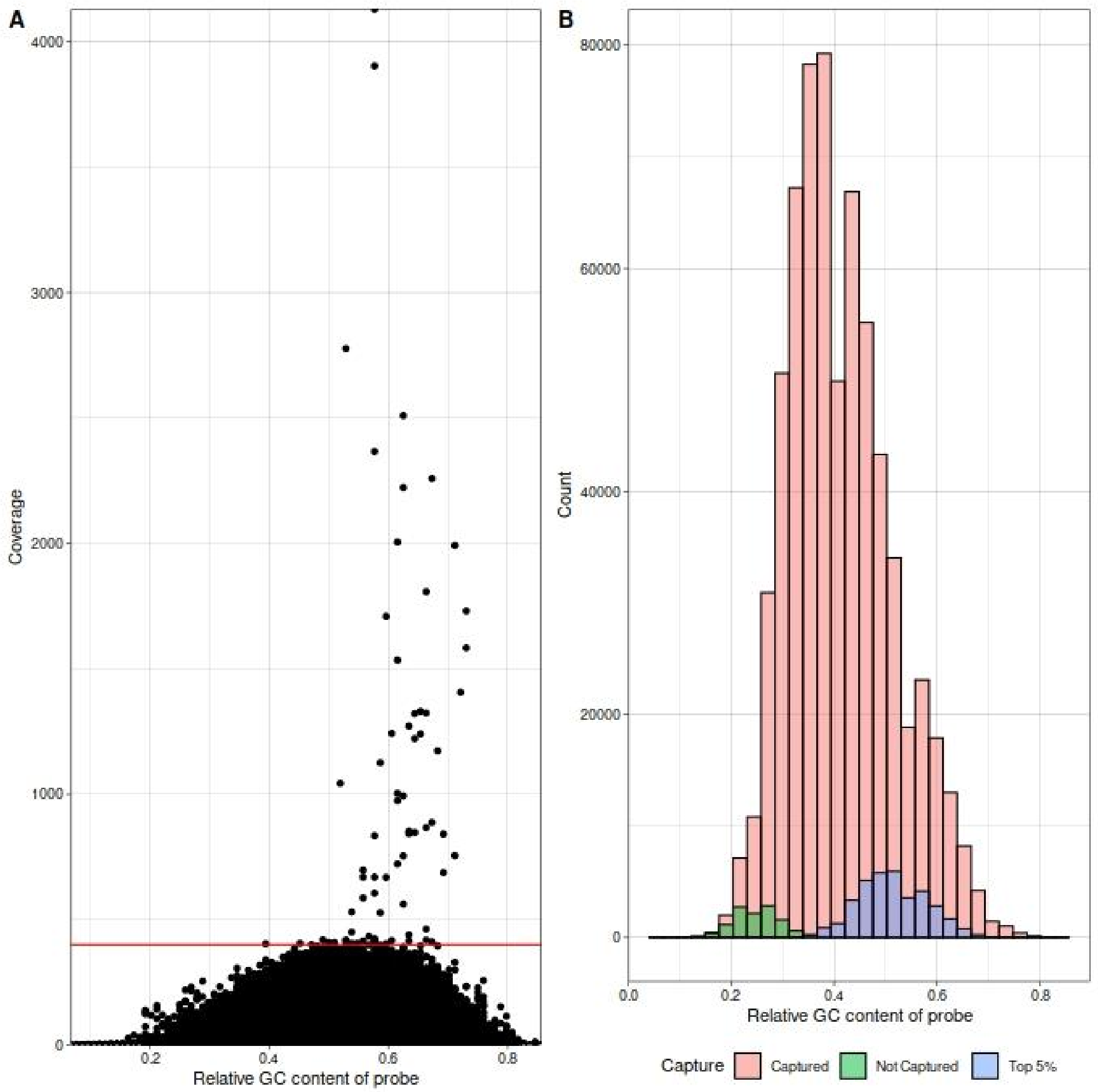

SI Figure 5.1. **A)** Coverage per GC content of capture probe **B)** Frequency of probes with a certain GC content. Red = captured sites, blue = highly captured sites, green = sites never captured.

To make sure there is no systematic bias in the capture process to preferentially capture particular chromosomes or to discriminate against some, we checked the coverage per each chromosome (**SI Figure 5.2**). We also observe a subset of sites with a very high coverage (>400x when merging all data sets). The coverage is likely increased because the probes at these sites are capturing highly conserved or repetitive sequences and we therefore remove them from all downstream analyses (**SI Figure 5.2**).

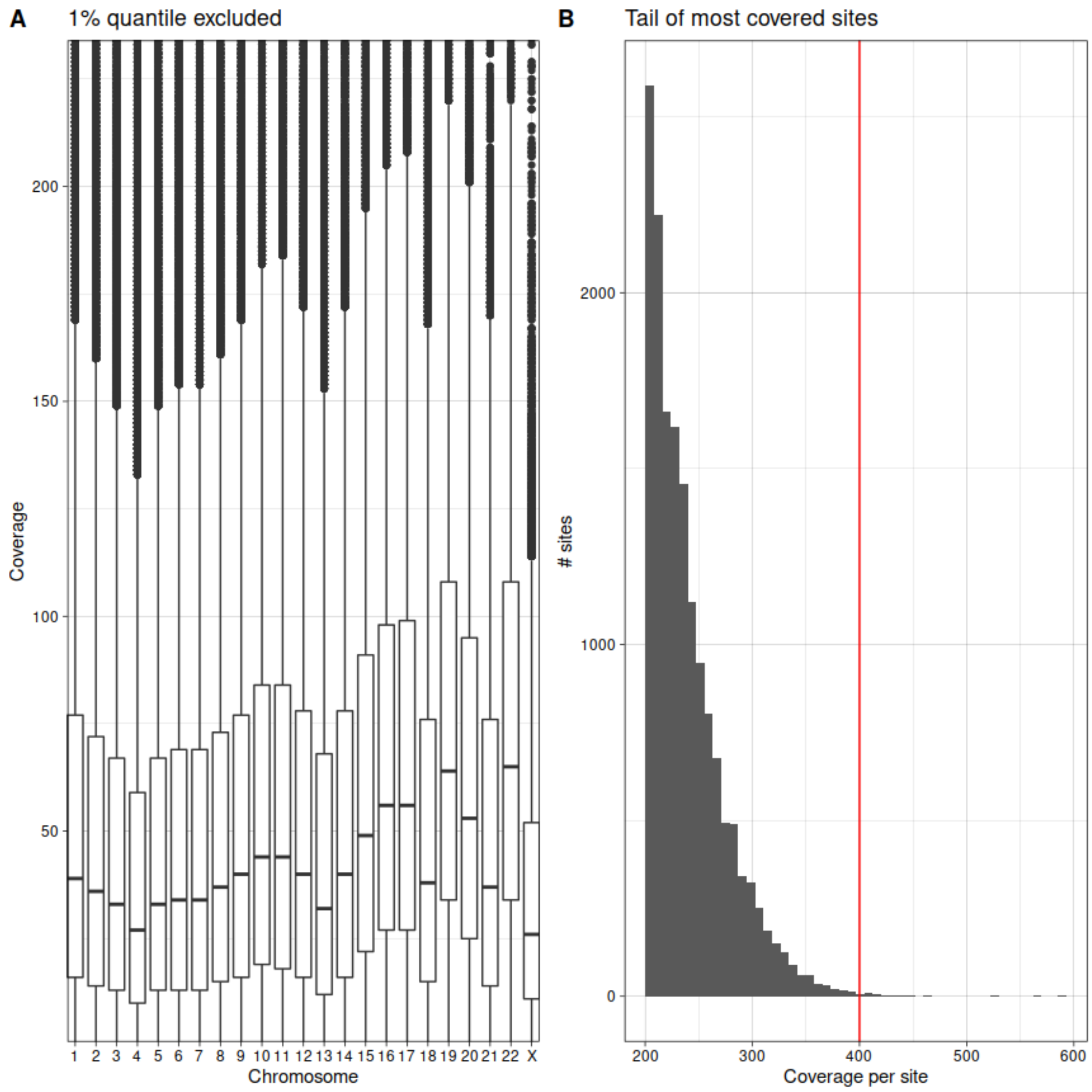

SI Figure 5.2. **A)** Coverage per chromosome excluding highest 1 percentile (minimum number of snps per chromosome e.i n=8,258). The horizontal line in the boxplot denote the median, the bounds of the box contains 50% (the distance from the lower 25% of the data to the upper 25% of the data is called the inter-quartile range) of the data and the whiskers denote 1.5 times the inter-quartile range. **B)** Tail of the distribution of coverages per site. The red line indicates the upper cutoff.

Probes found both in archaic component and African component

The sites included in the Archaic component (ARC_combined) and the African component (AFR_combined) of the array are partially overlapping. Hence, some SNPs (n=150,355) were targeted twice. We therefore checked if these sites are more likely to be covered when compared to the sites in only one of the two sets. This is indeed the case as we show in **SI Figure 5.3** - and we therefore excluded these sites for sex-determination, which relies on estimating the average relative coverage across sites on the autosomes and the X chromosome.

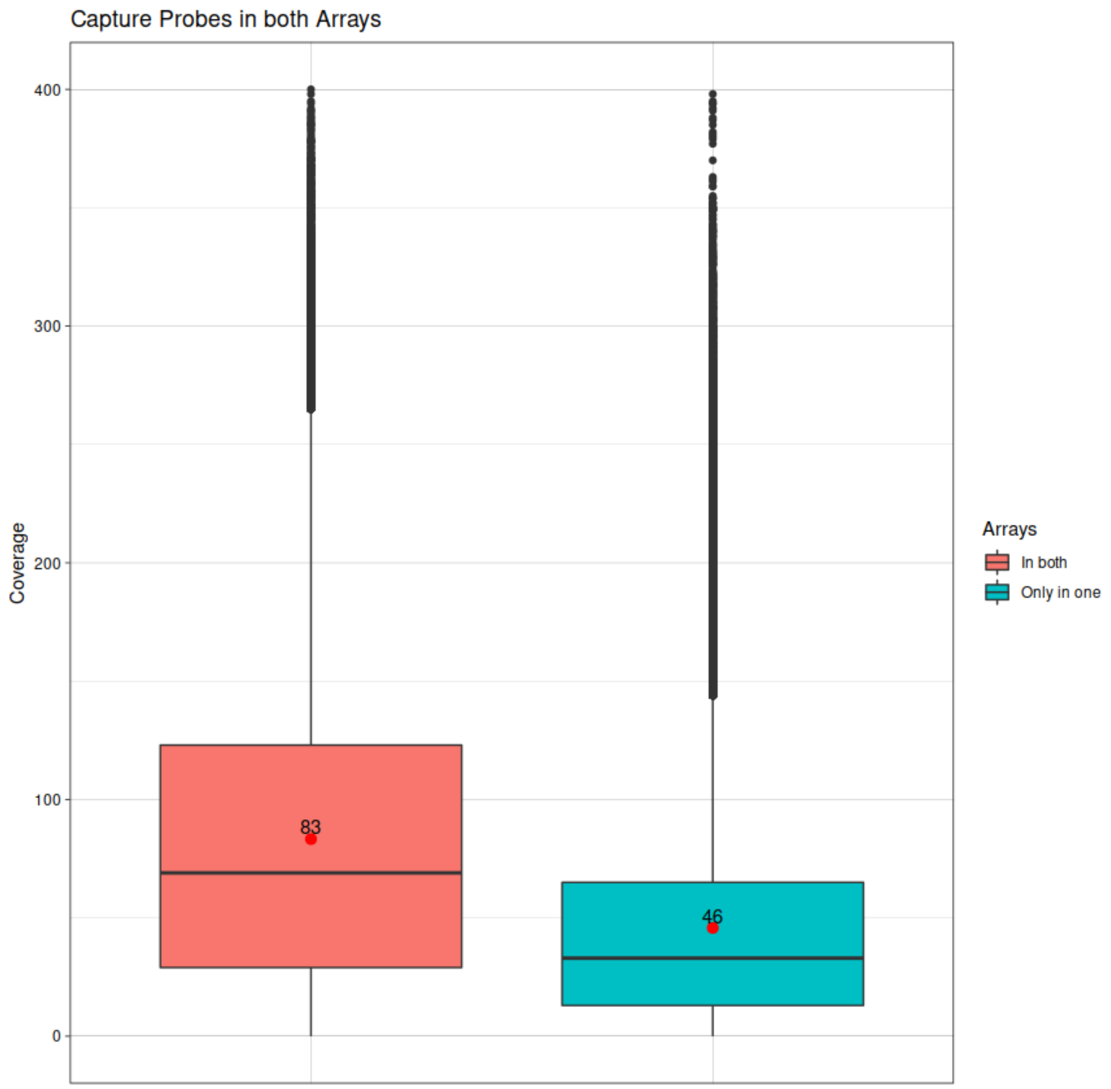

SI Figure 5.3. Coverage of capture sites contained in both components (n=150,355) vs. only in one component (n=562,019). The horizontal line in the boxplot denote the median, the red dot denote the mean value and the bounds of the box contains 50% (the distance from the lower 25% of the data to the upper 25% of the data is called the inter-quartile range) of the data and the whiskers denote 1.5 times the inter-quartile range.

Probes that overlap other probes

We found that some captured sites are very close to each other, which can be problematic if they are in the range of a probe that is designed to capture a neighboring site. As detailed previously in the **SI 5 - Probe design** section, each capture site of interest is targeted by four different probes. If we call the target site of a probe *x*, then two of the probes are centred on the site *x* and carry either the reference (REF) or the alternative (ALT) allele, as well as 26 bp downstream and 25 bp upstream of *x*. Additionally, two flanking probes cover the 52-bp on either side of *x* up- and downstream, but do not overlap *x* directly (**SI Figure 5.5**).

If there is another site *y* less than 52 bp from the captured site *x*, *y* will be targeted by the flanking part of the probe for *x*. Since the flanking part of the probe only contains the reference sequence, the probes capturing *x* will also capture *y* but may be biased towards capturing molecules that match the reference. This might lead to a reference bias, where in the worst case for a couple of SNPs which are less than 25 bp apart from each other only $1/5$ are preferentially targeting the alternative allele (**SI Figure 5.4**).

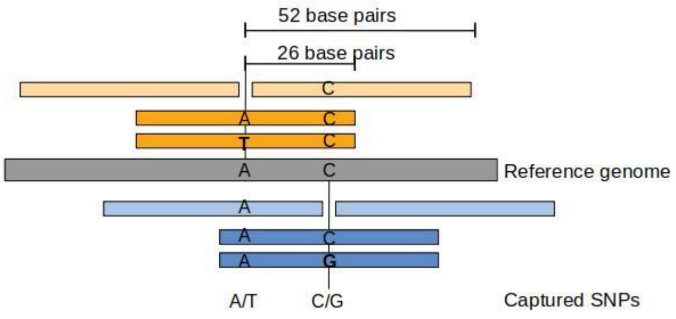

SI Figure 5.4. Depiction of probes overlapping multiple capture sites - the A/T site and the C/G site. The Reference genome is shown in grey with the reference allele marked. Each site has a total of four probes covering and surrounding it (A/T SNP in orange and C/G SNP in blue). In this example the A/T SNP is captured directly by 5 probes, but only one of them contains the alternative allele.

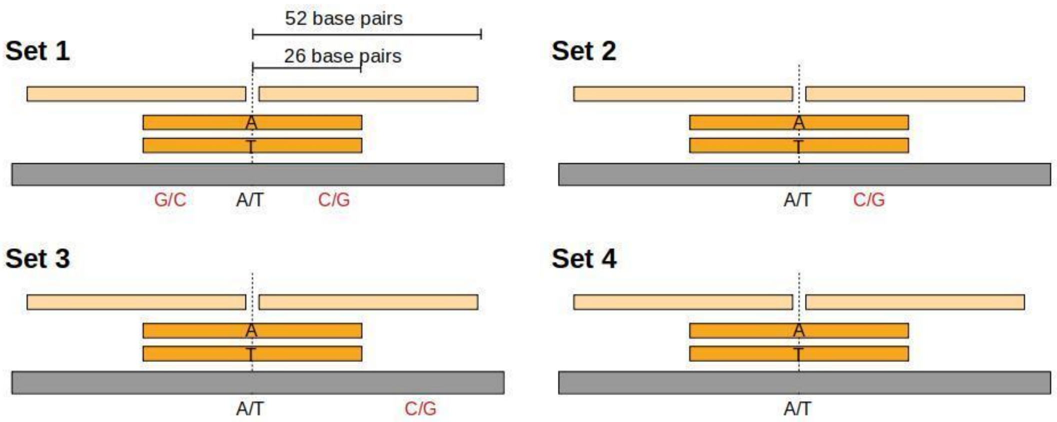

SI Figure 5.5. Probes overlapping any given site of interest. The grey bar represents the reference genome. In this example the site of interest (vertical dashed line) contains two possible alleles: “A” or “T” . The probes containing each of the two alleles are shown in dark orange. Flanking probes that do not overlap the site are shown in light orange. We show four different scenarios corresponding to four different sets of sites. **Set1**: There are a minimum of two other variants (in red) within 26 bp of the site of interest. **Set2**: There is one other site (in red) within 26 bp of the site of interest. **Set3**: There is one other site (in red) within 52 bp of the site of interest. **Set4**: There are no other sites within a distance of 52 bp of the site of interest.

We search for all variants which are within 52 bp of targeted sites, and partition these sites into 4 sets of local SNP density (**SI Figure 5.5**):

- Set 1: SNPs where the capturing probes overlap two or more additional capture sites that are within 25bp upstream AND/OR 26bp downstream of the target site *x* (1,003 sites)
- Set 2: SNPs where the capturing probes overlap at least one additional capture site that is within 25bp upstream or 26bp downstream of the target site *x* (41,058 sites)
- Set 3: SNPs where the capturing probes overlap additional capture sites that are within 52bp upstream or downstream of the target site *x* (68,876 sites)
- Set 4: SNPs where the capturing probes uniquely overlap the capture site *x* (643,497 sites)

Set 3 includes Set 1 and Set 2, while Set 2 includes Set 1. For each of these sets we check the reference bias making use of the fact that one of the remains, *Chagyrskaya 12*, (libraries: F3116 and F8790) belongs to the same individual as *Chagyrskaya 8*, which was previously shotgun sequenced to high coverage (see **SI 6 - Chagyrskaya 12 is the same individual as Chagyrskaya 8**). Since our capture array includes heterozygous positions from the high coverage genome of *Chagyrskaya 8*, we expect the same positions to be heterozygous in *Chagyrskaya 12*. Using targeted positions which are in close proximity to others we are able to check whether there is an equal representation of the reference and alternative alleles, or if the over-targeting of the reference alleles leads to a reference bias.

First we ascertained heterozygous sites in the high coverage genome of *Chagyrskaya 8* using previously generated VCF files[2]. We obtained a file containing the chromosome, position, reference base and the allele information for all heterozygous sites.

Next, we intersected this file with the sites included on the capture array, and which was used for the nuclear captures of *Chagyrskaya 12* and other remains included in this study. The intersect file in the bed format contains the chromosome, 0-based position of the SNP, +1-based position of the SNP, a reference and an alternative allele of all heterozygous sites in the *Chagyrskaya 8* high coverage genome, and which are also present in the capture array. We show the results in **SI Figure 5.6.**

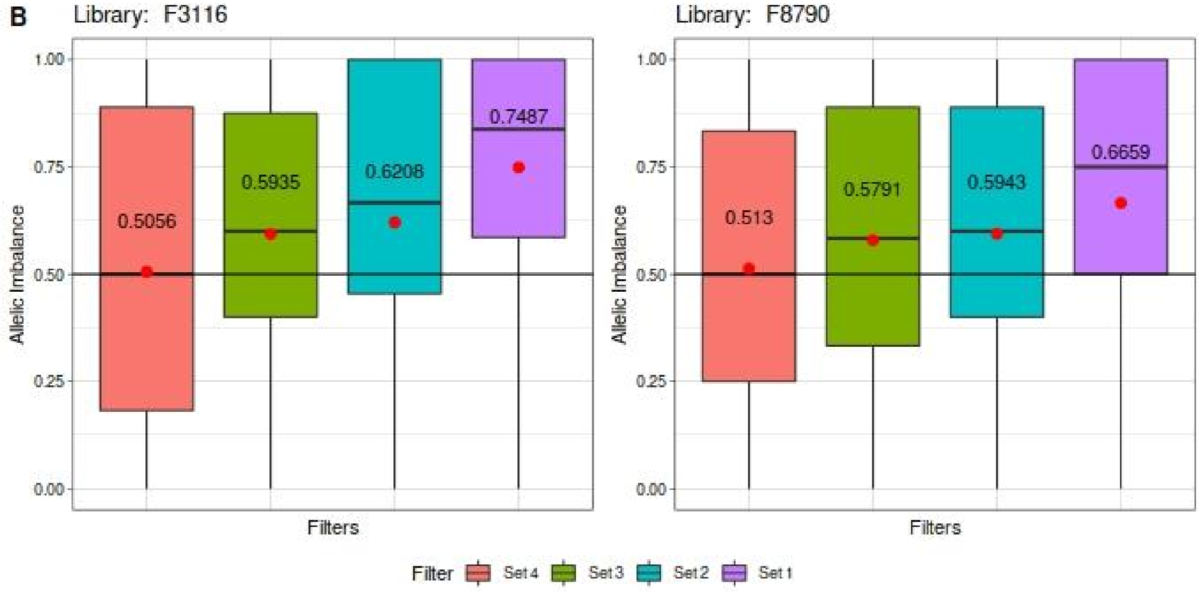

SI Figure 5.6. Local SNP density and resulting allelic imbalance of the two libraries for the different local SNP density sets. The red dot is the mean allelic imbalance. The horizontal line in the boxplot denote the median, the bounds of the box contains 50% (the distance from the lower 25% of the data to the upper 25% of the data is called the inter-quartile range) of the data and the whiskers denote 1.5 times the inter-quartile range. Set 1 (n=1,003) are sites captured with probes overlapping two or more additional capture sites that are within 25 bp upstream AND/OR 26bp downstream of the target site, Set 2 (n=41,058) are sites captured with probes overlapping at least one additional capture site that is within 25 bp upstream or 26 bp downstream of the target site, Set 3 (n=68,876) are sites captured with probes overlapping additional capture sites that are within 52 bp upstream or downstream of the target site and Set 4 (n=643,497) are sites only captured by a unique probe. We show the allelic imbalance for each individual set.

From **SI Figure 5.6** we conclude that only the sites in Set 4, i.e. sites captured with probes that uniquely overlap the site of capture site, do not show a reference bias. from the 713k sites originally captured, we therefore exclude 77 sites (coverage > 400x) and the 68k SNPs in set 3 (including set 1 and 2), leaving 643,472 sites for all downstream analyses.

Modern human contamination estimates

We estimated present-day human DNA contamination in the 17 remains using an approach described previously[73]: using 81,347 sites where B_Mbuti-4 (HGDP00456)[111] carries at least one derived allele, and calculate the proportion of these sites where a French individual (HGDP00521) or *Chagyrskaya 8* also carries the derived allele.

For *Chagyrskaya 8* the proportion is 0.00878 and for the French individual it is 0.468. The contamination c is then calculated as a linear combination of these two numbers, given the amount of shared sites p in a Chagyrskaya or an Okladnikov remain:

$$c \times 0.468 + (1 - c) \times 0.00878 = p$$

Thus, the contamination estimate is:

$$c = \frac{p - 0.00878}{0.468 - 0.00878}$$

The results are shown in **SIDataset1 - Nuclear capture tab** for each library with 95% binomial confidence intervals. This approach was also used for evaluation of initial shotgun screening (**SIDataset1 - Initial shotgun tab**). Note that, due to low coverage, the modern human contamination estimates are very noisy and at times negative.

For the curated dataset we include all reads from a library if the contamination estimate is around 3-4%. If the contamination estimate for all reads in the library is higher we only use deaminated reads if the contamination estimate for the deaminated reads is around 3-4%. Our decision is a trade-off between retaining as much data as possible and keeping the overall contamination rate for each remain low. In some cases we might include libraries for a remain that has slightly higher contamination estimates if that library has a majority of the reads.

Contamination from non-human sources

In order to check for other mammalian sources of contamination than present-day human DNA contamination we focused on the sites that were fixed-derived in the four high coverage archaic genomes and ancestral in all 504 Africans. We do not know if these sites are truly fixed or just found in high frequency in Neanderthals and Denisovans since we only have four high quality genomes of archaic individuals.

If there is substantial contamination either from present-day human DNA or another source there should be an enrichment in ancestral alleles at these sites since they are only fixed derived in the archaics. We show the results below in **SI Table 5.3**.

We find that *Chagyrskaya 9* has more ancestral variants than the other samples (20 times more than predicted from the present-day human contamination). We also see evidence of non-human sources of DNA contamination for *Chagyrskaya 6, Chagyrskaya 6_14* and *Chagyrskaya 63* - however, here the number of ancestral alleles is increased only between 5 and 10 fold. We do not observe a large increase in the mitochondrial sequence coverage for these remains however.

Given that the mitochondrial genome of *Chagyrskya 9* shows evidence for contamination with hyena DNA, (**SI Figure 3.1**) it is possible that a hyena could also be the source of

non-human contamination among the nuclear DNA sequences recovered from this remain.

Individual name	remain name(s)	n reads Covering 11,047 pos	non-Neanderthal contamination (%)	modern human contamination (%)
Chagyrskaya A	Chagyrskaya 01	1,919	1.56 (1.09-2.21)	1.97 (1.67-2.27)
Chagyrskaya B	Chagyrskaya 02	116,367	0.49 (0.44-0.52)	1.34 (1.31-1.37)
Chagyrskaya C	Chagyrskaya 06	1,605	1.0 (0.61-1.6)	0.10 (0.03-0.17)
	Chagyrskaya 14	183	2.19 (0.85-5.48)	2.58 (1.46-3.7)
	Chagyrskaya 06_14	1,732	0.98 (0.61-1.57)	0.13 (0.05-0.21)
Chagyrskaya D	Chagyrskaya 07	52,339	0.65 (0.58-0.72)	0.52 (0.34-0.69)
Chagyrskaya E	Chagyrskaya 09	387	9.04 (6.57-12.32)	0.42 (0.1-0.73)
Chagyrskaya F	Chagyrskaya 12	82,605	0.45 (0.4-0.49)	1.65 (1.61-1.7)
Chagyrskaya G	Chagyrskaya 13	5,563	2.03 (1.68-2.41)	1.54 (1.39-1.7)
	Chagyrskaya 19	3,452	0.61 (0.4-0.92)	1.04 (0.88-1.2)
	Chagyrskaya 63	148	0.68 (0.12-3.7)	0.08 (0.0-0.31)
	Chagyrskaya 13_19_63	8,855	1.43 (1.21-1.7)	1.27 (1.16-1.38)
Chagyrskaya H	Chagyrskaya 17	2,525	0.67 (0.42-1.07)	0.81 (0.64-0.98)
Chagyrskaya I	Chagyrskaya 18	10,223	0.84 (0.69-1.04)	1.30 (1.2-1.41)
Chagyrskaya J	Chagyrskaya 20	34,695	1.47 (1.34-1.59)	2.05 (1.98-2.13)
Chagyrskaya K	Chagyrskaya 41	50,513	1.52 (1.41-1.62)	1.87 (1.81-1.92)
Chagyrskaya L	Chagyrskaya 60	15,842	0.74 (0.62-0.88)	0.68 (0.62-0.74)
Okladnikov A	Okladnikov 11	341	1.76 (0.8-3.75)	1.85 (1.15-2.54)
Okladnikov B	Okladnikov 15	1,442	2.29 (1.63-3.18)	3.17 (2.73-3.61)

SI Table 5.3. Number of reads overlapping the sites where four high coverage archaics all carry the derived allele and the proportion of ancestral alleles for all 17 remains. We also show the estimated present-day human DNA contamination calculated in the **SI 5 - Modern human contamination estimation** section. *Chagyrskaya 9* is highlighted in dark red because of the excess of reads carrying the ancestral allele. *Chagyrskaya 6, Chagyrskaya 63* and *Chagyrskaya 6_14* are highlighted in light red because they also show an excess of reads carrying the ancestral allele although not to the same extent as *Chagyrskaya 9*.

Curated data set

In the **SI Table 5.4** below, we show the curated dataset for all remains after merging the data from all of our libraries. In this curated dataset we only include reads that overlap the 643,472 positions left after removing overlapping probes and sites described previously.

Individual name	remain(s) name	contamination (%)	contamination 95% CI (%)	sites called	average coverage	n reads
Chagyrskaya A	Chagyrskaya 1	1.97	1.67-2.27	102,616	0.2	126,540
Chagyrskaya B	Chagyrskaya 2	1.34	1.31-1.37	554,098	12.34	7,887,452
Chagyrskaya C	Chagyrskaya 6	0.1	0.03-0.17	88,491	0.16	99,702
	Chagyrskaya 14	2.58	1.46-3.7	11,784	0.02	12,107
	Chagyrskaya 6_14	0.13	0.05-0.21	94,972	0.17	107,738
Chagyrskaya D	Chagyrskaya 7	0.52	0.34-0.69	632,835	4.94	3,175,358
Chagyrskaya E	Chagyrskaya 9	0.42	0.1-0.73	21,464	0.04	22,563
Chagyrskaya F	Chagyrskaya 12	1.65	1.61-1.7	541,924	8.34	5,367,074
Chagyrskaya G	Chagyrskaya 13	1.54	1.39-1.7	227,192	0.57	365,309
	Chagyrskaya 19	1.04	0.88-1.2	168,496	0.35	224,715
	Chagyrskaya 63	0.08	0.0-0.31	9,323	0.01	9,531
	Chagyrskaya 13_19_63	1.27	1.16-1.38	302,804	0.93	575,948
Chagyrskaya H	Chagyrskaya 17	0.81	0.64-0.98	127,601	0.25	161,728
Chagyrskaya I	Chagyrskaya 18	1.3	1.2-1.41	329,364	1.00	641,574
Chagyrskaya J	Chagyrskaya 20	2.05	1.98-2.13	483,160	3.52	2,263,191
Chagyrskaya K	Chagyrskaya 41	1.87	1.81-1.92	531,757	5.12	3,293,393
Chagyrskaya L	Chagyrskaya 60	0.68	0.62-0.74	385,267	1.58	1,013,756
Okladnikov A	Okladnikov 11	1.85	1.15-2.54	21,527	0.04	22,573
Okladnikov B	Okladnikov 15	3.17	2.73-3.61	80,019	0.14	89,833

SI Table 5.4. Summary statistics for the merged BAM files of the nuclear DNA capture data for the 17 remains, further grouped as individuals after identifying remains which stem from the same individual. We show present-day human DNA contamination estimates along with the number of sites/SNPs called (out of 643,472 sites), average coverage of the target sites and a number of reads overlapping those sites. Note that for the merged remains the numbers of called sites do not add up given that sometimes a same target site is covered by multiple reads stemming from different remains.

Sex determination

We calculated the average coverage for the autosomes and X chromosomes for all 17 remains using the curated dataset.

For females, who carry two copies of the X chromosome, the average coverage for the X chromosome and autosomes should be the same (a ratio of ~1). For males, who only carry one X chromosome, we would expect that the coverage of the X chromosome is half that of the autosomes (a ratio of ~0.5).

To calculate the average coverage we first excluded all SNPs that are found in both the African and Archaic subsets of our array because they have an increased coverage as described previously (see **SI Figure 5.3**). We also removed SNPs where the GC content in the flanking region is above 60% and below 30% - the mean GC content of the genome is 40%.

This left us with 11,886 sites on the X chromosome and 402,796 sites on the autosomes. We randomly sample 11,886 sites from the autosomes 100 times (while keeping the same sites for the X chromosome) for calculating our 95% confidence intervals.

We find that 6 of the remains belong to females, while the remaining 11 belong to males, see **SI Figure 5.7**.

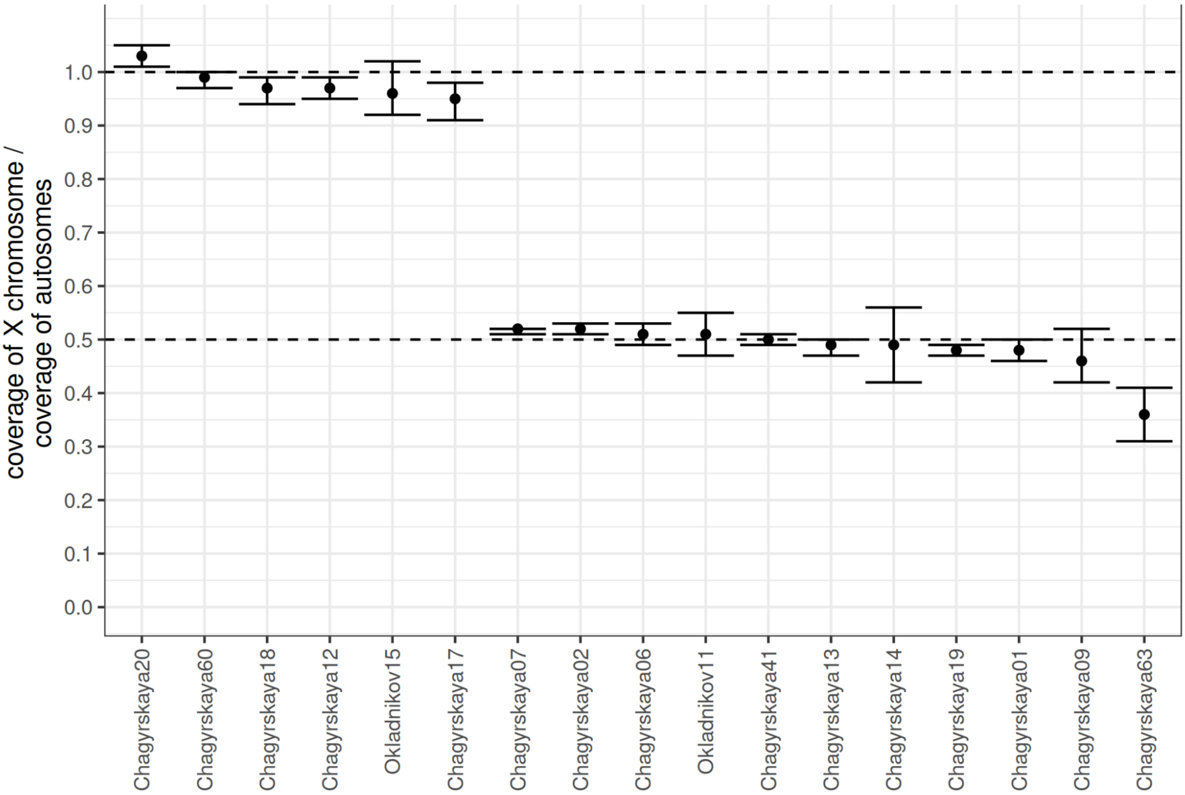

SI Figure 5.7. Ratio of the coverage of the X chromosome relative to the coverage of the autosomes. The dotted horizontal lines indicate the expected values if an individual is a female (expected ratio of ~1) or a male (expected ratio of ~0.5). The dot shows the mean estimate and the error bars show 95% confidence intervals using n=100 bootstrap replicates.

SI 6 Relationship to other archaic individuals

Chagyrskaya individuals are closer to Chagyrskaya 8

We use D-statistics[126,127] to investigate the relationship of the Chagyrskaya and Okladnikov Neanderthals to other high-coverage individuals. We find that both the individuals from Chagyrskaya and Okladnikov Caves are most closely related to the high-coverage *Chagyrskaya 8* individual, without evidence of substantial gene flow from any other Neanderthal population, as well as Denisovans or modern humans.

Data processing

We calculate F-statistics using admixtools[126], through the admixr R wrapper[128]. To prepare the input file in the eigenstrat-format, we start from BAM files (for the Chagyrskaya samples) and vcf-files (for the high-coverage individuals).

Data is prepared as:

1. Sites are restricted to the sites which have a total coverage less than 400 and do not overlap with other probes - (n = 643,472 - See **SI 5**).
2. We use the curated data files described in **SI 5 Curated data set**.
3. At each site, only one random read is sampled for each individual

We only report statistics of the form

D(X, Y; Test, Chimpanzee),

which tell us whether the test individual shares more alleles with the individual X vs. Y, while controlling for differences in sample ages. If D(X, Y; Test, Chimpanzee) < 0, the test individual is closer to Y than X; if D > 0, it is closer to X than Y, and if D = 0, then the test individual is either an outgroup, or a 50%/50% admixture between the two individuals/populations.

Comparison between High-Coverage Archaics

In **SI Figure 6.1**, we evaluate whether any of our remains show any significant relationship to the high-coverage *Vindija 33.19* Neanderthal from Croatia[76], by comparing it with the high-coverage *Chagyrskaya 8* individual[2], and by calculating D-statistics of the form D(*Vindija 33.19, Chagyrskaya 8*; Test, Chimpanzee). We find that all remains from Chagyrskaya and Okladnikov Caves share significantly more alleles with the *Chagyrskaya 8* individual. In contrast, many Late Western Eurasian Neanderthals, such as *Spy 94a*, *Goyet Q56-1* and *Mezmaiskaya 2* are significantly closer to *Vindija 33.19*, and earlier Neanderthals such as the *Altai*, *HST* and *Scladina*, as well as modern humans and Denisovans show no significant excess similarity to either of the two, consistent with them being outgroups to both *Vindija 33.19* and *Chagyrskaya 8*. For one remain, *Chagyrskaya 12*, we obtain much lower D-values (D=-0.7) than for most other remains (D~-0.4), consistent with *Chagyrskaya 12* and *Chagyrskaya 8* originating from the same individual.

Similarly, when calculating D-statistics of the form D(*Chagyrskaya 8*, Altai; Test, Chimpanzee) and D(*Chagyrskaya 8, Denisova 3*; Test, Chimpanzee), we do not observe any substantial differentiation between the Chagyrskaya remains, suggesting that the Chagyrskaya and Okladnikov Neanderthals are a largely homogeneous group with at most traces of gene flow from other archaic populations to the limits of our resolution.

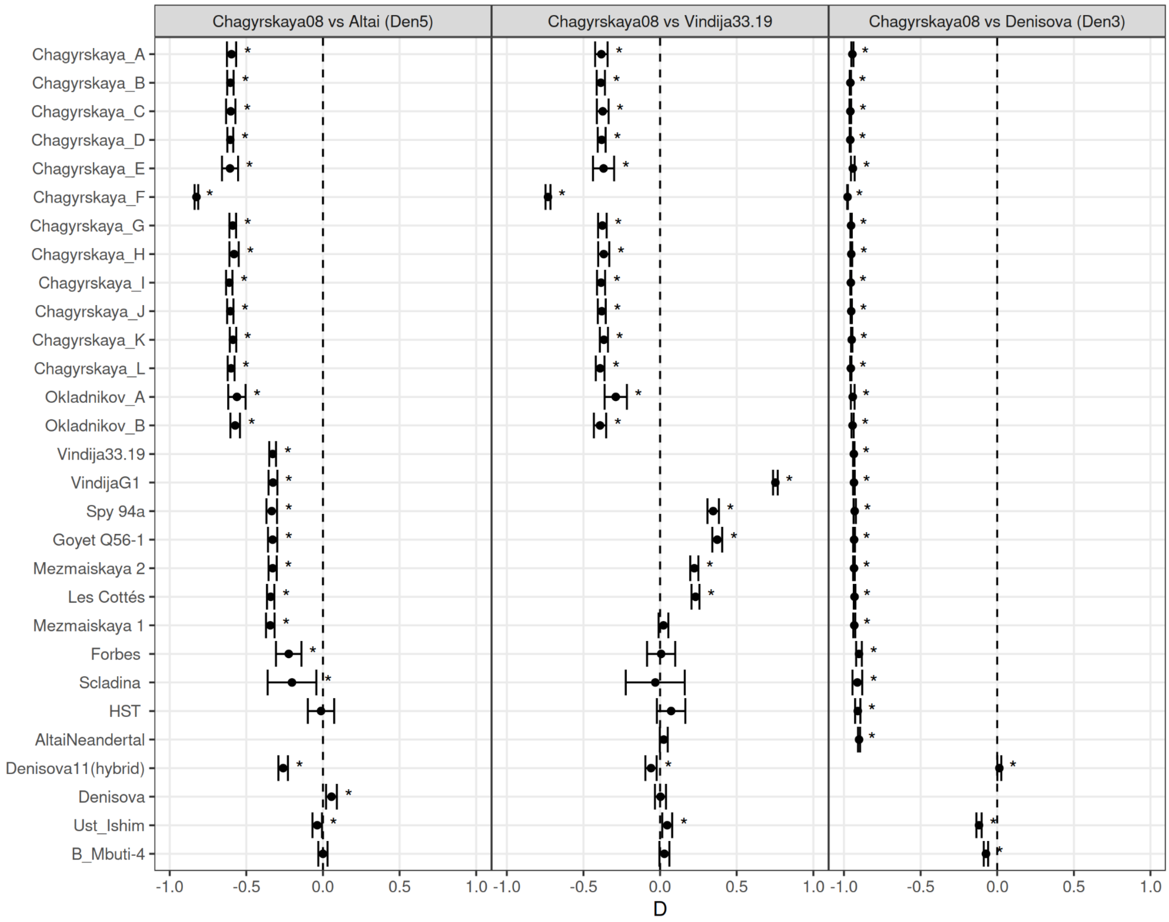

SI Figure 6.1. The center of the errorbar show the D-statistics of the form D((Altai/Vindija/Denisova), Chagyrskaya 08, Test, Chimpanzee). The point shows the D statistic and the error bars show the 95% confidence intervals calculated for 643,472 SNPs using a weighted block jackknife and a block size of 5 Mb. The points with a |Z-score| > 2 are denoted with an asterisk. The dotted vertical line is at D = 0.

Chagyrskaya 12 is the same individual as Chagyrskaya 08

To further test if any of the Chagyrskaya individuals were the same as any of the previously sequenced high coverage genomes we randomly sampled a read from each Chagyrskaya and Okladnikov individual and compared them to the genotyped high coverage individuals.

The expectation is that if an individual is the same as a high coverage individual we should see around 50% of reads supporting the alternative allele if the high coverage individual is heterozygous, around 0% of reads supporting the alternative site at sites where the high coverage individual is homozygous for the reference allele and around 100% of reads supporting the alternative site at sites where the high coverage individual is homozygous for the alternative allele. **SI Table 6.1** indicates that *Chagyrskaya 12* is the same individual as *Chagyrskaya 8*.

	**Altai Neanderthal (Den5)**			**Vindija 33.19**			**Chagyrskaya 8**			**Denisova 3 (Den3)**		
**remain**	0/0	0/1	1/1	0/0	0/1	1/1	0/0	0/1	1/1	0/0	0/1	1/1
Chagyrskaya 1	4.97	38.08	91.92	3.48	35.2	93.28	1.59	44.24	96.05	25.47	14.59	54.76
Chagyrskaya 2	4.47	36.89	93.07	3.04	33.24	94.21	1.17	43.77	96.92	23.14	12.02	58.63
Chagyrskaya 6	4.86	36.72	92.49	3.28	33.33	93.8	1.44	44.3	96.53	24.4	13.11	56.9
Chagyrskaya 7	4.44	36.5	93.15	2.96	33.54	94.23	1.19	43.36	96.91	22.75	11.71	59.25
Chagyrskaya 9	5.83	35.23	89.8	4.14	33.1	91	2.38	41.71	93.18	24.9	15.15	55.14
Chagyrskaya 63	4.66	37.58	92.85	3.36	36.79	94.14	1.48	46.86	96.2	25.96	15.11	53.28
Chagyrskaya 12	5.41	36.84	92.69	3.97	33.46	93.76	0.14	49.81	99.51	24.14	12.04	58.32
Chagyrskaya 13	4.74	37.55	92.2	3.26	33.4	93.34	1.46	42.75	96.04	24.64	14.12	55.82
Chagyrskaya 14	5.56	39.54	91.66	3.6	33.27	93.51	1.78	41.46	96.36	26.21	14.6	55.58
Chagyrskaya 17	4.79	37.98	92.8	3.25	34.73	93.82	1.4	44.06	96.66	24.74	14.12	56.72
Chagyrskaya 18	4.68	36.94	92.57	3.14	34.06	93.83	1.26	43.51	96.65	24.21	13.06	57.07
Chagyrskaya 19	4.57	37.15	92.72	3.16	33.76	93.98	1.28	42.34	96.71	24.56	13.78	56.61
Chagyrskaya 20	4.74	36.76	92.24	3.21	33.16	93.52	1.37	43.78	96.17	23.72	12.45	57.55
Chagyrskaya 41	4.67	36.53	92.14	3.22	32.99	93.29	1.42	43.25	95.91	23.27	12.24	58.06
Chagyrskaya 60	4.66	37.33	92.87	3.18	33.84	94.04	1.29	43.73	96.85	24.32	13.43	57.35
Okladnikov 11	4.78	37.6	91.67	3.4	31.93	93.09	1.66	41.53	95.67	24.87	14.64	55.38
Okladnikov 15	5	37.61	91.26	3.66	33.86	92.37	1.74	43.87	95.23	24.75	13.49	55.75

SI Table 6.1. Percentage of randomly sampled reads that carry the non-reference allele for each remain at a position where the high coverage genomes are either homozygous reference (0/0), heterozygous (0/1) or homozygous for the alternative allele (1/1). Comparison of *Chagyrskaya 12* to the high coverage *Chagyrskaya 8* is highlighted in green.

Denisovan introgression

We looked for the introgressed Denisovan fragments using admixfrog (version 0.6.1)[129]. We use the set of sites where the total coverage is less than 400 and the probes are non-overlapping (n = 643,472). We create a reference file using information from the high-coverage genomes by using the data from the Sub-Saharan Africans from SGDP[104] and the data of the three published high-coverage Neanderthal and Denisovan genomes, respectively. Recombination rate information is based on the African-American map[130]

We run admixfrog with the following parameters.

```
admixfrog --infile {input.infile} --ref {input.ref} -o {outname}
 --states NEA DEN
 --cont-id AFR
 --ll-tol 0.001
 --bin-size 20000
 --est-F --est-tau
 --e0 0.01
 --est-error
 --ancestral PAN
 --max-iter 250
 --filter-pos 50 --filter-map 0.000
```

We note that because we don't have full genome data, but only capture data many segments will be missed. We chose a cutoff of 0.2 cM. For *Chagyrskaya F* we only find one segment that is introgressed (the length is 0.02 cM so we remove it) vs the 12 segments found in *Chagyrskaya 8* which is high coverage (same individual as *Chagyrskaya F*).

name	total seq	total cM	segments	mean length	max length	min length
Chagyrskaya A (1)	746,464	1.5	1	746464	746464	746464
Chagyrskaya D (7)	561,629	0.58	4	140407	368586	21035
Chagyrskaya H (17)	419,510	0.3	2	209755	332214	87296
Chagyrskaya J (20)	428,600	0.48	2	214300	321592	107008
Chagyrskaya K (41)	580,715	0.38	2	290358	378145	202570

SI Table 6.2. Amount of sequence, which is more similar to the high coverage Denisovan than Neanderthal genomes for each of the analysed individuals. We only show individuals which have fragments longer than 0.2 cM. The remains originating from the same individual are shown in parentheses.

We first investigated the longest fragment which is found in *Chagyrskaya A* in **SI Figure 6.2** which has a length of 746 Kb**.**
We can calculate the probability that this fragment has been maintained in both Denisovans and Chagyrskaya Neanderthals since the two populations split from each other (as in[131]).
The split time of Denisovans and Neanderthals has been estimated to around 420,000 years ago[76] and if we assume that *Chagyrskaya A* lived ~60,000 years ago this means that the fragments must have been maintained for around 360,000 years (*t*). We also assume a constant recombination rate at 1.5×10−8 per base pair (bp) per generation (*r*) and a generation time of 29 years per generation[101].

The expected length of fragments maintained this long is (R code below):

```
L = 1/(r * t)
```

where L is the expected mean length of an ILS fragment in bp, r is the recombination rate and t is the time since the two populations (Denisovans and Neanderthals) last exchanged genetic material. Here, t is the time since the Denisovan and Neanderthal split and the sampling time of the *Chagyrskaya A* individual. The expected length distribution of ILS fragments is given by an exponential distribution with rate parameter rt. Given this expectation we can ask how likely it is to observe a fragment as long or longer than the one observed in *Chagyrskaya A* assuming fragments are caused by ILS (R code below):

```
pexp(length, rate= rt,lower.tail=F) < 4e-61
```

The probability that a fragment this long or longer has been maintained since the split between Neanderthals and Denisovans is smaller than 4e-61.

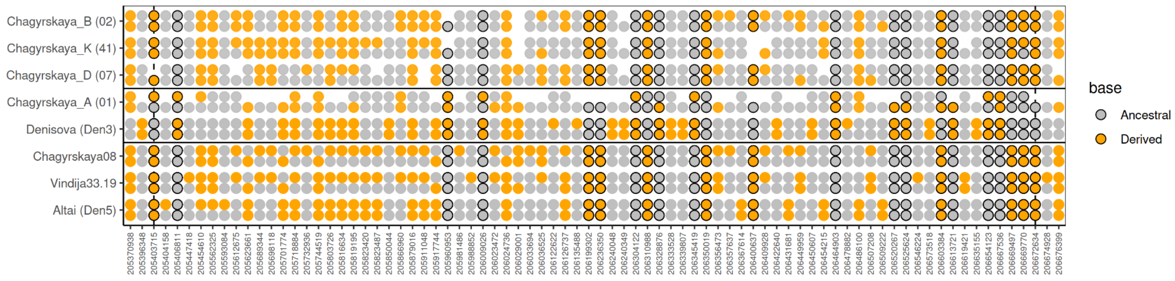

SI Figure 6.2. Putative Denisovan introgressed fragment is shown for *Chagyrskaya A* next to the *Denisova 3* genome in a box. Shown are the derived and ancestral alleles. Note that we sample two random reads from *Chagyrskaya A* at each position when the coverage is greater than 2, otherwise we sample 1 or 0 reads.

The maximum likelihood estimate for the admixture time can be found in the following way:

$$t_{admix} = \left(\frac{1}{n}\sum_{1}^{n}(n_i - 0.02\ cM)\right)^{-1} * 100 * 29$$

Here we calculate the admixture time (tadmix) using only fragments longer than 0.02 cM and convert it into years assuming 29 years/generation[101,129]. We show the results when combining all Denisova fragments in all Chagyrskayas **SI Table 6.3**.

Individual name	Segments	Time (years)	95% CI
All segments	11	30,673	±18,126

SI Table 6.3. Estimated admixture time and 95% CI based on all segments or on segments found in one individual.

We estimate that the admixture is 30,673±18,126 years ago which is similar to the admixture time of 24,300±14,100 years ago estimated from Denisovan fragments in *Chagyrskaya 8* in [129].

SI 7 Relatedness analysis

We used READ[132] to identify remains originating from the same or related individuals. READ estimates relatedness by comparing the pairwise differences between a pair of individuals to the median pairwise differences in a given population. An in-house python script was used to randomly sample an allele at all biallelic sites for each individual, and to generate the input file for READ. We applied READ with the default settings (median as normalization parameter, window size of 1Mb).

Sites used

We used the curated SNP set (**SI 5**) and only keep sites that are varying between the four high coverage archaic genomes (the ARC_var sites), and we removed sites which are only derived in the high coverage *Chagyrskaya 8* individual. Ascertaining sites in *Chagyrskaya 8* would make this remain, and remains from closely related individuals, appear more different from the other samples than it actually is. Lastly, we removed sites on the X chromosome. This left us with 250,785 sites.

Modern human contamination correction

We noticed that the pairwise differences between two individuals correlate with the combined contamination estimate of both individuals (p value=4.7e−11) - see **SI Figure 7.1**. In order to correct for this we introduced a correction term which is a linear combination of the pairwise differences between a modern human B_Mbuti-4 (HGDP00456)[111] and the *Chagyrskaya 8* individual, and the true pairwise difference:

$$\pi_{obs} = \pi_{true} \cdot (1 - c) + \pi_{Chag08-human} c$$

Here c is the combined contamination estimate from (**SI 5 - Modern human contamination estimate**).

For example, if we are comparing *Chagyrskaya 1* to *Chagyrskaya 2*, c would be (1.95% + 1.34% = 3.31%). By combining the contamination estimates, we assume that either *Chagyrskaya 1* or *Chagyrskaya 2*, but not both, contribute a contaminant read at any position. The pairwise difference between *Chagyrskaya 8* and a modern human ($\pi_{Chag08-human}$) is 0.378. We show the uncorrected and corrected pairwise distances as a function of c in **SI Figure 7.1**.

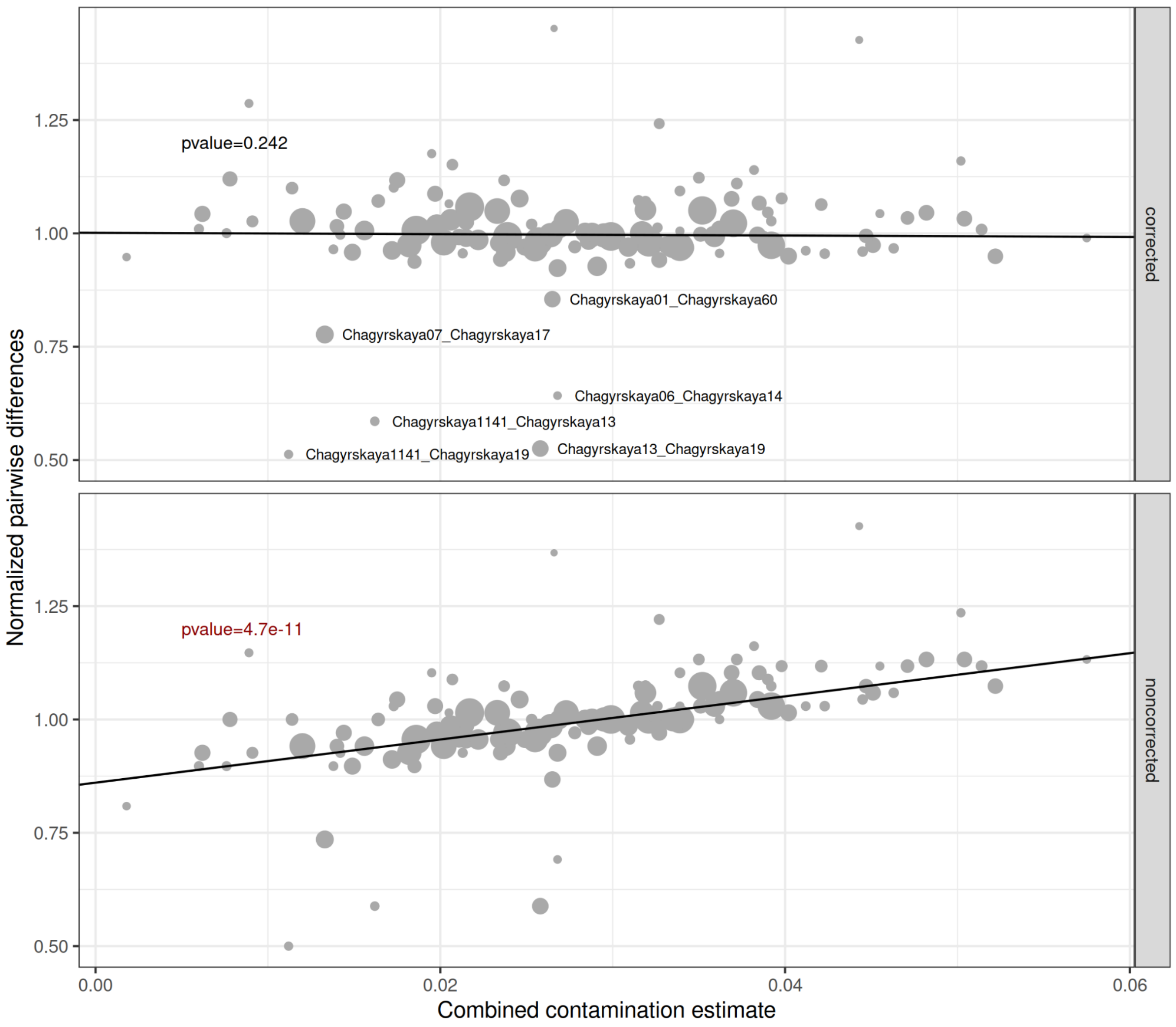

SI Figure 7.1. Normalized pairwise differences as a function of the joint contamination for all pairs of remains (c1 + c2) before and after correcting for contamination - the size of each dot is the number of overlapping sites between remains. The p-value (T-test, 2-sided, correcting for multiple testing not applicable) of the slope is indicated.

Nonhuman contamination correction

Note that *Chagyrskaya 9* is not shown in **SI Figure 7.1.** That is due to its large nonhuman contamination component (see **SI 5 - Contamination from non-human sources**). In order to analyze *Chagyrskaya 9* we introduce another way of normalizing the pairwise differences. For each remain we normalize the pairwise difference by the median of all comparisons which includes that remain. The idea is that the nonhuman contamination in *Chagyrskaya 9* for instance will affect all pairwise comparisons in the same way.

When we use all individuals the normalization factor is 0.059 and when the normalization factors for all Chagyrskaya remains is 0.056-0.062 except for *Chagyrskaya 9* where it is 0.08.

We used the cutoffs suggested in READ[132] for both normalization methods:

Identical individuals/monozygotic twins (normalized dist<0.625)
First degree relationship (0.625<normalized dist<0.815)
Second degree relationship (0.815<normalized dist<0.90)

In **SI Table 7.1** are the degree of relationships we identify between remains (up to second degree) with the two different normalization methods. Note that only comparisons including *Chagyrskaya 9* change the degree of relationship.

We also show this as a heatmap in figure **SI Figure 7.2** both before merging and after merging specimens from the same individuals.

			**Normalize using all remains**			**Normalize for each remain**		
**remain 1**	**remain 2**	**Overlap**	p_0	**95% CI Range**	**Relationship**	p_0	**95% CI Range**	**Relationship**
Chagyrskaya 63	Chagyrskaya 19	1,788	0.513	0.342-0.65	Identical	0.474	0.36-0.62	Identical
Chagyrskaya 13	Chagyrskaya 19	38,429	0.525	0.462-0.548	Identical	0.513	0.47-0.56	Identical
Chagyrskaya 63	Chagyrskaya 13	2,463	0.586	0.445-0.753	Identical	0.556	0.42-0.68	Identical
Chagyrskaya 12	Chagyrskaya 8	214,921	0.467	0.408-0.459	Identical	0.430	0.38-0.44	Identical
Chagyrskaya 6	Chagyrskaya 14	1,001	0.642	0.342-0.787	Identical/ 1st degree	0.623	0.39-0.84	Identical/ 1st degree
Chagyrskaya 9	Chagyrskaya 7	8,763	0.87	0.771-0.989	2nd degree	0.638	0.58-0.70	Identical/ 1st degree
Chagyrskaya 7	Chagyrskaya 17	50,455	0.777	0.719-0.821	1st degree	0.789	0.75-0.82	1st degree
Chagyrskaya 1	Chagyrskaya 60	37,694	0.855	0.77-0.907	2nd degree	0.828	0.77-0.86	2nd degree
Chagyrskaya 9	Chagyrskaya 17	2,995	1.17	1.039-1.408	>2nd degree	0.850	0.75-0.95	2nd degree?
Chagyrskaya 9	Chagyrskaya 13	5,306	1.121	0.938-1.223	>2nd degree	0.838	0.76-0.925	2nd degree?

SI Table 7.1. We show remains which are inferred to be up to second degree related using different normalization methods.

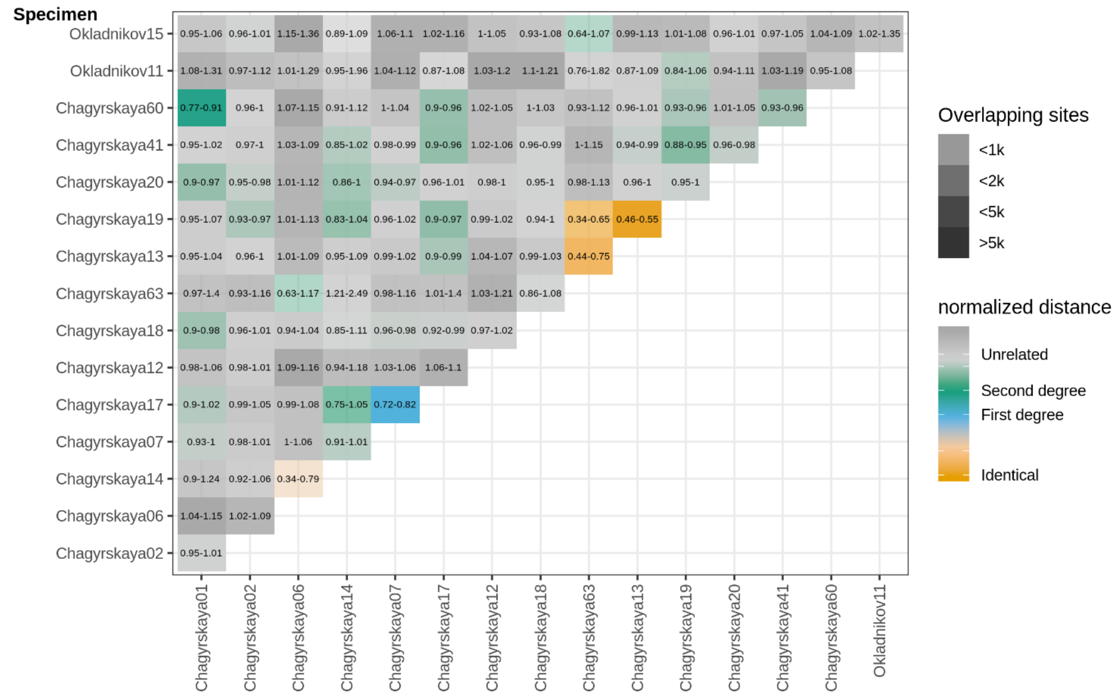

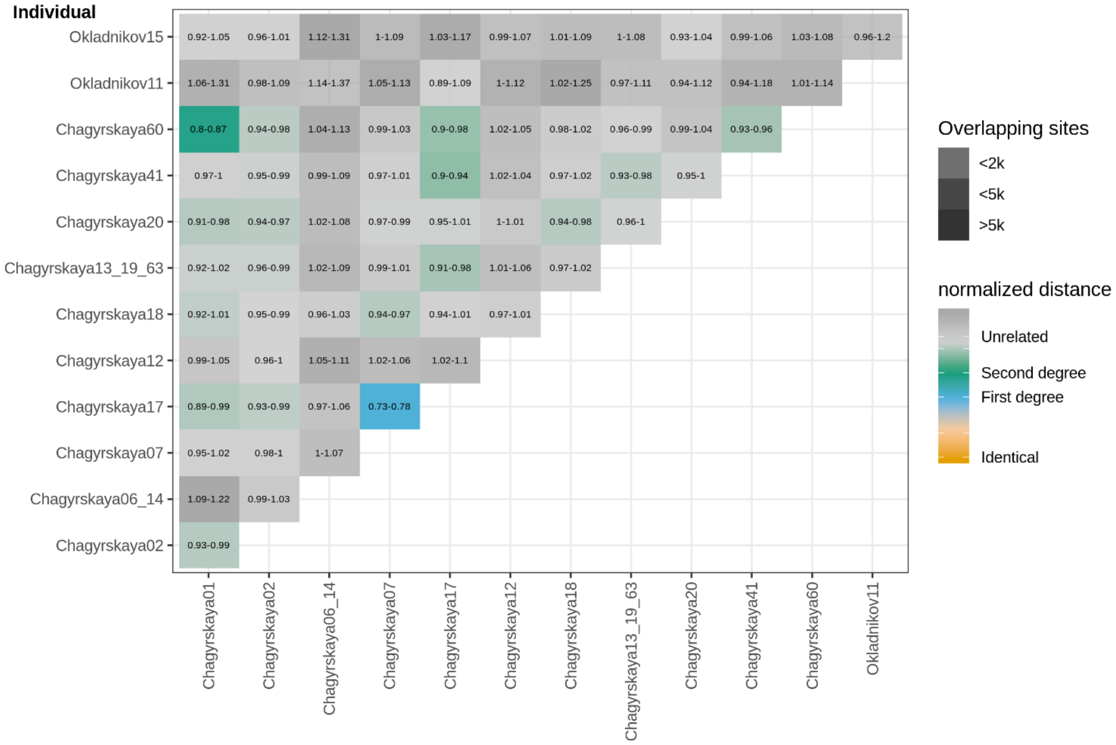

SI Figure 7.2. Heatman showing normalized pairwise difference for each pair of specimens/Individuals. The boxes are colored according to normalized pairwise difference and the intensity of the color corresponds to the amount of overlapping sites - the text in the boxes show the 95% confidence interval estimated from 100 bootstrap iterations. The bottom panel is a repetition of the analysis with fossils assigned to the same individuals merged.

Identical individuals
We note that the group of remains originating from the same individual (*Chagyrskaya 13-19-63*) all share the same mitochondrial heteroplasmies at positions 3961 and 303-318 (in a coordinate system of *Chagyrskaya 8* mtDNA) in similar frequencies (**SI 3 - Heteroplasmies**).

The same is true for the second group of remains (*Chagyrskaya 6-14*), which share a heteroplasmy at position 545 (in a coordinate system of *Chagyrskaya 8* mtDNA). In addition to this, *Chagyrskaya 14* is a permanent incisor that fits into the *Chagskaya 6* mandible (see **SI 1 - remains**). The higher observed pairwise difference between these two remains can be explained with the elevated levels of the non-human contamination detected in *Chagyrskaya 6* (see **SI 5 - Other forms of contamination**). Taken together, these three lines of evidence strongly support the conclusion that *Chagyrskaya 6* and *Chagyrskaya 14* belonged to the same individual.

Another line of evidence to support these observations comes from *Chagyrskaya 8*, the remain which was previously sequenced to high coverage (27X)[2]. In total, 21 different libraries were constructed for this remain, thus enabling us to compare all 210 pairwise combinations of these libraries, amounting to a distribution of pairwise differences between libraries originating from a single individual. The expectation is that the pairwise differences for all putative remains belonging to the same individuals would fall within this distribution. Indeed, we observe that all pairwise comparisons for the groups of remains *Chagyrskaya 13-19-63* and *Chagyrskaya 6-14* fall within the 95% percentile of the distribution calculated using *Chagyrskaya 8* libraries - see **SI Figure 7.3**.

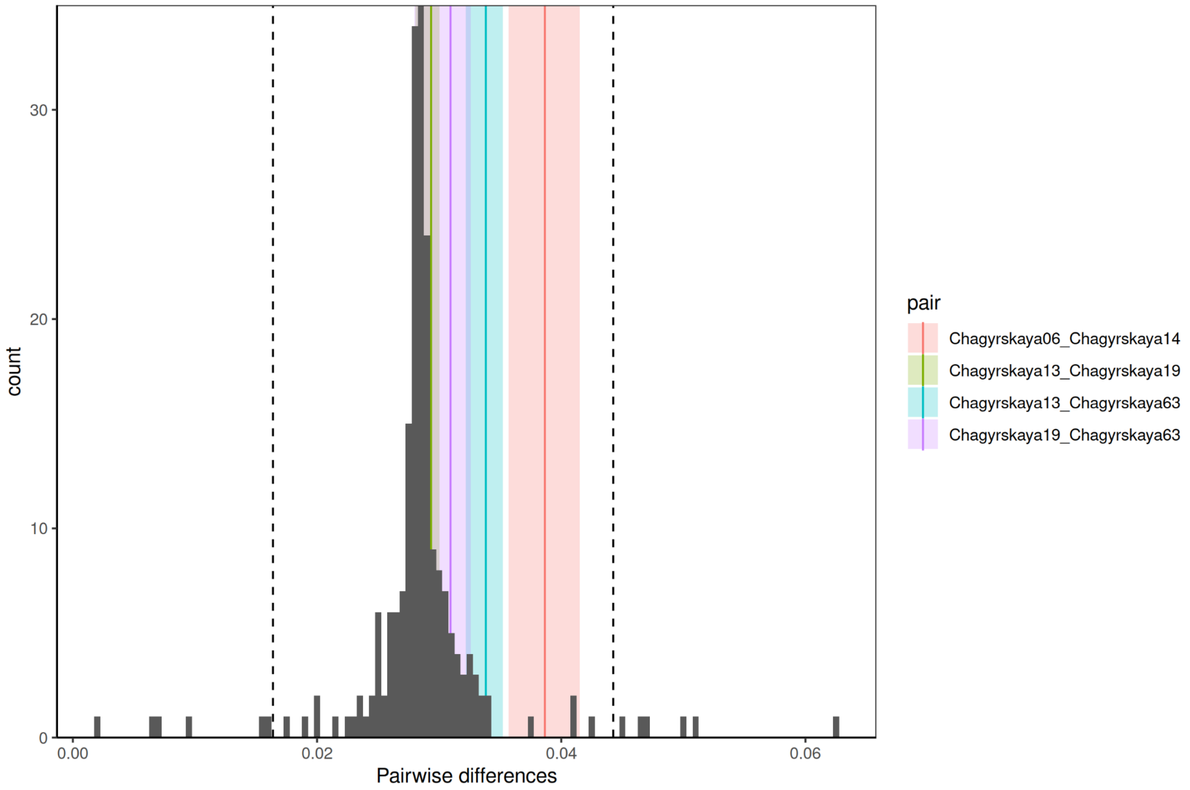

SI Figure 7.3. Histogram of the pairwise differences generated from 21 libraries (n = 210 combinations) from *Chagyrskaya 8*. The dotted vertical lines encompass 95% of the distribution. The mean pairwise difference between identical remain pairs are shown (pairs are colored) as a vertical line. The filled bars are confidence intervals based on blocked jackknife where one chromosome is removed in each iteration.

We also find that *Chagyrskaya 8* and *Chagyrskaya 12* are from the same individual which is also supported by their genotypes (not just pairwise difference) in **SI Table 6.1.**

For *Chagyrskaya 9* and *Chagyrskaya 7* we cannot be sure of their relationship. They share a heteroplasmy with a frequency of the derived allele at 42 - 54% for *Chagyrskaya 7* and 23-30% for *Chagyrskaya 9*. This would indicate that they are not the same individual **SI Table 3.2** - but contamination estimate for *Chagyrskaya 9* is high and this would decrease the frequency of the derived allele and could explain why they are different. We note that READ estimates a first degree relationship between *Chagyrskaya 7* and *Chagyrskaya 17* but a second degree relationship between *Chagyrskaya 9* and *Chagyrskaya 17*. This would indicate that *Chagyrskaya 7* and *Chagyrskaya 9* are different individuals but again the confidence intervals are wide **SI Table 7.1**. For now we say *Chagyrskaya 9* is either from a distinct individual (*Chagyrskaya E*) or the same as *Chagyrskaya D*. In order not to analyze the same individual twice we remove from all further analysis.

We thus identify at least 13 (not sure about *Chagyrskaya 9/7*) unique individuals based on our 17 remains as shown in **SI Table 7.2**. We designate individuals with letters and remains with numbers.

**Individual**	**remain**
Chagyrskaya A	Chagyrskaya 1
Chagyrskaya B	Chagyrskaya 2
Chagyrskaya C	Chagyrskaya 6
	Chagyrskaya 14
Chagyrskaya D	Chagyrskaya 7
Chagyrskaya E?	Chagyrskaya 9
Chagyrskaya F	Chagyrskaya 8
	Chagyrskaya 12
Chagyrskaya G	Chagyrskaya 13
	Chagyrskaya 19
	Chagyrskaya 63
Chagyrskaya H	Chagyrskaya 17
Chagyrskaya I	Chagyrskaya 18
Chagyrskaya J	Chagyrskaya 20
Chagyrskaya K	Chagyrskaya 41
Chagyrskaya L	Chagyrskaya 60
Okladnikov A	Okladnikov 11
Okladnikov B	Okladnikov 15

SI Table 7.2. remains likely originating from the same individuals.

First degree relationship

Chagyrskaya H (female) and *Chagyrskaya D* (male) are classified as being first degree related, thus pointing to a father-daughter, brother-sister or mother-son relationship. Therefore, we looked at the mitochondrial sequences of these two individuals. If they are different, that would rule out a brother-sister relationship because the same mitochondrial genome would be inherited from the mother, as well as a mother-son relationship because the son would inherit the same mitochondrial genome from the mother.

The mitochondrial genomes of these two individuals are different (4 differences) which only leaves a father-daughter relationship (**SI 3 - Phylogenetic relationship**).

Furthermore, if it is a father-daughter pair, we would expect the pairwise differences across each autosome to be the same (normalized pairwise difference of 0.75) and the X chromosome to have a normalized pairwise difference of 0.5. The daughter will inherit one of her X chromosomes from the father and the other one from the mother, hence when we randomly sample an allele we would expect to see a matching read half of the time. We show that this is indeed also the case in **SI Figure 7.4**.

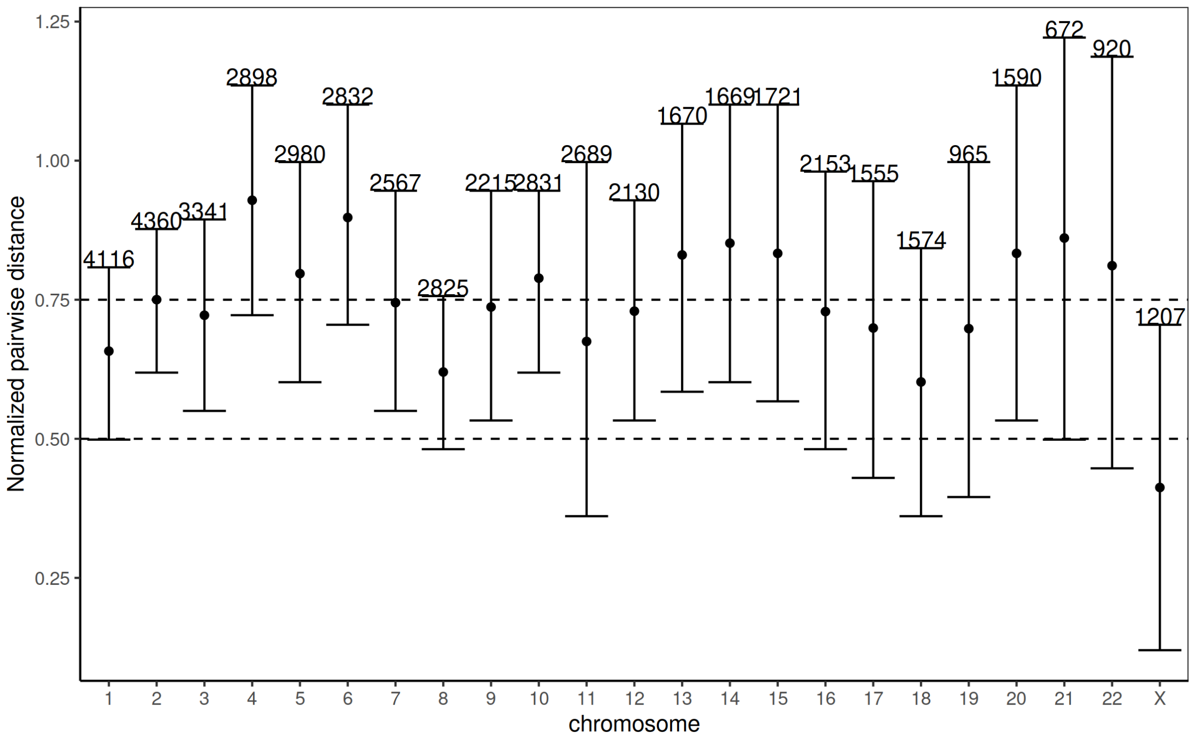

SI Figure 7.4. Mean pairwise differences and 95% confidence intervals across all chromosomes using 100 bootstrap replicates for each chromosome (number of sites for each chromosome is shown above the error bar). HLA region on chromosome 6 is excluded. The horizontal bars indicate 0.75 pairwise difference (first degree) and 0.5 pairwise differences (identical).

Relationship between Chagyrskaya and Okladnikov remains

We wanted to test if individuals from the Chagyrskaya Cave are more similar to other individuals from the Chagyrskaya Cave than they are to individuals from the Okladnikov Cave.
To do this we selected all 11 individuals from Chagyrskaya Cave (*Chagyrskaya E* removed) and two individuals from Okladnikov Cave and computed within-cave and between-cave pairwise distances, sampling random reads at each position.

We excluded pairwise-comparisons between related individuals, which is the father-daughter pair (*Chagyrskaya D* and *Chagyrskaya H*) and the second degree relationship between *Chagyrskaya A* and *Chagyrskaya L.*
We compute distances as the normalized distance and take contamination into account (**SI 7 - Relatedness analysis**).

Due to the low coverage of both Okladnikov individuals we could not get an accurate estimate of the diversity within Okladnikov Cave. We could however compare the between-cave diversity to the within cave diversity. We show the results in **SI table 7.3.**
We find that individuals from Chagyrskaya Cave are more related to each other than Chagyrskaya and Okladnikov individuals (Wilcoxon rank-sum test p-value = 8.6e−05).

		**Pairwise distance**			**normalized pairwise distance**		
**between within**	**n comparisons**	**difference**	**min 95% CI**	**max 95% CI**	**difference**	**min 95% CI**	**max 95% CI**
Chag_Chag	55	0.0591	0.0584	0.0598	1	0.99	1.01
Chag_Oklad	22	0.0628	0.061	0.0645	1.06	1.04	1.09
Oklad_Oklad	1	0.0674	-	-	1.14	0.9	1.38

SI Table 7.3. Pairwise differences and the normalized pairwise differences for within and between cave comparisons.

Relationship between contemporaneous and non-contemporaneous individuals

We find two sets of individuals which might be contemporaneous and a set of individuals which does not seem to be related - Note that *Chagyrskaya E* is removed.

Set A
Chagyrskaya D and *Chagyrskaya H* (father-daughter)
Chagyrskaya D and *Chagyrskaya C* (shares a heteroplasmy)

Set B
Chagyrskaya A and *Chagyrskaya L* (second degree relationship)

Set C
Chagyrskaya B, Chagyrskaya F, Chagyrskaya G, Chagyrskaya I, Chagyrskaya J and *Chagyrskaya K*

We do not know if the sets all represent the same occupation or if they represent separate occupations by genetically similar groups. In order to test if the set A, set B and set C are contemporaneous with each other, we calculated pairwise differences within the unrelated set C and compared it to the differences between set A/C and set B/C and tested if they were significantly different using a Wilcoxon rank-sum test.
We did not find any significant differences between the set A/C compared to set C/C or between set B/C compared to set C/C (**SI Table 7.4**).

**First set**	**Second set**	**Mean pairwise diff and 95% CI (First group)**	**Mean pairwise diff and 95% CI (Second group)**	**n**	**P-value**
Set A vs Set C	Within Set C	0.999 [0.609-1.389]	0.984 [0.651-1.317]	15	0.26
Set B vs Set C	Within Set C	0.98 [0.564-1.396]	0.984 [0.651-1.317]	12	0.53

SI Table 7.4. Comparisons within and between set A/C and set B/C. For each set we show the mean pairwise differences along with the 95% confidence intervals.

SI 8 Mitochondrial and Y chromosome diversity

The genetic diversity on uniparental markers such as mitochondrial and Y chromosomal DNA is affected by sex-biased demographic processes. In a scenario with random mating the coalescence time of maternally inherited mtDNA and paternally inherited Y chromosomes are expected to both be ¼ of the coalescence time of the autosomes (expected coalescence time for the autosomes is $2N_e$, and 1/2 $N_e/2$ for the mtDNA and Y chromosome). However, if males have a skewed offspring distribution where only a few males contribute to the next generation, we would expect Y-diversity to be reduced. The converse case of skewed female offspring distribution would result in lower mtDNA diversity. In addition, sex-biased migration patterns may produce similar results; if females more often move to live with the mans group (male philopatry) we expect elevated between-population diversity and reduced within-population diversity on the Y chromosome, and we would expect the same pattern on mtDNA in communities where females move less frequenly.
It has been estimated that (male philopatry) exists to some extent in around 70% of human societies and in addition to this the genetic differences between Y chromosomes are typically higher than between mitochondrial genomes[133,134].
Apes too show substantial variation in their social organization. In Sumatran Orangutans females mainly show philopatric tendencies while males migrate[135,136]. Other studies argue that both males and females show philopatric tendencies in Bornean Orangutans[137] while other studies find evidence of male migration and female philopatry[136,138,139].
Gorillas communities are typically polygynous[140] with one-male groups[141] with female dispersal[142–144] although male dispersal can also occur[145].
Chimpanzee communities show male philopatric tendencies while females migrate[146–148] although this does not seem to be the case in some Western chimpanzee communities where there is both female and male migration[149,150].
Bonobo communities also show male philopatric tendencies while females migrate[151,152]

Data sets used

To compare the Y chromosome and mitochondrial DNA diversity we downloaded full mitochondrial genomes and Y chromosome sequences from present-day and early modern humans, as well as mitochondrial sequences from other Neanderthal groups and great apes.

Present-day humans from HGDP
We downloaded full mitochondrial sequences (n=1,058) from Genbank with accession numbers KF450814-KF451871[153] and Y chromosome sequences (n=603)[154].

Early modern humans
We downloaded ancient individuals with available mitochondrial sequences. We selected individuals from the same locations and where the calibrated radiocarbon dates differed less than 2,500 years.

Groups where Y chromosome and mitochondria data is available

- 4 individuals from Bacho Kiro Cave[155]
- 4 individuals from Sunghir[156]

Groups where only mitochondrial data is available

- 5 individuals from Goyet[84,157]
- 4 individuals from Vestonice[157]
- 3 individuals from Paglicci[157]
- 3 individuals from Iboussieres[84]

Neanderthals

Neanderthals (no Y chromosome data available) - all mitochondrial sequences taken from.

- 2 individuals from Feldhofer[68]
- 4 individuals from Vindija [67,68,71,76]
- 7 individuals from Goyet[72,77]
- 11 individuals from Chagyrskaya cave (*Chagyrskaya E* is removed)

Great apes

We also downloaded 115 mitochondrial genomes[118,158,159] for great apes and Y chromosome genotypes for 39 great apes[118].

We note that the great ape data is very heterogeneous in the sense that some subspecies have very few sampled individuals and in some subspecies a majority of individuals are not wildborn (**SI Table 8.1**).

Common name	Species name	mtDNA		Y chromosomes	
		n	Wildborn	n	Wildborn
Eastern lowland gorilla	Gorilla beringei graueri	7	89.0%	2	100.0%
Western lowland gorilla	Gorilla gorilla gorilla	43	71.0%	7	85.7%
Mountain gorilla	Gorilla beringei beringei	8	100.0%	3	100.0%
Bonobo	Pan paniscus	16	75.0%	4	100.0%
Nigerian chimpanzee	Pan troglodytes ellioti	6	100.0%	4	100.0%
Eastern chimpanzee	Pan troglodytes schweinfurthii	7	100.0%	3	100.0%
Central chimpanzee	Pan troglodytes troglodytes	6	100.0%	2	100.0%
Western chimpanzee	Pan troglodytes verus	8	38.0%	8	28.6%
Sumatran orangutan	Pongo abelii	6	50.0%	4	25.0%
Bornean orangutan	Pongo pygmaeus	8	33.0%	2	50.0%

SI Table 8.1. We show the number of mtDNA sequences (which corresponds to the number of individuals), Y chromosome sequences (which corresponds to the number of males) and the proportion of these that are wildborn vs captive born.

Mutation rates

To incorporate uncertainty in the mutation rate estimates we gathered previously published studies which estimate the mutation rate for the Y chromosome (n=9) and mtDNA (n=8). The rates are shown in **SI Table 8.2**.

**Point estimate (mutations per bp per year)**	**95 % CI range**	**Short name**	**Citation link**
mtDNA - values are 1e-8			
1.30	0.42 - 4.1	Rebolledo-Jaramillo et al 2014	[160]
1.67	1.37 - 1.97	Soares et al 2009	[161]
1.89	1.15 - 2.2	Zaidi et al 2019	[162]
1.92	1.16 - 2.68	Fu et al 2013	[63]
2.23	1.71 - 2.75	Rieux et al 2014	[163]
2.40	1.7 - 3.2	Brotherton et al 2013	[164]
2.53	1.8 - 3.2	Fu et al 2014	[86]
2.74	2.44 - 3.01	Posth et al 2016	[84]
Y chromosome - values are 1e-10			
5.3	None provided	Francalacci et al 2013	[165]
6.1	4.3 - 7	Mendez et al 2013	[106]
7.3	6.27 - 8.46	Petr et al 2020	[10]
7.4	6.3 - 9.5	Karmin et al 2015	[105]
7.6	6.7 - 8.6	Fu et al 2014	[86]
7.8	None provided	Balanovsky et al 2015	[166]
8.2	7.2 - 9.2	Poznik et al 2013	[167]
8.3	7.26 - 9.63	Helgason et al 2015	[168]
10.3	3.07 - 24.1	Xue et al 2009	[169]

SI Table 8.2. Mutation rate estimates for mitochondria (mtDNA) and the Y chromosome in humans - sorted from smallest to largest. The mtDNA rates are multiplied by e8 for clarity and the Y chromosome values are multiplied by e10. Note the mtDNA values are for the entire mitochondria (including D-loop).

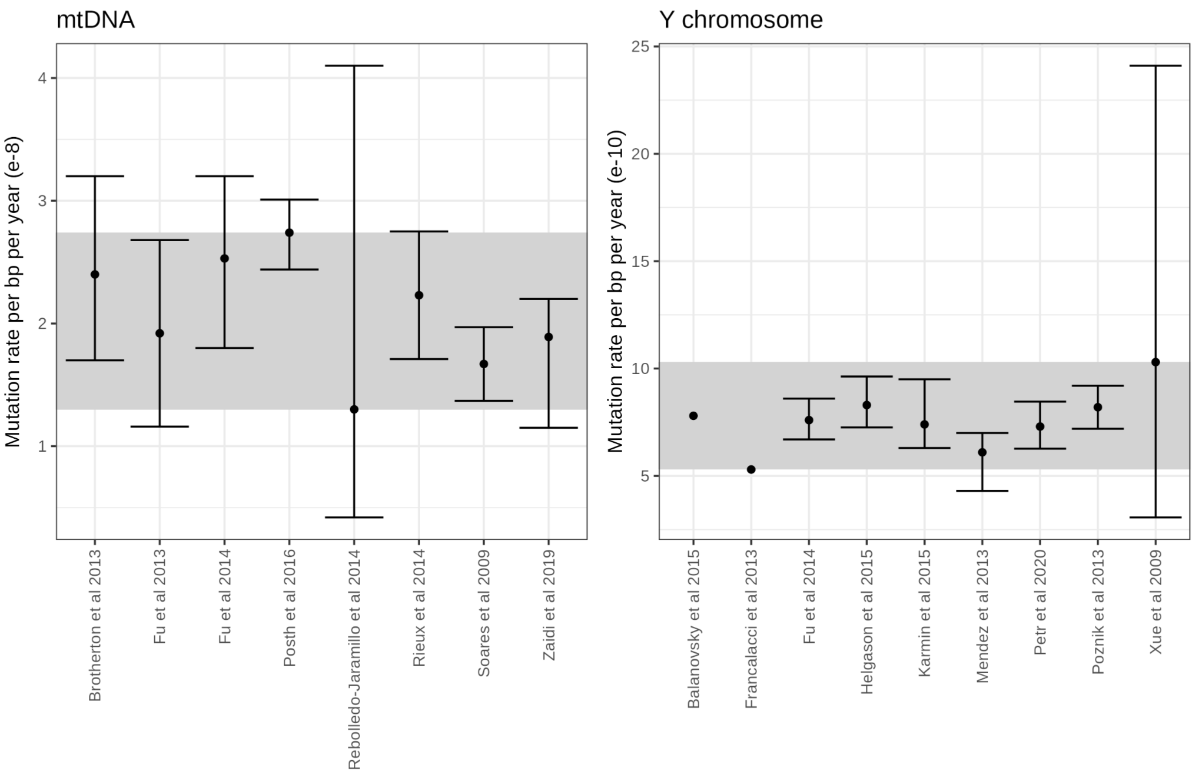

SI Figure 8.1. The various previous mutation rate estimates for the mtDNA and Y chromosome - note the error bars represent 95% confidence intervals calculated in the respective publications. The grey area indicates the values we uniformly sample from.

When we perform simulations we uniformly pick mutation rates that are between the minimum and maximum point estimates for the mtDNA and Y chromosome respectively - the grey area in **SI Figure 8.1**. For the mtDNA the range of values we pick from is from 1.3e-8 to 2.74e-8 mutations per bp per year and for the Y chromosome the values are from 5.3e-10 to 10.3e-10 mutations per bp per year. When estimating coalescence times (**SI Table 8.3**) we also incorporate the uncertainty in mutation rate.

Generation time

For both the mtDNA and the Y chromosome, we assume a generation time of 29 years[101] for humans and Neanderthals even though the relative generation time of males and females might differ between modern humans and Neanderthals[170]. For chimpanzees and bonobos we use a generation time of 24 years, and for gorillas and orangutans we use a generation time of 20 years[118].

Mitochondrial and Y chromosome diversity

In **SI Table 8.3** we show the coalescence time estimates for the Y chromosome and mtDNA. The Y chromosome average coalescence time is 446 years . and the 95% confidence interval is 113 - 1,116 years when taking the variation in mutation rate into account. The values are similar when only using transversions **SI Table 8.3**.

The average coalescence time for the mitochondria is 4,348 years and the 95% confidence interval is 2,043 - 6,196 years when taking the variation in mutation rate into account.

	comparisons	mean diff (diff per bp)	Average coalescence time in years	minimum coalescence time in years	maximum coalescence time in years	P value when comparing Y chromosome to mtDNA (wilcoxon)
Y chrom (6 Chagyrskaya individuals)						
Ts + Tv	15	7.08E-07	446	113	1116	4.1e-05
Tv	15	3.12E-07	519	64	1428	8.6e-05
Y chrom (4 Chagyrskaya individuals with > 1.7 fold coverage)						
Ts + Tv	6	1.51E-06	952	399	2075	0.022
Tv	6	4.69E-07	780	121	2098	0.015
mtDNA (11 Chagyrskaya individuals)						
Ts + Tv	55	1.37E-04	4348	2043	6196	-

SI Table 8.3. The average coalescence time in years for the Y chromosome and mtDNA. For the Y chromosome we calculate the mean coalescence time both for transversions and transitions (Ts + Tv) and for transversions only (Tv). For the Y chromosome transversions rate we divide the total mutation rate by (1+1.64) as there are 1.64 more transitions than transversions. For the mtDNA all differences between individuals are transitions.

We tested if the coalescence time for the Y chromosome and mtDNA is significantly different from each other using a Wilcoxon signed rank test for all four comparisons (all variations of the Y chromosome coalescence time from **SI Table 8.3**). All are significantly different from each other.

Assuming a generation time of 29 years[101] we can calculate the effective population sizes of men and women which is 15 for men (446/29) and 150 for women (4348/29).
We can also use the effective population size of men and women to estimate the effective size of the population using the standard equation:

$$N_e = \frac{4N_f N_m}{N_f + N_m}$$

Using the mean effective size estimates of N_m = 15 for males and N_f = 150 for females yields an overall effective population size of 54 individuals - which is similar to what has been

estimated from the nuclear genome of *Chagyrskaya 8* (Ne is less than 50 individuals when migration rate is > 1%)[2].

Comparing absolute diversity levels in Chagyrskaya to other populations

The mean coalescence time estimates for all populations are displayed in **SI Figure 8.2**. We tested if the mitochondrial and Y chromosomal diversity was different for our Chagyrskaya individuals compared to other populations using Wilcoxon signed rank test.

Mt diversity

We find that the mitochondrial diversity in the Chagyrskaya individuals is not significantly different from the Surui population (a Native American population) (p=0.55, n=8 mitochondria), the Sunghir individuals (p = 0.17, n=4 mitochondria) and the Okladnikov individuals (p = 0.87, n=3 mitochondria) (**SI Figure 8.2 A**). For all other mitochondrial chromosome comparisons the Chagyrskayas have significantly lower diversity.

Y chromosome diversity

We also find that the Y chromosomal diversity in the Chagyrskaya individuals is not significantly different from the one in the Surui population (p = 0.54, n=4 y chromosomes). Furthermore the Y chromosomal diversity is also not significantly different from the Y chromosome diversity in the mountain gorilla population (p = 0.76, n=3 y chromosomes). For all other Y chromosome comparisons the Chagyrskayas have significantly lower diversity. (**SI Figure 8.2 A**)

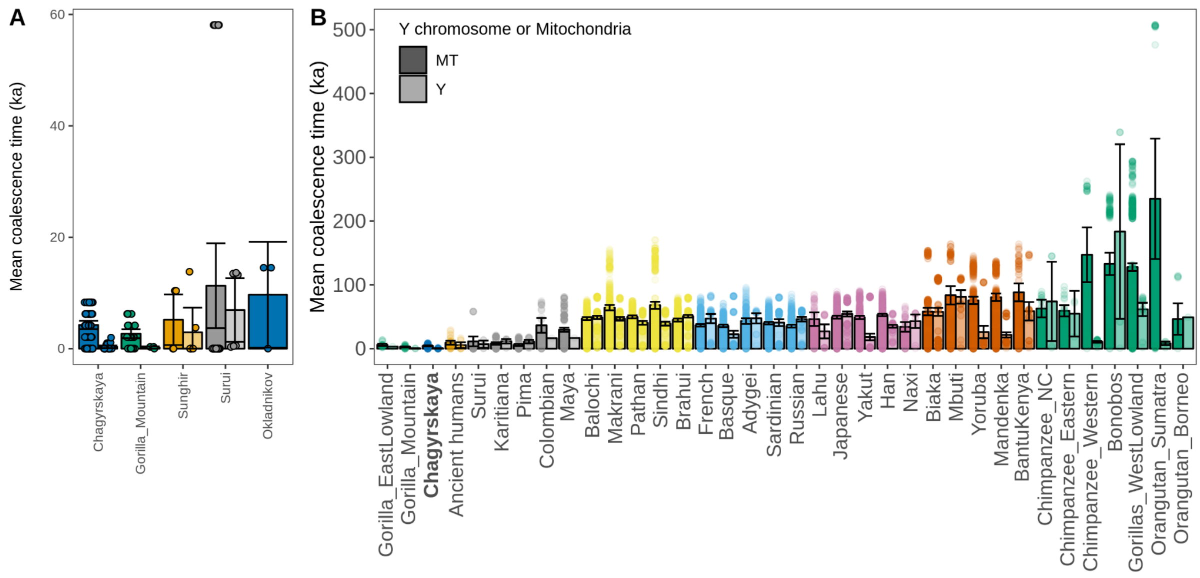

SI Figure 8.2. Mean coalescence time for the mitochondrial (MT) DNA and Y chromosome (Y). The points show all pairwise coalescence times for Y and MT. The black bar in the box plots denote the mean while errorbar denote the 95% confidence intervals. Sample sizes are for calculating confidence intervals: **Neanderthal/Denisovan:** Chagyrskaya(mt=55, y=15), Okladnikov(mt=3, y=0). **Early modern humans:** Sunghir(mt=6, y=6) and all Ancient modern humans which combined in **B** is (mt=28, y=9). **Great apes:** Mountain Gorilla(mt=28, y=3),

EastLowland Gorilla(mt=21, y=1), Chimpanzee_NC(mt=15, y=6), Chimpanzee_Eastern(mt=21, y=3), Chimpanzee_Western(mt=28, y=28), Bonobos(mt=120, y=6), Gorillas_WestLowland(mt=903, y=21), Orangutan_Sumatra(mt=28, y=6), Orangutan_Borneo(mt=15, y=1). **America:** Surui(mt=36, y=6), Karitiana(mt=78, y=10), Pima(mt=91, y=21), Colombian(mt=28, y=1), Maya(mt=231, y=1). **Central South Asia:** Balochi(mt=300, y=276), Makrani(mt=325, y=190), Pathan(mt=300, y=171), Sindhi(mt=300, y=190), Brahui(mt=325, y=300). **Europe:** French(mt=406, y=55), Basque(mt=276, y=105), Adygei(mt=136, y=21), Sardinian(mt=406, y=105), Russian(mt=325, y=120). **East Asia:** Lahu(mt=36, y=21), Japanese(mt=378, y=171), Yakut(mt=325, y=153), Han(mt=561, y=105), Naxi(mt=36, y=15). **Africa:** Biaka(mt=253, y=231), Mbuti(mt=91, y=45), Yoruba(mt=253, y=55), Mandenka(mt=253, y=91), BantuKenya(mt=66, y=45). **A** Shows the populations (Sunghir, Surui and Okladnikov) where the MT coalescence time is not significantly different from the Chagyrskaya population coalescence time and the populations (Mountain gorilla and Sunghir) where the Y chromosome coalescence time is not significantly different compared to the Chagyrskaya population. **B** shows all populations.

Comparing ratio of mt to Y chromosome diversity

We estimated the mean differences between the Y chromosome coalescence time and mitochondria coalescence time and normalized this by the mitochondria coalescence time. This measurement will be negative when Y chromosome coalescence time is low and greater than zero when the mitochondria coalescence time is less than the Y chromosome coalescence time. We calculated 95% confidence intervals for this measurement by iteratively removing one Y chromosome and one Mitochondria sequence from the data. The results are shown in **SI Figure 8.3**. The Chagyrskaya Neanderthals have an extremely low value of -0.89 for this statistic, which is lower than all human populations surveyed and only comparable to three great ape populations: the Western Chimpanzee and Sumatran Orangutan, and the mountain gorilla. We note that less than 50% of the individuals in Western Chimpanzee and Sumatran Orangutan are wildborn and thus this ratio might not reflect their social organization.

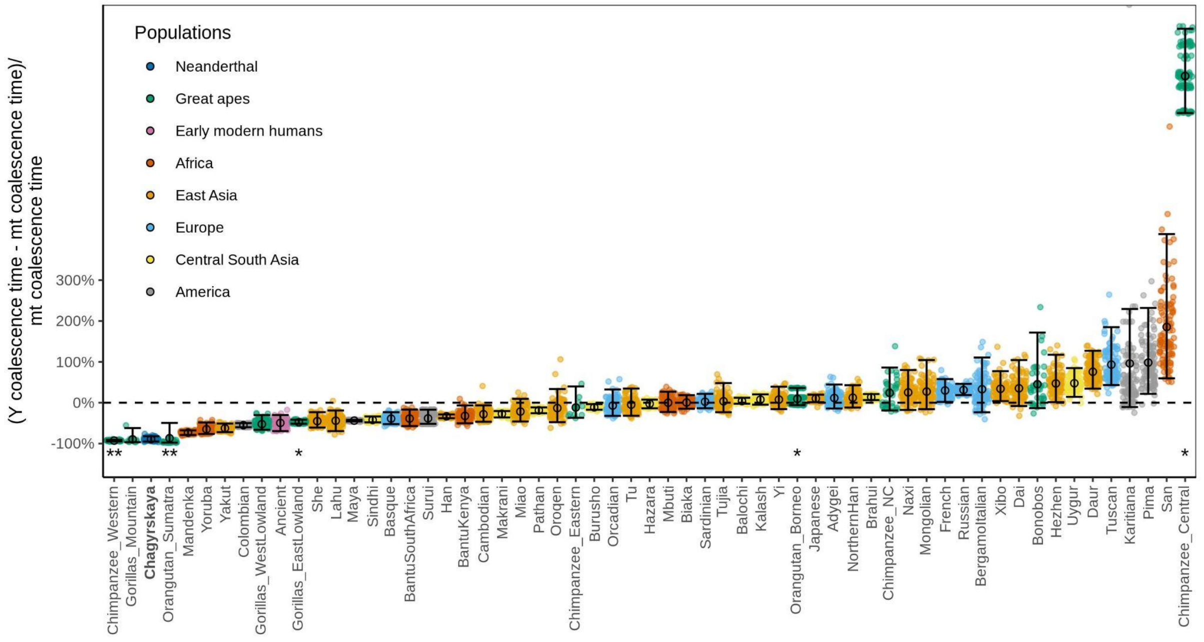

SI Figure 8.3. Black circles are mean estimates for each population and error bars are the corresponding 95% confidence intervals using 100 bootstrap iterations. Each colored point is a bootstrap iteration. Negative values denote lower Y-chromosome diversity than mitochondrial (mt) DNA diversity. We highlight great ape species where *) only data for two Y chromosomes exist and our estimate for Y chromosome coalescence time is one data point **) More than half of individuals are born in captivity. Those species are not shown in **Extended Figure 6**.

Causes for difference in Y chromosome and mt coalescence time

We consider four primary reasons for the observed low Y-chromosome coalescence time:

i) Neutral processes:
The differences in coalescence times might simply be due to genetic drift. Here we only vary the number of individuals in a deme.

ii) Difference in generation time:
We are assuming the same generation time for men and women of 29 years per generation[101]. Since all of the mutation rate estimates from **SI Table 8.2** are in mutations per bp per year this might affect our coalescence time estimates. In this section we change the generation time.

iii) High variance in male reproductive success:
Few men will father most of the children which will lead to high male offspring variance and thus to lower male coalescence time.

iv) Higher female migration rates:
In societies with male philopatry, women primarily move between communities, which results in higher female genetic coalescence time than in males.

Simulation parameters

Below we investigate the effect of the different scenarios using simulations. For all scenarios we perform 1,000 iterations of Wright-Fisher simulations. Following[2], we simulated 20 communities (called demes in[2]) that exchange migrants at rate *m* for 300 generations before they all coalesce into one population with an effective population size of 500 haploid individuals (250 males and 250 females).
For testing the scenarios we simulate all combinations of the following parameters for testing scenarios:

Y chromosome and mtDNA sample size, length and mutation rate
Y chromosome

- mutation rate is uniformly sampled between 1.3e-8 to 2.74e-8 mutations per bp per year (**SI Figure 8.1**)
- genome length is 3e6 base pairs (average number of comparisons we could make with 6 Y chromosomes)
- number of samples is 6

Mitochondria

- mutation rate is uniformly sampled between 5.3e-10 to 10.3e-10 mutations per bp per year (**SI Figure 8.1**)

- genome length is 15,446 base pairs (excluding D-loop)
- number of samples is 11

Population size
$N_f = N_m$ for the values (10, 15, 20, 25, 50, 75, 100, 125, 150, 175 and 200 haploid individuals). Note the total number of men and women are always the same.

Male skew parameters

For modeling skewed offspring distributions, we set up the following scenario: Divide the males into three groups - males in group A will have many children, group B will have one child each and males in group C will not have any children.

We vary the amount of males in group A from 0%-20% and also vary the total proportion of children coming from these males from 20%-100%. As an example, let's assume a population size of 150 males:

Say the 10% of males are in group A (15 men in total) and these men have 33% of the children (50 children in total): That means that the average male in group A has 3.3 children. Since we assume the same number of men in each generation there are 100 children left - that means that 100 males will be in group B and have one child each. In the end 35 males are in group C and will not have any children.

We simulate scenarios where the proportion of Males in group A is (0, 2, 4, 6, 8, 10 , 12, 14, 16, 18 and 20%) and the proportion of children fathered by males from group A (20, 25, 30, 35, 40, 45, 50, 55, 60, 65, 70, 75, 80, 85, 90, 95 and 100%).

Migration rates parameters
We vary the female migration rate in the range (0, 0.1, 0.2, 0.4, 0.6, 0.8, 1,2,4,6,8,10,20,40, 60, 80 and 100%). A female migration rate of 100% means that all females who have children in a given generation will be migrants from other communities.

Generation time differences
We set the values for the simulated male generation time to (20, 25, 30, 35, 40, 45, 50, 55 and 60 years) and the female generation time to (20, 25, 30, 35, 40 and 45 years).

Comparing Scenarios
For each scenario we calculate the percentage of simulations that “fit” our data. For a simulation to fit we require that the coalescence time for the simulated Y chromosome and simulated mtDNA fall within the 95% confidence interval of the values we observe in the Chagyrskaya population and the ratio statistic (y coalescence time - mt coalescence time)/mt coalescence time also fall within the 95% confidence interval of the value we observe in the Chagyrskaya population. The proportion of fitting simulations can be thought of as an approximation of the likelihood P(summary statistics | simulation parameters). If we assume a uniform prior over all simulated scenarios, a higher likelihood represents a better fit to the data.

Simulation results

We show the 5 best fitting scenarios when only considering one factor such as generation time, migration rate and male reproductive skew and the combinations of female migration rate and male reproductive skew in **SI Table 8.4**. We simulated a total of 103,037 scenarios.

**Population size (males + females)**	**Female Generation time (Years)**	**Male Generation time (Years)**	**Female Migration rate (%)**	**Males in group A**	**Children having fathers from group A**	**Simulations that fit (%)**
Scenario i) Varying population size						
70	29	29	-	-	-	1.0
90	29	29	-	-	-	1.0
80	29	29	-	-	-	0.9
60	29	29	-	-	-	0.8
100	29	29	-	-	-	0.6
Scenario ii) Varying generation time						
100	40	20	-	-	-	1.7
100	40	25	-	-	-	1.7
60	45	25	-	-	-	1.5
80	40	20	-	-	-	1.4
60	35	25	-	-	-	1.4
Scenario iii) Varying Male skew parameters						
300	29	29	-	3/150 males	67/150 children	6.0
300	29	29	-	18/150 males	150/150 children	6.0
300	29	29	-	12/150 males	135/150 children	5.8
180	29	29	-	3/90 males	45/90 children	5.7
250	29	29	-	5/125 males	68/125 children	5.7
Scenario iv) Varying female migration rate						
20	29	29	60	-	-	7.6
30	29	29	90	-	-	7.4
30	29	29	100	-	-	6.4
30	29	29	80	-	-	5.9
20	29	29	80	-	-	5.6
Scenario iii) + iv) Combination of increased female migration rate and male reproductive skew						
30	29	29	90	2/15 males	3/15 children	5.6
180	29	29	0.3	12/90 males	81/90 children	5.5
30	29	29	90	2/15 males	3/15 children	5.5
40	29	29	100	1/20 males	4/20 children	5.3
30	29	29	90	3/15 males	6/15 children	5.3

SI Table 8.4. We list the 5 best fitting scenarios for each four scenarios and for when we combine scenarios with male skew iii) and female migration rate iv). We show the female migration rate, and the amount of men in group A out of the total males and how many females have children with males from group A. Grey fields indicate the values were fixed to either 29 [101] for generation times or 0 for migration and male reproductive skew parameters.

To compare the different models more formally we calculated the Akaike information criterion (AIC) for each model. We show the results for the best fitting model for each scenario in **SI Table 8.5**.

**Scenario**	**Percent of simulations that fit**	**Log likelihood**	**degrees of freedom**	**AIC**	**Relative likelihood of model (compared to best model)**
iv) Varying female migration rate	0.076	-1.119	2	6.238	1
i) Varying population size	0.010	-2.000	1	7.000	0.68
iii) Varying Male skew parameters	0.060	-1.222	3	7.444	0.55
iii) + iv) Combination of increased female migration rate and male reproductive skew	0.056	-1.252	4	8.504	0.32
ii) Varying generation time	0.017	-1.770	3	8.539	0.32

SI Table 8.5. For each scenario we show the percent of simulations that fit, the log likelihood, degrees of freedom and Akaike information criterion (AIC) for the best fitting set of parameters.

The best fitting model (scenario iv) based on the AIC criterion is the model with varying female migration rate. The second best model (scenario i)) is $e^{(6.238 - 7.0)/2} = 0.68$ times as probable as the first model to minimize the information loss.

However, in this calculation, no prior information on Neanderthal community sizes is taken into account - that is, we assume that all community sizes are equally likely.
Previous estimates of Neanderthal community sizes, based on fossilized footprints, are 10-13 individuals[171] and 3-31 individuals[172]. Previous estimates from sleeping areas at various sites estimate 12-25 individuals[173]. The remains of at least 13 contemporaneous individuals[174] have been found in Spain. The community size of the Neanderthals occupying Chagyrskaya has been estimated to be less than 60 individuals[2] based on runs of homozygosity in the nuclear genome. This analysis also concluded that migration between communities was needed to explain the data. In **SI FIgure 8.4** we show the relative likelihood for the best fitting scenarios from scenario i), iii) and iv) stratified by community size. In the community size range estimated from the archeological literature our data is best explained by female migration.

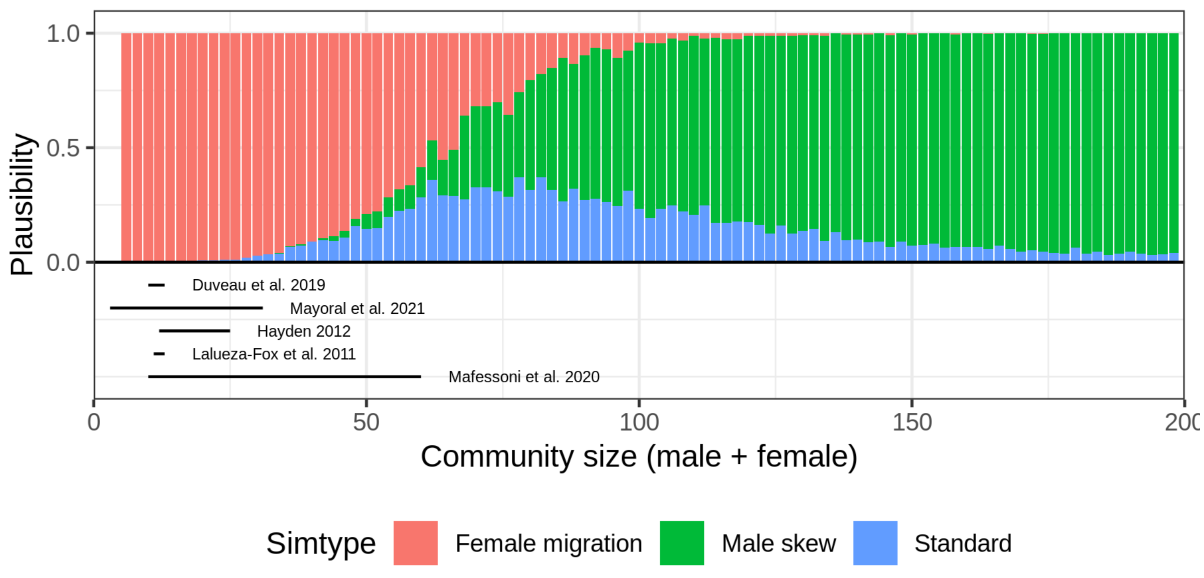

SI Figure 8.4. The plausibility for the best fitting models from scenarios i), iii) and iv) for a given community size. The plausibility is the probability of a given scenario scaled by the sum of the probabilities from the other scenarios. Below we show previous estimates of Neanderthal community size based on fossilized footprints, sleeping areas and runs of homozygosity.

In the best fitting simulated scenarios between 60-100% of females in each generation are migrants from other communities. The size of each community would be around 10 male and 10 females for a total of 20 individuals which would be consistent with previous estimates (**SI FIgure 8.4**).

The model with female migration rate **iv)** fits better than the scenario with only male reproductive skew **iii)** and scenarios with both female migration rate and male reproductive skew **iii)** + **iv).** In addition, the discovery of mtDNA sequences from Okladnikov Cave which are more similar to those of Chagyrskaya individuals (**SI Table 3.9**) and some mtDNA sequences recovered from the Chagyrskaya Cave sediments which are similar to *Okladnikov 2*, which is consistent with female migration.

Explaining our data with male reproductive skew (scenario **iii)**) alone would require a community size of 300 individuals which would far greater than other estimates of Neanderthal community sizes and also would not be consistent with the patterns observed in the nuclear genome. Furthermore the community sizes for scenario **i)** and **ii)** would also be much greater than previous estimates.

We conclude that female migration/male philopatry are able to recapture the observed diversity patterns.

SI 9 Diversity and runs of homozygosity

Runs of homozygosity

We called runs of homozygosity (ROH) using admixfrog[129] (version 0.6.1). We use the set of sites where the total coverage is less than 400x and the probes are uniquely overlapping the target sites (n = 643,472), using the same reference file as used for the analysis to detect Denisovan ancestry (see **SI 6 - Denisovan introgression**).
We run admixfrog with the following parameters.

```
admixfrog --infile {input.infile} --ref {input.ref} -o {outname}
" --states CHA "
" --cont-id AFR "
" --ll-tol 0.001 "
" --bin-size 20000 "
" --est-F --est-tau --freq-F 3 "
" --freq-contamination 3 "
" --e0 0.001 "
" --ancestral PAN "
" --est-inbreeding "
" --run-penalty 0.4"
" --max-iter 250"
" --est-error "
" --filter-pos 50 --filter-map 0.000"
```

Admixfrog calculates a posterior decoding of local ancestry Z at genomic position *i* as $P(O, G, Z) = P(Z_0 \prod_i P(O_i|G_i)P(G_i|Z_i)P(Z_i|Z_{i-1})$ using a Hidden Markov Model, where O_i designates the read data at SNP *i*, and the latent variable G_i is the genotype of the SNP at that position. Using only the *Chagyrskaya 8* [2] high coverage genome as a reference for the states, the local ancestry Z allows just two states; an outbred state that allows heterozygotes using Hardy-Weinberg equilibrium with frequencies 2p(1-p)f, where p is the estimated allele frequency in the reference Neanderthal; and an inbred state where heterozygous genotypes are not allowed.

We validate this approach empirically on the ascertained data using a downsampling experiment. For this purpose, we compare ROH calls from *Chagyrskaya 12* to the high coverage *Chagyrskaya 8*[2], as these two remains originated from *Chagyrskaya F*. Results are given in **SI Table 9.1**. We note that as coverage decreases we especially lose the ability to identify shorter fragments.

	long (>10cM)			Short (2.5-10cM)		
	Total length cM	fragments	reduction	Total length cM	fragments	reduction
Chagyrskaya 8	334	17	0.0%	431	98	0.0%
Chagyrskaya12 (8X)	314	14	6.0%	323	70	25.1%
Chagyrskaya12 (4X)	274	13	18.0%	249	48	42.2%
Chagyrskaya12 (2X)	256	14	23.4%	90.2	16	79.1%

SI Table 9.1. Reduction in ROH identification rate when decreasing coverage.

We also show the genomic location of all segments in **SI Figure 9.1**. We note however that our new approach tends to join shorter ROH segments and thus might be overestimating the amount of ROH > 10 cM.

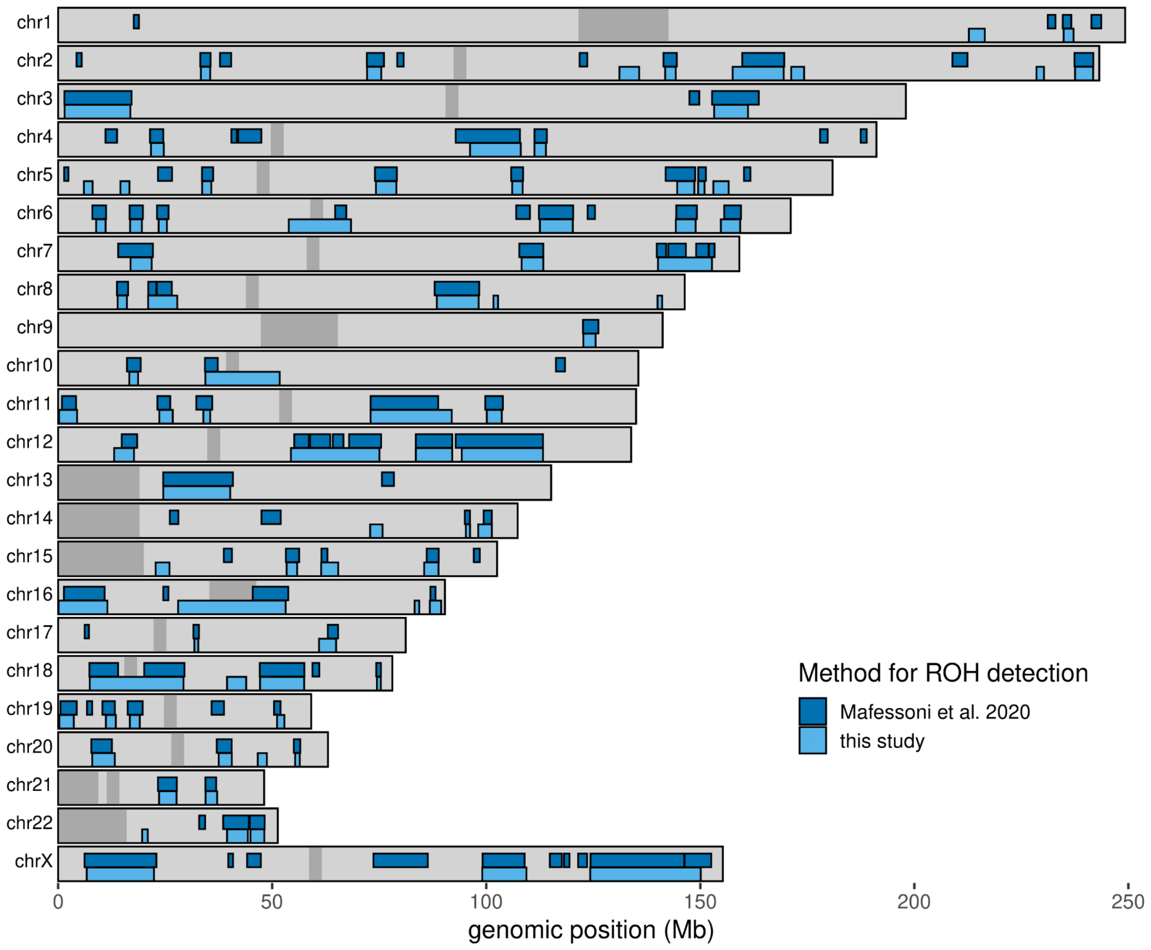

SI Figure 9.1. Comparing genomic locations of all ROH longer than 2.5cM for *Chagyrskaya F (Chagyrskaya 8)* from two different studies. We used all reads

We only report individuals that have ROH longer than 2.5 cM in **SI Table 9.2**. We also show the genomic locations of ROH longer than 10 cM in **SI Figure 9.2**.

		**long (> 10 cM)**			**short (2.5 - 10 cM)**			**Both**		
**Individual**	**Coverage**	**total cM**	**% of genome**	**n**	**total cM**	**% of genome**	**n**	**total cM**	**% of genome**	**n**
Chagyrskaya B (2)	12.34	308	9.1%	19	712	20.9%	144	1,020	29.96%	163
Chagyrskaya F (12)	8.34	314	9.2%	14	323	9.5%	70	637	18.74%	84
Chagyrskaya K (41)	5.12	367	10.8%	22	626	18.4%	136	993	29.20%	158
Chagyrskaya D (7)	4.94	255	7.5%	18	609	17.9%	136	864	25.40%	154
Chagyrskaya J (20)	3.52	508	14.9%	27	624	18.4%	133	1,132	33.30%	160
Chagyrskaya L (60)	1.58	250	7.4%	18	487	14.3%	108	737	21.65%	126
Chagyrskaya I (18)	1.00	120	3.5%	8	450	13.2%	102	570	16.73%	110
Chagyrskaya G (13,19,63)	0.93	55.4	1.6%	4	447	13.1%	107	502.4	14.73%	111

SI Table 9.2. Total length (in cM) and number of ROH fragments sorted by coverage of the individual.

We combined our estimates with ROH estimates from gorillas[158] and Human Genome Diversity Project (*HGDP*)[154].

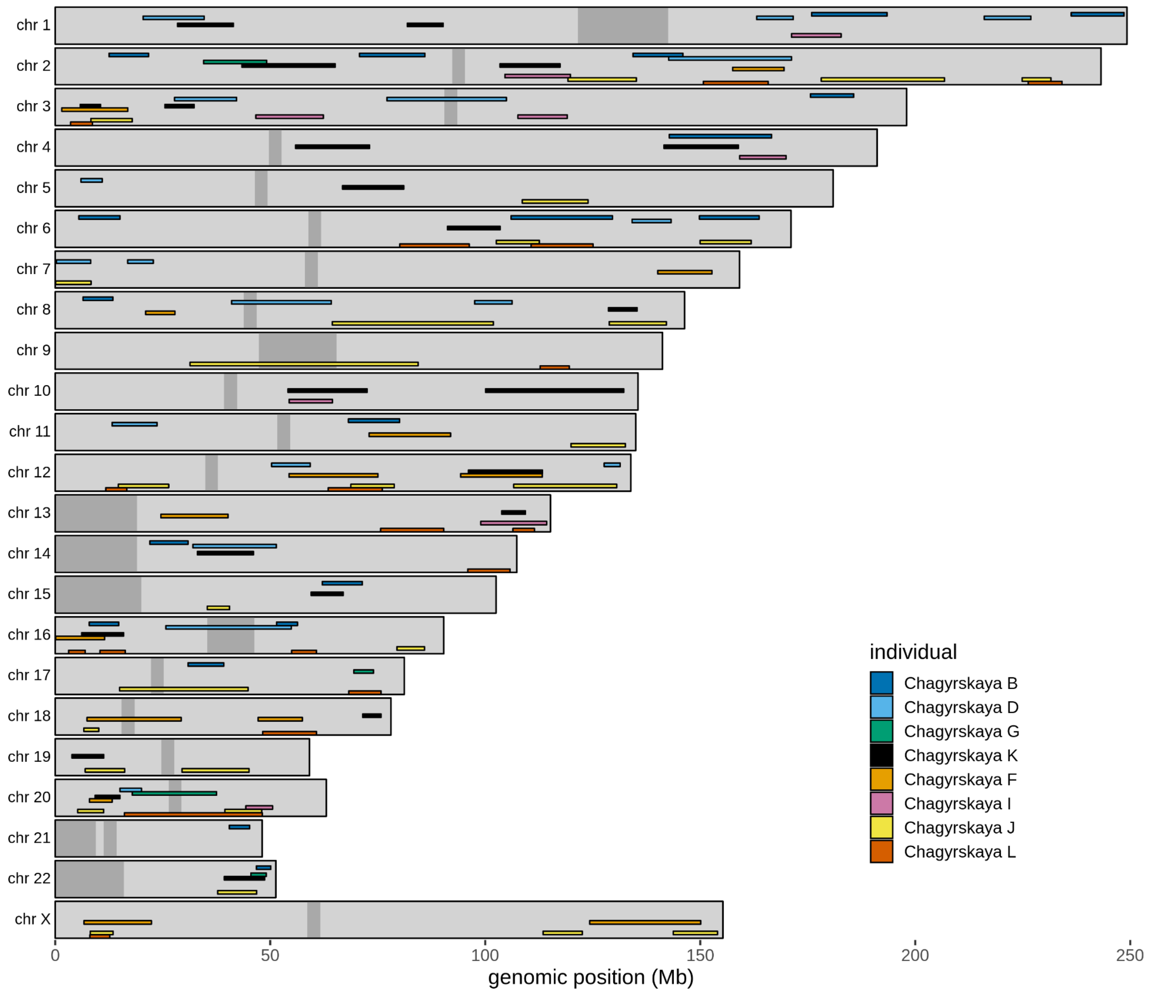

SI Figure 9.2. The genomic locations of all ROH longer than 10cM for 8 Chagyrskaya individuals. *Chagyrskaya B, D, G* and *K* are males; *Chagyrskaya F, I, J* and *L* are female.

SI 10 Diversity ratio of X chromosome and autosomes

Under neutrality the X chromosome diversity to autosome diversity ratio should be 0.75 because there are 3 X chromosomes for each 4 autosomes in a population of equal number of men and women. However in recent years it has been shown to be different from this expectation value in modern human populations and this can be a result of multiple different processes..

i) Differences in mutation rates between X and the autosomes
ii) Sex-biased demographic events or social organization (such as male philopatry)
iii) changes in historical N_e over time, such as population bottlenecks which would affect the X chromosome more than the autosomes.
iv) Types of selection that differentially affecting X and the autosomes

In this section we explore the X diversity/Autosome diversity difference in the Chagyrskaya and Okladnikov individuals - in particular we will try to correct for factors i) and iv) to investigate if there is evidence of Sex-biased demographic events .

To investigate the diversity ratio on the X chromosome and autosomes we remove a set of captured variants which could be functional and thus be under some form of selection[175]. The purpose of these filters is to limit the signal coming from iv) by restricting to putative neutral regions. The filters are:

1) Variants are more than 2 kb from the midpoint of exons (Downloaded from UCSC table browser - hg19 build)[103].
2) Have a phastCons100-vertebrate alignment score lower than 0[176]
3) Are in regions with a B-value[177] between 0.6 and 0.9

After these filters we are left with 2,246 sites on the X chromosome and 133,437 sites on the autosomes in the capture array.

When calculating the diversity for the Chagyrskaya and Okladnikov we removed *Chagyrskaya D* due to large amounts of non-human contamination (see **SI 5 Other forms of contamination**).

We calculate diversity (π) at each site as 2 * P * Q / (n * (n - 1)) where P is the couts of the reference allele, Q is the count of the alternative allele and n = P + Q.

To take the difference in male and female mutation rate into account we use a male-to-female mutation rate of alpha = 4.25 which is estimated from pedigree data[175]**.**

Biased due to capture array

Because we are selecting for variants that are polymorphic in Neanderthals and Denisovans one potential issue is that we overestimate the amount of heterozygosity on the autosomes and the X chromosome. In order to see the effect of ascertaining sites we calculate the X diversity / autosome diversity for the 4 high coverage archaic individuals both using the captured sites and the whole genome sequence.

In addition we also remove sites that are in runs of homozygosity longer than 2.5 cM.

In order to calculate confidence intervals we perform 100 bootstrap samples with replacement of the autosome and X chromosome diversity and then calculate the X/autosome diversity ratio. We show the results below in **SI Table 10.1** and visually in **SI Figure 10.1**.

		Autosomes		X chromosomes		X/autosome diversity		
**Data**	**Individual/pop**	**sites available**	**hets per site**	**sites available**	**hets per site**	**Mean value**	**min 95% CI**	**max 95% CI**
**Whole genome**	Vindija 33.19	287,367,449	1.7132	6,020,873	1.131	0.832	0.67	1.004
	Denisova 5 (Altai)	256,073,752	2.0155	5,490,314	1.752	1.095	0.757	1.48
	Denisova 3	302,846,833	1.9489	8,053,913	1.042	0.674	0.542	0.798
	Chagyrskaya 8	254,898,847	1.7258	3,847,263	1.076	0.786	0.585	1.025
**Capture array**	Vindija 33.19	124,335	0.046	1,631	0.042	1.141	0.884	1.415
	Denisova 5 (Altai)	110,114	0.052	1,521	0.047	1.126	0.915	1.376
	Denisova 3	131,000	0.05	2,169	0.042	1.045	0.832	1.312
	Chagyrskaya 8	110,521	0.047	1,043	0.035	0.929	0.63	1.202
	Chagyrskayas	409,551	0.038	10,822	0.029	0.981	0.914	1.049
	Okladnikovs	2,774	0.052	30	0.1	2.379	0	4.618

SI Table 10.1. We show the number sites available for analysis after filtering for the autosomes and the X chromosome. We show the number of heterozygous sites per called site. We show the X/autosome diversity ratio - corrected for difference in male and female mutation rate along with 95% confidence intervals.

Note that the X/Autosome diversity increases when we only use sites from the capture array. The effect is more extreme for Denisova 3 which could be explained by the fact that we use 3 Neanderthals but only one Denisovan for designing the capture array.

Thus the X/autosome ratio of 0.981 for the Chagyrskaya community is likely overestimated. If we assume it is overestimated by the same amount as the X/autosome diversity in *Chagyrskaya 8* the actual value is likely not very different from 0.75

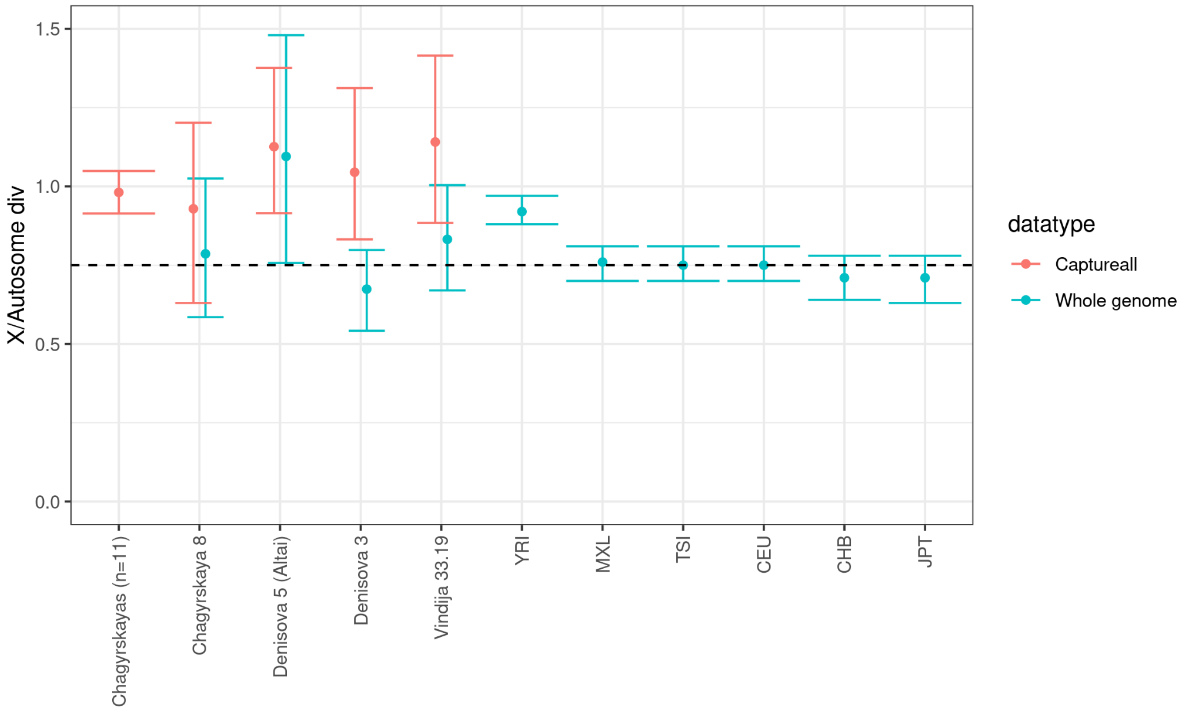

SI Figure 10.1. We show the X to autosome diversity for the individuals from Chagyrskaya cave along with the four high coverage archaics and 6 contemporary human populations (values taken from[175]). Mid-points are means and error bars are 95% CI from 100 bootstrap iterations.

References

1. Kolobova, K. A. *et al.* Archaeological evidence for two separate dispersals of Neanderthals into southern Siberia. *Proc. Natl. Acad. Sci. U. S. A.* **117**, 2879–2885 (2020).
2. Mafessoni, F. *et al.* A high-coverage Neandertal genome from Chagyrskaya Cave. *Proc. Natl. Acad. Sci. U. S. A.* **117**, 15132–15136 (2020).
3. Derevianko, A. P. *et al. Multidisciplinary studies of chagyrskaya cave – A middle paleolithic site in Altai*. (IAET SB RAS Publishing, 2018).
4. Kolobova, K. A. *et al.* Exploitation of the natural environment by Neanderthals from Chagyrskaya Cave (Altai). *qu* **66**, 7–31 (2019).
5. Vernot, B. *et al.* Unearthing Neanderthal population history using nuclear and mitochondrial DNA from cave sediments. *Science* eabf1667 (2021).
6. Galbraith, R. F. & Roberts, R. G. Statistical aspects of equivalent dose and error calculation and display in OSL dating: An overview and some recommendations. *Quaternary Geochronology* vol. 11 1–27 (2012).
7. Talamo, S. & Richards, M. A Comparison of Bone Pretreatment Methods for AMS Dating of Samples >30,000 BP. *Radiocarbon* vol. 53 443–449 (2011).
8. Talamo, S., Fewlass, H., Maria, R. & Jaouen, K. 'Here we go again': the inspection of collagen extraction protocols for 14C dating and palaeodietary analysis. *STAR: Science & Technology of Archaeological Research* vol. 7 62–77 (2021).
9. Kromer, B., Lindauer, S., Synal, H.-A. & Wacker, L. MAMS – A new AMS facility at the Curt-Engelhorn-Centre for Achaeometry, Mannheim, Germany. *Nucl. Instrum. Methods Phys. Res. B* **294**, 11–13 (2013).
10. Petr, M. *et al.* The evolutionary history of Neanderthal and Denisovan Y chromosomes.

Science **369**, 1653–1656 (2020).

11. Baumann, M. *et al.* The Neandertal bone industry at Chagyrskaya cave, Altai Region, Russia. *Quaternary International* vol. 559 68–88 (2020).
12. Derevianko, A. P., Markin S. V. *Mustye Gornogo Altaya [The Mousterian of the Mountainous Altai]*. (Novosibirsk: Nauka., 1992).
13. Derevianko, A. P. & Markin, S. The Paleolithic of Siberia: new discoveries and interpretations. 94–97 (1998).
14. Derevianko, A. P. *et al.* Archaeology, Geology, and the Pleistocene and Holocene Palaeogeography of the Mountainous Altai. (1998).
15. Turner, C. G., II, Ovodov, N. D. & Pavlova, O. V. *Animal Teeth and Human Tools: A Taphonomic Odyssey in Ice Age Siberia*. (Cambridge University Press, 2013).
16. Derevianko, A. P., Markin S. V. *Mustye Gornogo Altaya [The Mousterian of the Mountainous Altai]*. (Novosibirsk: Nauka., 1992).
17. Krause, J. *et al.* Neanderthals in central Asia and Siberia. *Nature* **449**, 902–904 (2007).
18. Higham, T. F. G. Removing contaminants: a restatement of the value of isolating single compounds for AMS dating. *Antiquity* vol. 93 1072–1075 (2019).
19. Deviese, T., Comeskey, D., McCullagh, J., Bronk Ramsey, C. & Higham, T. New protocol for compound-specific radiocarbon analysis of archaeological bones. *Rapid Commun. Mass Spectrom.* **32**, 373–379 (2018).
20. Mednikova, M. B. Postcranial morphology and taxonomy of genus Homo representatives from Okladnikov Cave in Altai. (2011).
21. Goebel, F. E. The Middle to Upper Paleolithic Transition in Siberia. (1994).
22. Grün, R., Eggins, S., Kinsley, L., Moseley, H. & Sambridge, M. Laser ablation U-series analysis of fossil bones and teeth. *Palaeogeography, Palaeoclimatology, Palaeoecology* vol. 416 150–167 (2014).
23. Kharevich, A. V. *et al.* New archaeological sites in the northwestern Altai

(Krasnoschekovsky and Soloneshensky Districts of Altai Krai). (2020).

24. Derevianko, A. P., Markin, S. V. & Shunkov, M. V. The Sibiryachikha Facies of the Middle Paleolithic of the Altai. *Archaeology, Ethnology and Anthropology of Eurasia* vol. 41 89–103 (2013).
25. Smith, B. H. Standards of human tooth formation and dental age assessment. (1991).
26. Hillson, S. *Dental Anthropology*. (Cambridge University Press, 1996).
27. Moorrees, C. F., Fanning, E. A. & Hunt, E. E., Jr. AGE VARIATION OF FORMATION STAGES FOR TEN PERMANENT TEETH. *J. Dent. Res.* **42**, 1490–1502 (1963).
28. Shackelford, L. L., Stinespring Harris, A. E. & Konigsberg, L. W. Estimating the distribution of probable age-at-death from dental remains of immature human fossils. *Am. J. Phys. Anthropol.* **147**, 227–253 (2012).
29. Liversidge, H. Variation in modern human dental development. in *Patterns of Growth and Development in the Genus Homo* (eds. Thompson, J. L., Krovitz, G. E. & Nelson, A. J.) 73–113 (Cambridge University Press, 2003).
30. Macchiarelli, R. *et al.* How Neanderthal molar teeth grew. *Nature* **444**, 748–751 (2006).
31. Smith, T. M. *et al.* Dental evidence for ontogenetic differences between modern humans and Neanderthals. *Proc. Natl. Acad. Sci. U. S. A.* **107**, 20923–20928 (2010).
32. Smith, T. M. *et al. The Scladina I-4A Juvenile Neandertal Andenne, Belgium: Palaeoanthropology and Context*. (2014).
33. Molnar, S. Human tooth wear, tooth function and cultural variability. *Am. J. Phys. Anthropol.* **34**, 175–189 (1971).
34. Wolpoff, M. H. The Krapina dental remains. *Am. J. Phys. Anthropol.* **50**, 67–114 (1978).
35. Molnar, S. *et al.* Tooth Wear and Culture: A Survey of Tooth Functions Among Some Prehistoric Populations [and Comments and Reply]. *Current Anthropology* vol. 13 511–526 (1972).
36. Smith, P. Dental Pathology in Fossil Hominids: What Did Neanderthals Do With Their

Teeth? *Current Anthropology* vol. 17 149–151 (1976).

37. Hillson, S. *Tooth Development in Human Evolution and Bioarchaeology*. (Cambridge University Press, 2014).
38. Scheuer, L. *Developmental Juvenile Osteology: Louise Scheuer, Sue Black*. (2000).
39. Mednikova, M. B. An Archaic Human Ulna from Chagyrskaya Cave Altai: Morphology and Taxonomy*. *Archaeology, Ethnology and Anthropology of Eurasia* vol. 41 66–77 (2013).
40. Haavikko, K. *The Formation and the Alveolar and Clinical Eruption of the Permanent Teeth: An Orthopantomographic Study*. (1970).
41. Wallace, J. A. *Did La Ferrassie I Use His Teeth as a Tool*. (1975).
42. Trinkaus, E. *The Shanidar Neandertals*. (1983).
43. Hassanali, J. & Odhiambo, J. W. Ages of eruption of the permanent teeth in Kenyan African and asian children. *Ann. Hum. Biol.* **8**, 425–434 (1981).
44. Hassanali, J. The third permanent molar eruption in Kenyan Africans and Asians. *Ann. Hum. Biol.* **12**, 517–523 (1985).
45. Smith, T. M. *et al.* Dental Development and Age at Death of a Middle Paleolithic Juvenile Hominin from Obi-Rakhmat Grotto, Uzbekistan. *Vertebrate Paleobiology and Paleoanthropology* 155–163 (2011) doi:10.1007/978-94-007-0492-3_13.
46. Glantz, M. *et al.* New hominin remains from Uzbekistan. *J. Hum. Evol.* **55**, 223–237 (2008).
47. Mednikova, M. B. A proximal pedal phalanx of a Paleolithic hominin from denisova cave, Altai. *Archaeology, Ethnology and Anthropology of Eurasia* vol. 39 129–138 (2011).
48. Glantz, M., Athreya, S. & Ritzman, T. Is Central Asia the eastern outpost of the Neandertal range? A reassessment of the Teshik-Tash child. *Am. J. Phys. Anthropol.* **138**, 45–61 (2009).
49. Schultz, M. Light Microscopic Analysis in Skeletal Paleopathology. *Identification of*

Pathological Conditions in Human Skeletal Remains 73–II (2003) doi:10.1016/b978-012528628-2/50043-0.

50. Viola, B. T. New hominid remains from Central Asia and Siberia: the Easternmost Neanderthals? (2009).
51. Skoglund, P. *et al.* Separating endogenous ancient DNA from modern day contamination in a Siberian Neandertal. *Proceedings of the National Academy of Sciences* vol. 111 2229–2234 (2014).
52. Korlević, P. *et al.* Reducing microbial and human contamination in DNA extractions from ancient bones and teeth. *Biotechniques* **59**, 87–93 (2015).
53. Rohland, N., Glocke, I., Aximu-Petri, A. & Meyer, M. Extraction of highly degraded DNA from ancient bones, teeth and sediments for high-throughput sequencing. *Nat. Protoc.* **13**, 2447–2461 (2018).
54. Gansauge, M.-T. & Meyer, M. Single-stranded DNA library preparation for the sequencing of ancient or damaged DNA. *Nat. Protoc.* **8**, 737–748 (2013).
55. Gansauge, M.-T., Aximu-Petri, A., Nagel, S. & Meyer, M. Manual and automated preparation of single-stranded DNA libraries for the sequencing of DNA from ancient biological remains and other sources of highly degraded DNA. *Nat. Protoc.* **15**, 2279–2300 (2020).
56. Glocke, I. & Meyer, M. Extending the spectrum of DNA sequences retrieved from ancient bones and teeth. *Genome Res.* **27**, 1230–1237 (2017).
57. Kircher, M., Sawyer, S. & Meyer, M. Double indexing overcomes inaccuracies in multiplex sequencing on the Illumina platform. *Nucleic Acids Res.* **40**, e3 (2012).
58. Damgaard, P. B. *et al.* Improving access to endogenous DNA in ancient bones and teeth. *Sci. Rep.* **5**, 11184 (2015).
59. Renaud, G., Stenzel, U. & Kelso, J. leeHom: adaptor trimming and merging for Illumina sequencing reads. *Nucleic Acids Res.* **42**, e141 (2014).

60. Li, H. & Durbin, R. Fast and accurate long-read alignment with Burrows-Wheeler transform. *Bioinformatics* **26**, 589–595 (2010).

61. Briggs, A. W. *et al.* Patterns of damage in genomic DNA sequences from a Neandertal. *Proc. Natl. Acad. Sci. U. S. A.* **104**, 14616–14621 (2007).

62. Maricic, T., Whitten, M. & Pääbo, S. Multiplexed DNA sequence capture of mitochondrial genomes using PCR products. *PLoS One* **5**, e14004 (2010).

63. Fu, Q. *et al.* A revised timescale for human evolution based on ancient mitochondrial genomes. *Curr. Biol.* **23**, 553–559 (2013).

64. Slon, V. *et al.* Neandertal and Denisovan DNA from Pleistocene sediments. *Science* **356**, 605–608 (2017).

65. Andrews, R. M. *et al.* Reanalysis and revision of the Cambridge reference sequence for human mitochondrial DNA. *Nat. Genet.* **23**, 147 (1999).

66. Fu, Q. *et al.* DNA analysis of an early modern human from Tianyuan Cave, China. *Proc. Natl. Acad. Sci. U. S. A.* **110**, 2223–2227 (2013).

67. Green, R. E. *et al.* A complete Neandertal mitochondrial genome sequence determined by high-throughput sequencing. *Cell* **134**, 416–426 (2008).

68. Briggs, A. W. *et al.* Targeted retrieval and analysis of five Neandertal mtDNA genomes. *Science* **325**, 318–321 (2009).

69. Brown, S. *et al.* Identification of a new hominin bone from Denisova Cave, Siberia using collagen fingerprinting and mitochondrial DNA analysis. *Sci. Rep.* **6**, 23559 (2016).

70. Douka, K. *et al.* Age estimates for hominin fossils and the onset of the Upper Palaeolithic at Denisova Cave. *Nature* **565**, 640–644 (2019).

71. Gansauge, M.-T. & Meyer, M. Selective enrichment of damaged DNA molecules for ancient genome sequencing. *Genome Res.* **24**, 1543–1549 (2014).

72. Hajdinjak, M. *et al.* Reconstructing the genetic history of late Neanderthals. *Nature* **555**, 652–656 (2018).

73. Peyrégne, S. *et al.* Nuclear DNA from two early Neandertals reveals 80,000 years of genetic continuity in Europe. *Sci Adv* **5**, eaaw5873 (2019).
74. Posth, C. *et al.* Deeply divergent archaic mitochondrial genome provides lower time boundary for African gene flow into Neanderthals. *Nat. Commun.* **8**, 16046 (2017).
75. Prüfer, K. *et al.* The complete genome sequence of a Neanderthal from the Altai Mountains. *Nature* **505**, 43–49 (2014).
76. Prüfer, K. *et al.* A high-coverage Neandertal genome from Vindija Cave in Croatia. *Science* **358**, 655–658 (2017).
77. Rougier, H. *et al.* Neandertal cannibalism and Neandertal bones used as tools in Northern Europe. *Sci. Rep.* **6**, 29005 (2016).
78. Altschul, S. F., Gish, W., Miller, W., Myers, E. W. & Lipman, D. J. Basic local alignment search tool. *J. Mol. Biol.* **215**, 403–410 (1990).
79. Sawyer, S. *Insights Into Neandertals and Denisovans Fron Denisova Cave*. (2016).
80. Payne, B. A. I. *et al.* Universal heteroplasmy of human mitochondrial DNA. *Hum. Mol. Genet.* **22**, 384–390 (2013).
81. Laricchia, K. M. *et al.* Mitochondrial DNA variation across 56,434 individuals in gnomAD. doi:10.1101/2021.07.23.453510.
82. Wei, W. *et al.* Germline selection shapes human mitochondrial DNA diversity. *Science* **364**, (2019).
83. Liu, C. *et al.* Presence and Transmission of Mitochondrial Heteroplasmic Mutations in Human Populations of European and African Ancestry. doi:10.1101/2020.10.13.337071.
84. Posth, C. *et al.* Pleistocene Mitochondrial Genomes Suggest a Single Major Dispersal of Non-Africans and a Late Glacial Population Turnover in Europe. *Curr. Biol.* **26**, 827–833 (2016).
85. Reimer, P. J. *et al.* The IntCal20 Northern Hemisphere Radiocarbon Age Calibration Curve (0–55 cal kBP). *Radiocarbon* **62**, 725–757 (2020).

86. Fu, Q. *et al.* Genome sequence of a 45,000-year-old modern human from western Siberia. *Nature* **514**, 445–449 (2014).
87. Krause, J. *et al.* The complete mitochondrial DNA genome of an unknown hominin from southern Siberia. *Nature* **464**, 894–897 (2010).
88. Gilbert, M. T. P. *et al.* DNA from pre-Clovis human coprolites in Oregon, North America. *Science* **320**, 786–789 (2008).
89. Ermini, L. *et al.* Complete mitochondrial genome sequence of the Tyrolean Iceman. *Curr. Biol.* **18**, 1687–1693 (2008).
90. Reich, D. *et al.* Genetic history of an archaic hominin group from Denisova Cave in Siberia. *Nature* **468**, 1053–1060 (2010).
91. Sawyer, S. *et al.* Nuclear and mitochondrial DNA sequences from two Denisovan individuals. *Proc. Natl. Acad. Sci. U. S. A.* **112**, 15696–15700 (2015).
92. Slon, V. *et al.* A fourth Denisovan individual. *Sci Adv* **3**, e1700186 (2017).
93. Meyer, M. *et al.* A mitochondrial genome sequence of a hominin from Sima de los Huesos. *Nature* vol. 505 403–406 (2014).
94. Horai, S., Hayasaka, K., Kondo, R., Tsugane, K. & Takahata, N. Recent African origin of modern humans revealed by complete sequences of hominoid mitochondrial DNAs. *Proc. Natl. Acad. Sci. U. S. A.* **92**, 532–536 (1995).
95. Katoh, K. & Standley, D. M. MAFFT multiple sequence alignment software version 7: improvements in performance and usability. *Mol. Biol. Evol.* **30**, 772–780 (2013).
96. Bouckaert, R. *et al.* BEAST 2.5: An advanced software platform for Bayesian evolutionary analysis. *PLoS Comput. Biol.* **15**, e1006650 (2019).
97. Leaché, A. D., Fujita, M. K., Minin, V. N. & Bouckaert, R. R. Species delimitation using genome-wide SNP data. *Syst. Biol.* **63**, 534–542 (2014).
98. Tamura, K. & Nei, M. Estimation of the number of nucleotide substitutions in the control region of mitochondrial DNA in humans and chimpanzees. *Mol. Biol. Evol.* **10**, 512–526

(1993).

99. Rambaut, A., Drummond, A. J., Xie, D., Baele, G. & Suchard, M. A. Posterior Summarization in Bayesian Phylogenetics Using Tracer 1.7. *Systematic Biology* vol. 67 901–904 (2018).

100. Tavaré, S., Balding, D. J., Griffiths, R. C. & Donnelly, P. Inferring coalescence times from DNA sequence data. *Genetics* **145**, 505–518 (1997).

101. Fenner, J. N. Cross-cultural estimation of the human generation interval for use in genetics-based population divergence studies. *Am. J. Phys. Anthropol.* **128**, 415–423 (2005).

102. Skaletsky, H. *et al.* The male-specific region of the human Y chromosome is a mosaic of discrete sequence classes. *Nature* **423**, 825–837 (2003).

103. Karolchik, D. *et al.* The UCSC Table Browser data retrieval tool. *Nucleic Acids Res.* **32**, D493–6 (2004).

104. Mallick, S. *et al.* The Simons Genome Diversity Project: 300 genomes from 142 diverse populations. *Nature* **538**, 201–206 (2016).

105. Karmin, M. *et al.* A recent bottleneck of Y chromosome diversity coincides with a global change in culture. *Genome Res.* **25**, 459–466 (2015).

106. Mendez, F. L. *et al.* An African American Paternal Lineage Adds an Extremely Ancient Root to the Human Y Chromosome Phylogenetic Tree. *The American Journal of Human Genetics* vol. 92 454–459 (2013).

107. Hallast, P. *et al.* The Y-chromosome tree bursts into leaf: 13,000 high-confidence SNPs covering the majority of known clades. *Mol. Biol. Evol.* **32**, 661–673 (2015).

108. Wilson, E. B. Probable Inference, the Law of Succession, and Statistical Inference. *Journal of the American Statistical Association* vol. 22 209–212 (1927).

109. Wallis, S. Binomial Confidence Intervals and Contingency Tests: Mathematical Fundamentals and the Evaluation of Alternative Methods. *Journal of Quantitative*

Linguistics vol. 20 178–208 (2013).

110. Renaud, G., Hanghøj, K., Willerslev, E. & Orlando, L. gargammel: a sequence simulator for ancient DNA. *Bioinformatics* **33**, 577–579 (2017).

111. Meyer, M. *et al.* A high-coverage genome sequence from an archaic Denisovan individual. *Science* **338**, 222–226 (2012).

112. Skov, L., Danish Pan Genome Consortium & Schierup, M. H. Analysis of 62 hybrid assembled human Y chromosomes exposes rapid structural changes and high rates of gene conversion. *PLoS Genet.* **13**, e1006834 (2017).

113. Massaia, A. & Xue, Y. Human Y chromosome copy number variation in the next generation sequencing era and beyond. *Hum. Genet.* **136**, 591–603 (2017).

114. Wei, W. *et al.* Copy number variation in the human Y chromosome in the UK population. *Hum. Genet.* **134**, 789–800 (2015).

115. Repping, S. *et al.* Polymorphism for a 1.6-Mb deletion of the human Y chromosome persists through balance between recurrent mutation and haploid selection. *Nat. Genet.* **35**, 247–251 (2003).

116. Poznik, G. D. *et al.* Punctuated bursts in human male demography inferred from 1,244 worldwide Y-chromosome sequences. *Nat. Genet.* **48**, 593–599 (2016).

117. Shi, W. *et al.* Birth, expansion, and death of VCY-containing palindromes on the human Y chromosome. *Genome Biol.* **20**, 207 (2019).

118. Hallast, P. *et al.* Great ape Y Chromosome and mitochondrial DNA phylogenies reflect subspecies structure and patterns of mating and dispersal. *Genome Res.* **26**, 427–439 (2016).

119. Yong, R. Y. Y., Gan, L. S. H., Chang, Y. M. & Yap, E. P. H. Molecular characterization of a polymorphic 3-Mb deletion at chromosome Yp11.2 containing the AMELY locus in Singapore and Malaysia populations. *Hum. Genet.* **122**, 237–249 (2007).

120. Welker, F. Palaeoproteomics for human evolution studies. *Quaternary Science Reviews*

vol. 190 137–147 (2018).

121. Peyrégne, S. & Prüfer, K. Present-Day DNA Contamination in Ancient DNA Datasets. *Bioessays* **42**, e2000081 (2020).

122. Krause, J. *et al.* A complete mtDNA genome of an early modern human from Kostenki, Russia. *Curr. Biol.* **20**, 231–236 (2010).

123. Navarro Gonzalez, J. *et al.* The UCSC Genome Browser database: 2021 update. *Nucleic Acids Res.* **49**, D1046–D1057 (2021).

124. Mendez, F. L., Poznik, G. D., Castellano, S. & Bustamante, C. D. The Divergence of Neandertal and Modern Human Y Chromosomes. *Am. J. Hum. Genet.* **98**, 728–734 (2016).

125. Consortium, T. 1000 G. P. & The 1000 Genomes Project Consortium. A global reference for human genetic variation. *Nature* vol. 526 68–74 (2015).

126. Patterson, N. J. *et al.* Ancient Admixture in Human History. *Genetics* genetics.112.145037 (2012).

127. Peter, B. M. Admixture, Population Structure, and F-Statistics. *Genetics* **202**, 1485–1501 (2016).

128. Petr, M., Vernot, B. & Kelso, J. admixr—R package for reproducible analyses using ADMIXTOOLS. *Bioinformatics* **35**, 3194–3195 (2019).

129. Peter, B. M. 100,000 years of gene flow between Neandertals and Denisovans in the Altai mountains. *bioRxiv* 2020.03.13.990523 (2020) doi:10.1101/2020.03.13.990523.

130. Hinch, A. G. *et al.* The landscape of recombination in African Americans. *Nature* **476**, 170–175 (2011).

131. Huerta-Sánchez, E. *et al.* Altitude adaptation in Tibetans caused by introgression of Denisovan-like DNA. *Nature* **512**, 194–197 (2014).

132. Monroy Kuhn, J. M., Jakobsson, M. & Günther, T. Estimating genetic kin relationships in prehistoric populations. *PLoS One* **13**, e0195491 (2018).

133. Oota, H., Settheetham-Ishida, W., Tiwawech, D., Ishida, T. & Stoneking, M. Human mtDNA and Y-chromosome variation is correlated with matrilocal versus patrilocal residence. *Nat. Genet.* **29**, 20–21 (2001).

134. Seielstad, M. T., Minch, E. & Cavalli-Sforza, L. L. Genetic evidence for a higher female migration rate in humans. *Nat. Genet.* **20**, 278–280 (1998).

135. Singleton, I. & van Schaik, C. P. The social organisation of a population of Sumatran orang-utans. *Folia Primatol.* **73**, 1–20 (2002).

136. Nater, A. *et al.* Sex-biased dispersal and volcanic activities shaped phylogeographic patterns of extant Orangutans (genus: Pongo). *Mol. Biol. Evol.* **28**, 2275–2288 (2011).

137. Goossens, B. *et al.* Philopatry and reproductive success in Bornean orang-utans (Pongo pygmaeus). *Mol. Ecol.* **15**, 2577–2588 (2006).

138. van Noordwijk, M. A. *et al.* Female philopatry and its social benefits among Bornean orangutans. *Behav. Ecol. Sociobiol.* **66**, 823–834 (2012).

139. Nietlisbach, P. *et al.* Heavily male-biased long-distance dispersal of orang-utans (genus: Pongo), as revealed by Y-chromosomal and mitochondrial genetic markers. *Mol. Ecol.* **21**, 3173–3186 (2012).

140. Vigilant, L. *et al.* Reproductive competition and inbreeding avoidance in a primate species with habitual female dispersal. *Behav. Ecol. Sociobiol.* **69**, 1163–1172 (2015).

141. Harcourt, A. H. & Stewart, K. J. Gorilla society: What we know and don't know. *Evol. Anthropol.* **16**, 147–158 (2007).

142. Bradley, B. J., Doran-Sheehy, D. M. & Vigilant, L. Potential for female kin associations in wild western gorillas despite female dispersal. *Proc. Biol. Sci.* **274**, 2179–2185 (2007).

143. Parnell, R. J. Group size and structure in western lowland gorillas (Gorilla gorilla gorilla) at Mbeli Bai, Republic of Congo. *Am. J. Primatol.* **56**, 193–206 (2002).

144. Robbins, A. M., Stoinski, T. S., Fawcett, K. A. & Robbins, M. M. Socioecological influences on the dispersal of female mountain gorillas—evidence of a second folivore

paradox. *Behav. Ecol. Sociobiol.* **63**, 477–489 (2009).

145. Douadi, M. I. *et al.* Sex-biased dispersal in western lowland gorillas (Gorilla gorilla gorilla). *Mol. Ecol.* **16**, 2247–2259 (2007).

146. Langergraber, K. E., Mitani, J. C. & Vigilant, L. The limited impact of kinship on cooperation in wild chimpanzees. *Proc. Natl. Acad. Sci. U. S. A.* **104**, 7786–7790 (2007).

147. Nishida, T. *et al.* Demography, female life history, and reproductive profiles among the chimpanzees of Mahale. *Am. J. Primatol.* **59**, 99–121 (2003).

148. McCarthy, M. S. *et al.* Genetic censusing identifies an unexpectedly sizeable population of an endangered large mammal in a fragmented forest landscape. *BMC Ecol.* **15**, 21 (2015).

149. Schubert, G. *et al.* Male-mediated gene flow in patrilocal primates. *PLoS One* **6**, e21514 (2011).

150. Lukas, D., Reynolds, V., Boesch, C. & Vigilant, L. To what extent does living in a group mean living with kin? *Mol. Ecol.* **14**, 2181–2196 (2005).

151. Hohmann, G. Association and social interactions between strangers and residents in bonobos (Pan paniscus). *Primates* **42**, 91–99 (2001).

152. Eriksson, J. *et al.* Y-chromosome analysis confirms highly sex-biased dispersal and suggests a low male effective population size in bonobos (Pan paniscus). *Mol. Ecol.* **15**, 939–949 (2006).

153. Lippold, S. *et al.* Human paternal and maternal demographic histories: insights from high-resolution Y chromosome and mtDNA sequences. *Investig. Genet.* **5**, 13 (2014).

154. Bergström, A. *et al.* Insights into human genetic variation and population history from 929 diverse genomes. *Science* **367**, (2020).

155. Hajdinjak, M. *et al.* Initial Upper Palaeolithic humans in Europe had recent Neanderthal ancestry. *Nature* **592**, 253–257 (2021).

156. Sikora, M. *et al.* Ancient genomes show social and reproductive behavior of early Upper Paleolithic foragers. *Science* **358**, 659–662 (2017).

157. Fu, Q. *et al.* The genetic history of Ice Age Europe. *Nature* **534**, 200–205 (2016).

158. Xue, Y. *et al.* Mountain gorilla genomes reveal the impact of long-term population decline and inbreeding. *Science* **348**, 242–245 (2015).

159. Prado-Martinez, J. *et al.* Great ape genetic diversity and population history. *Nature* **499**, 471–475 (2013).

160. Rebolledo-Jaramillo, B. *et al.* Maternal age effect and severe germ-line bottleneck in the inheritance of human mitochondrial DNA. *Proc. Natl. Acad. Sci. U. S. A.* **111**, 15474–15479 (2014).

161. Soares, P. *et al.* Correcting for purifying selection: an improved human mitochondrial molecular clock. *Am. J. Hum. Genet.* **84**, 740–759 (2009).

162. Zaidi, A. A. *et al.* Bottleneck and selection in the germline and maternal age influence transmission of mitochondrial DNA in human pedigrees. *Proc. Natl. Acad. Sci. U. S. A.* **116**, 25172–25178 (2019).

163. Rieux, A. *et al.* Improved calibration of the human mitochondrial clock using ancient genomes. *Mol. Biol. Evol.* **31**, 2780–2792 (2014).

164. Brotherton, P. *et al.* Neolithic mitochondrial haplogroup H genomes and the genetic origins of Europeans. *Nat. Commun.* **4**, 1764 (2013).

165. Francalacci, P. *et al.* Low-pass DNA sequencing of 1200 Sardinians reconstructs European Y-chromosome phylogeny. *Science* **341**, 565–569 (2013).

166. Balanovsky, O. *et al.* Deep phylogenetic analysis of haplogroup G1 provides estimates of SNP and STR mutation rates on the human Y-chromosome and reveals migrations of Iranic speakers. *PLoS One* **10**, e0122968 (2015).

167. Poznik, G. D. *et al.* Sequencing Y chromosomes resolves discrepancy in time to common ancestor of males versus females. *Science* **341**, 562–565 (2013).

168. Helgason, A. *et al.* The Y-chromosome point mutation rate in humans. *Nature Genetics* vol. 47 453–457 (2015).

169. Xue, Y. *et al.* Human Y Chromosome Base-Substitution Mutation Rate Measured by Direct Sequencing in a Deep-Rooting Pedigree. *Current Biology* vol. 19 1453–1457 (2009).

170. Skov, L. *et al.* The nature of Neanderthal introgression revealed by 27,566 Icelandic genomes. *Nature* (2020) doi:10.1038/s41586-020-2225-9.

171. Duveau, J., Berillon, G., Verna, C., Laisné, G. & Cliquet, D. The composition of a Neandertal social group revealed by the hominin footprints at Le Rozel (Normandy, France). *Proc. Natl. Acad. Sci. U. S. A.* **116**, 19409–19414 (2019).

172. Mayoral, E. *et al.* Tracking late Pleistocene Neandertals on the Iberian coast. *Sci. Rep.* **11**, 4103 (2021).

173. Hayden, B. Neandertal social structure? *Oxf. j. archaeol.* **31**, 1–26 (2012).

174. Lalueza-Fox, C. *et al.* Genetic evidence for patrilocal mating behavior among Neandertal groups. *Proc. Natl. Acad. Sci. U. S. A.* **108**, 250–253 (2011).

175. Amster, G., Murphy, D. A., Milligan, W. R. & Sella, G. Changes in life history and population size can explain the relative neutral diversity levels on X and autosomes in extant human populations. *Proc. Natl. Acad. Sci. U. S. A.* **117**, 20063–20069 (2020).

176. Siepel, A. *et al.* Evolutionarily conserved elements in vertebrate, insect, worm, and yeast genomes. *Genome Res.* **15**, 1034–1050 (2005).

177. McVicker, G., Gordon, D., Davis, C. & Green, P. Widespread genomic signatures of natural selection in hominid evolution. *PLoS Genet.* **5**, e1000471 (2009).
